# Supplementary material for: Effect of switching from nucleos(t)ide maintenance therapy to PegIFN alfa-2a in patients with HBeAg-positive chronic hepatitis B: A randomized trial
Source: PLoS One. 2022 Jul 22;17(7):e0270716. doi: 10.1371/journal.pone.0270716 (PMC9307167; doi:10.1371/journal.pone.0270716)
Supplement: S1 Table — (DOCX) [file pone.0270716.s002.docx]

**S1 Table. Outcome variables at each assessment time in the two groups.**

|  | **Group** | |  | **Analysis for repeated measures** | |
| --- | --- | --- | --- | --- | --- |
| **Variable** | **PegIFNα-2a**  **(n=75)** | **NA**  **(n=74)** | **p** | **Source** | **p** |
| **HBsAg (log_10_IU/mL)** |  |  |  |  |  |
| baseline | 3.50±0.55^a*^ | 3.49±0.51^a^ | 0.962 | **Group** | 0.515^‡^ |
| 12 weeks | 3.41±0.70^a^ | 3.44±0.53^a^ | 0.938 | **Time** | 0.074^‡^ |
| 24 weeks | 3.11±0.95^b^ | 3.42±0.66^b^ | 0.021 | **Group x Time** | 0.136^‡^ |
| 36 weeks | 3.00±1.06^b^ | 3.41±0.67^b^ | 0.009 |  |  |
| 48 weeks | 3.01±1.03^b^ | 3.39±0.65^c^ | 0.018 |  |  |
| **HBsAg reduction (log_10_IU/mL)** |  |  |  |  |  |
| baseline | 0.00±0.00 | 0.00±0.00 | 1.000 | **Group** | 0.033^‡^ |
| 12 weeks | 0.09±0.30^a^ | 0.05±0.35^a^ | 0.103 | **Time** | 0.073^‡^ |
| 24 weeks | 0.39±0.73^b^ | 0.07±0.46^b^ | <.001 | **Group x Time** | 0.033^‡^ |
| 36 weeks | 0.50±0.88^b^ | 0.08±0.46^b^ | <.001 |  |  |
| 48 weeks | 0.48±0.87^b^ | 0.11±0.45^c^ | <.001 |  |  |
| **HBeAg Seroconversion** |  |  |  |  |  |
| 12 weeks | 4(5.3%)^a^ | 0(0.0%)^a^ | 0.120 | **Group** | 0.015^†^ |
| 24 weeks | 10(13.3%)^ab^ | 2(2.7%)^a^ | 0.017 | **Time** | <0.001^†^ |
| 36 weeks | 12(16.0%)^b^ | 3(4.1%)^a^ | 0.015 | **Group x Time** | 0.139^†^ |
| 48 weeks | 15(20.0%)^b^ | 5(6.8%)^a^ | 0.018 |  |  |
| **HBeAg loss** |  |  |  |  |  |
| 12 weeks | 9(12.0%)^a^ | 6(8.1%)^a^ | 0.430 | **Group** | 0.283^†^ |
| 24 weeks | 17(22.7%)^b^ | 11(14.9%)^a^ | 0.223 | **Time** | <0.001^†^ |
| 36 weeks | 19(25.3%)^bc^ | 10(13.5%)^a^ | 0.068 | **Group x Time** | 0.624^†^ |
| 48 weeks | 24(32.0%)^c^ | 20(27.0%)^b^ | 0.506 |  |  |

^*^ Data are presented as mean±SD or number (%), and Bonferroni’s post-hoc test was used for multiple comparisons between each the five time points. Means with different superscript letters are significantly different (P < 0.05).

^†^ P values were from a generalized estimating equation.

^‡^ P values were from a generalized linear mixed model.

NA, nucleos(t)ide analogues; PegIFNα-2a, peginterferon α-2a; HBsAg, hepatitis B surface antigen; HBeAg, hepatitis B e antigen.
