# Supplementary material for: Effect of switching from nucleos(t)ide maintenance therapy to PegIFN alfa-2a in patients with HBeAg-positive chronic hepatitis B: A randomized trial
Source: PLoS One. 2022 Jul 22;17(7):e0270716. doi: 10.1371/journal.pone.0270716 (PMC9307167; doi:10.1371/journal.pone.0270716)
Supplement: S2 Table — (DOCX) [file pone.0270716.s003.docx]

**S2 Table. Outcomes at each assessment time in the two groups based on ITT with LOCF, ITT without OCF, and complete case analysis.**

|  | **ITT analysis with LOCF** | | | | | **ITT analysis without LOCF** | | | | | **Complete case analysis** | | | | |
| --- | --- | --- | --- | --- | --- | --- | --- | --- | --- | --- | --- | --- | --- | --- | --- |
|  | **Group** | |  | **Analysis for repeated measures** | | **Group** | |  | **Analysis for repeated measures** | | **Group** | |  | **Analysis for repeated measures** | |
| **Variable** | **PegIFNα-2a**  **(n=75)** | **NA**  **(n=74)** | **p** | **Source** | **p** | **PegIFNα-2a**  **(n=75)** | **NA**  **(n=74)** | **p** | **Source** | **p** | **PegIFNα-2a**  **(n=75)** | **NA**  **(n=74)** | **p** | **Source** | **p** |
| **HBsAg (log_10_IU/mL)** |  |  |  |  |  |  |  |  |  |  |  |  |  |  |  |
| Baseline | 3.50±0.55^a^* | 3.49±0.51^a^ | 0.962 | **Group** | 0.515^‡^ | 3.50±0.55^a^ | 3.49±0.51^a^ | 0.962 | **Group** | 0.326^‡^ | 3.44±0.52^a^ | 3.49±0.48^a^ | 0.603 | **Group** | 0.233^‡^ |
| 12 weeks | 3.41±0.70^a^ | 3.44±0.53^a^ | 0.938 | **Time** | 0.074^‡^ | 3.39±0.70^a^ | 3.42±0.51^a^ | 0.980 | **Time** | <0.001^‡^ | 3.38±0.57^a^ | 3.46±0.51^a^ | 0.374 | **Time** | <0.001^‡^ |
| 24 weeks | 3.11±0.95^b^ | 3.42±0.66^b^ | 0.021 | **Group x Time** | 0.136^‡^ | 3.20±0.80^b^ | 3.41±0.68^a^ | 0.053 | **Group x Time** | 0.021^‡^ | 3.20±0.72^b^ | 3.48±0.51^a^ | 0.015 | **Group x Time** | 0.001^‡^ |
| 36 weeks | 3.00±1.06^b^ | 3.41±0.67^b^ | 0.009 |  |  | 3.09±0.87^b^ | 3.48±0.51^a^ | 0.008 |  |  | 3.11±0.81^b^ | 3.47±0.52^a^ | 0.008 |  |  |
| 48 weeks | 3.01±1.03^b^ | 3.39±0.65^c^ | 0.018 |  |  | 3.14±0.77^b^ | 3.44±0.50^a^ | 0.018 |  |  | 3.12±0.76^b^ | 3.44±0.50^a^ | 0.016 |  |  |
| **HBsAg reduction (log_10_IU/mL)** |  |  |  |  |  |  |  |  |  |  |  |  |  |  |  |
| Baseline | 0.00±0.00 | 0.00±0.00 | 1.000 | **Group** | 0.033^‡^ | 0.00±0.00 | 0.00±0.00 | 1.000 | **Group** | 0.0495^‡^ | 0.00±0.00 | 0.00±0.00 | 1.000 | **Group** | 0.005^‡^ |
| 12 weeks | 0.09±0.30^a^ | 0.05±0.35^a^ | 0.103 | **Time** | 0.073^‡^ | 0.09±0.30^a^ | 0.06±0.36^a^ | 0.127 | **Time** | <0.001^‡^ | 0.06±0.16^a^ | 0.03±0.32^a^ | 0.082 | **Time** | <0.001^‡^ |
| 24 weeks | 0.39±0.73^b^ | 0.07±0.46^b^ | <.001 | **Group x Time** | 0.033^‡^ | 0.31±0.59^b^ | 0.05±0.45^a^ | <.001 | **Group x Time** | 0.048^‡^ | 0.24±0.35^b^ | 0.00±0.24^a^ | <.001 | **Group x Time** | 0.002^‡^ |
| 36 weeks | 0.50±0.88^b^ | 0.08±0.46^b^ | <.001 |  |  | 0.38±0.57^b^ | 0.01±0.23^a^ | <.001 |  |  | 0.33±0.50^b^ | 0.01±0.24^a^ | <.001 |  |  |
| 48 weeks | 0.48±0.87^b^ | 0.11±0.45^c^ | <.001 |  |  | 0.31±0.48^b^ | 0.05±0.22^a^ | <.001 |  |  | 0.32±0.49^b^ | 0.05±0.22^a^ | <.001 |  |  |
| **HBeAg seroconversion** |  |  |  |  |  |  |  |  |  |  |  |  |  |  |  |
| 12 weeks | 4(5.3%)^a^ | 0(0.0%)^a^ | 0.120 | **Group** | 0.015^†^ | 4(5.5%)^a^ | 0(0.0%)^a^ | 0.148 | **Group** | 0.074^†^ | 4(6.3%)^a^ | 0(0.0%)^a^ | .1484 | **Group** | 0.176^†^ |
| 24 weeks | 10(13.3%)^ab^ | 2(2.7%)^a^ | 0.017 | **Time** | <0.001^†^ | 10(13.9%)^ab^ | 3(4.3%)^a^ | 0.047 | **Time** | <0.001^†^ | 9(14.3%)^ab^ | 1(2.4%)^a^ | .0484 | **Time** | 0.079^†^ |
| 36 weeks | 12(16.0%)^b^ | 3(4.1%)^a^ | 0.015 | **Group x Time** | 0.139^†^ | 12(18.2%)^b^ | 1(2.3%)_a_ | 0.011 | **Group x Time** | 0.577^†^ | 11(17.5%)^b^ | 1(2.4%)^a^ | .0254 | **Group x Time** | 0.917^†^ |
| 48 weeks | 15(20.0%)^b^ | 5(6.8%)^a^ | 0.018 |  |  | 14(21.2%)^b^ | 5(7.2%)^b^ | 0.020 |  |  | 13(20.6%)^b^ | 1(2.4%)^a^ | .0073 |  |  |
| **HBeAg loss** |  |  |  |  |  |  |  |  |  |  |  |  |  |  |  |
| 12 weeks | 9(12.0%)^a^ | 6(8.1%)^a^ | 0.430 | **Group** | 0.283^†^ | 9(12.3%)^a^ | 6(12.2%)^a^ | 0.989 | **Group** | 0.762^†^ | 9(14.3%)^a^ | 4(10.8%)^a^ | .7634 | **Group** | 0.642^†^ |
| 24 weeks | 17(22.7%)^b^ | 11(14.9%)^a^ | 0.223 | **Time** | <0.001^†^ | 17(23.9%)^b^ | 11(16.4%)^a^ | 0.272 | **Time** | <0.001^†^ | 15(23.8%)^ab^ | 5(13.5%)^a^ | .2143 | **Time** | <0.001^†^ |
| 36 weeks | 19(25.3%)^bc^ | 10(13.5%)^a^ | 0.068 | **Group x Time** | 0.624^†^ | 18(27.3%)^bc^ | 6(13.6%)^a^ | 0.090 | **Group x Time** | 0.621^†^ | 17(27.0%)^b^ | 5(13.5%)^a^ | .1163 | **Group x Time** | 0.365^†^ |
| 48 weeks | 24(32.0%)^c^ | 20(27.0%)^b^ | 0.506 |  |  | 23(34.8%)^c^ | 18(27.7%)^b^ | 0.377 |  |  | 21(33.3%)^b^ | 7(18.9%)^a^ | .1213 |  |  |

^*^ Data are presented as mean±SD or number (%), and Bonferroni’s post-hoc test was used for multiple comparisons between each the five time points. Means with different superscript letters are significantly different (P < 0.05).

^†^ P values were derived by a generalized estimating equation.

^‡^ P values were derived by a generalized linear mixed model.

ITT, intention to treat; LOCF, last observation carried forward; NA, nucleos(t)ide analogues; PegIFNα-2a, peginterferon α-2a; HBsAg, hepatitis B surface antigen; HBeAg, hepatitis B e antigen
