# Supplementary material for: Effect of switching from nucleos(t)ide maintenance therapy to PegIFN alfa-2a in patients with HBeAg-positive chronic hepatitis B: A randomized trial
Source: PLoS One. 2022 Jul 22;17(7):e0270716. doi: 10.1371/journal.pone.0270716 (PMC9307167; doi:10.1371/journal.pone.0270716)
Supplement: S3 Table — (DOCX) [file pone.0270716.s004.docx]

**S3 Table. Changes in serum HBV DNA levels in the two groups.**

| **Variable** | **PegIFNα-2a**  **(n=75)** | **NA**  **(n=74)** | **p** |
| --- | --- | --- | --- |
| **HBV DNA, log_10_ IU/mL** |  |  |  |
| baseline | 0.06±0.32 | 0.09±0.37 | 0.709 |
| 12 weeks | 0.45±0.90 | 0.20±0.97 | 0.009 |
| 24 weeks | 2.02±2.14 | 0.25±1.11 | <0.001 |
| 36 weeks | 2.42±2.27 | 0.26±1.10 | <0.001 |
| 48 weeks | 3.14±2.46 | 0.15±0.64 | <0.001 |

Data are presented as mean±SD.

NA, nucleos(t)ide analogues; PegIFNα-2a, peginterferon α-2a
