# Supplementary material for: Effect of switching from nucleos(t)ide maintenance therapy to PegIFN alfa-2a in patients with HBeAg-positive chronic hepatitis B: A randomized trial
Source: PLoS One. 2022 Jul 22;17(7):e0270716. doi: 10.1371/journal.pone.0270716 (PMC9307167; doi:10.1371/journal.pone.0270716)
Supplement: S4 Table — (DOCX) [file pone.0270716.s005.docx]

**S4 Table. Predictors of a decline in HBsAg by 0.5 log_10_ IU/mL or more at week-48 in all 149 patients.**

| **Characteristic** | **HBsAg decline^*^** | | **Univariate**  **p-value†** | **Multivariate**  **p-value†** |
| --- | --- | --- | --- | --- |
|  | **>0.5log_10_(IU/mL) (n=20)** | **≤0.5log_10_(IU/mL)**  **(n=129)** |  |  |
| Male | 17 (85%) | 96 (74.4%) | 0.304 |  |
| Age, years | 44.85 (7.68%) | 46.48 (11.07%) | 0.524 |  |
| Liver cirrhosis | 2 (10%) | 15 (11.6%) | 0.831 |  |
| Baseline HBsAg, log_10_ IU/mL | 3.41 (±0.77) | 3.51 (±0.48) | 0.436 |  |
| HBsAg at week-24, log_10_ IU/mL | 2.07 (±1.38) | 3.44 (±0.51) | <0.001 | 0.909 |
| HBsAg reduction at week-24, log_10_ IU/mL | 1.34 (±1.16) | 0.06 (±0.19) | <0.001 | 0.004 |
| Baseline HBV DNA by PCR, log_10_ IU/mL | 0.15 (±0.46) | 0.06 (±0.32) | 0.314 |  |
| Baseline ALT, U/L | 34.00 (±38.80) | 25.87 (±16.11) | 0.137 |  |
| ALT elevation associated with PegIFNα-2a | 12 (60%) | 35 (27.1%) | 0.003 | 0.615 |
| PegIFNα-2a/NA | 18/2(90%/10%) | 57/72(44.2%/55.8%) | 0.002 | 0.289 |
| **Previous antiviral agent** |  |  |  |  |
| Entecavir | 10 (50%) | 56 (43.4%) | 0.162 |  |
| Lamivudine | 0 (0%) | 9 (7%) |  |  |
| Tenofovir | 0 (0%) | 13 (10.1%) |  |  |
| Lamivudine + adefovir | 6 (30%) | 14 (10.9%) |  |  |
| Entecavir + adefovir | 1 (5%) | 15 (11.6%) |  |  |
| Entecavir + tenofovir | 2 (10%) | 12 (9.3%) |  |  |
| Lamivudine + tenofovir | 1 (5%) | 10 (7.8%) |  |  |

Data are presented as mean±SD or number (%),

† Logistic regression was performed for comparison

HBsAg, hepatitis B surface antigen; NA, nucleos(t)ide analogues; ALT, alanine transaminase; PegIFNα-2a, peginterferon α-2a.
