# Supplementary material for: Effect of switching from nucleos(t)ide maintenance therapy to PegIFN alfa-2a in patients with HBeAg-positive chronic hepatitis B: A randomized trial
Source: PLoS One. 2022 Jul 22;17(7):e0270716. doi: 10.1371/journal.pone.0270716 (PMC9307167; doi:10.1371/journal.pone.0270716)
Supplement: S5 Table — (DOCX) [file pone.0270716.s006.docx]

**S5 Table. HBsAg levels in the 75 patients who received PegIFN alfa-2a alone or PegIFN alfa-2a with NA retreatment.**

| Variable | NA retreatment | | Non-NA retreatment | | p^*^ |
| --- | --- | --- | --- | --- | --- |
|  | **n** | **Mean±SD** | **n** | **Mean±SD** |  |
| HBsAg (log_10_IU/mL) |  |  |  |  |  |
| 12 weeks | 1 | 3.70±0.00 | 74 | 3.41±0.71 | - |
| 24 weeks | 8 | 3.29±1.40 | 67 | 3.09±0.89 | 0.572 |
| 36 weeks | 11 | 3.25±1.18 | 64 | 2.96±1.04 | 0.407 |
| 48 weeks | 35 | 3.09±0.86 | 40 | 2.95±1.16 | 0.557 |

* All p values were from an independent samples t-test.

NA, nucleos(t)ide analogues; PegIFNα-2a, peginterferon α-2a; HBsAg, hepatitis B surface antigen
