# Supplementary material for: Effect of switching from nucleos(t)ide maintenance therapy to PegIFN alfa-2a in patients with HBeAg-positive chronic hepatitis B: A randomized trial
Source: PLoS One. 2022 Jul 22;17(7):e0270716. doi: 10.1371/journal.pone.0270716 (PMC9307167; doi:10.1371/journal.pone.0270716)
Supplement: S6 Table — (DOCX) [file pone.0270716.s007.docx]

**S6 Table. Achievement of two different combined end-points by patients in the two groups.**

| Combined end-point | Treatment group | | p |
| --- | --- | --- | --- |
|  | **PegIFNα-2a**  **(n=75)** | **NA**  **(n=74)** |  |
| HBeAg loss + HBV DNA < 2000 IU/mL | | | |
| 12 weeks | 7 (9.3%) | 6 (8.1%) | 0.791^*^ |
| 24 weeks | 14 (18.7%) | 11 (14.9%) | 0.535* |
| 36 weeks | 14(18.7%) | 10(13.5%) | 0.392* |
| 48 weeks | 18(24.0%) | 20(27.0%) | 0.672* |
| HBeAg Seroconversion + HBsAg < 100 IU/mL | | | |
| 12 weeks | 0 (0.0%) | 0 (0.0%) | - |
| 24 weeks | 2 (2.7%) | 0 (0.0%) | 0.497** |
| 36 weeks | 2 (2.7%) | 0 (0.0%) | 0.497^**^ |
| 48 weeks | 1 (1.3%) | 0 (0.0%) | 1.000^**^ |

Data are presented as number (%)

* P values were derived from chi-square test.

** P values were derived from Fisher’s exact test.

NA, nucleos(t)ide analogues; PegIFNα-2a, peginterferon α-2a; HBsAg, hepatitis B surface antigen; HBeAg, hepatitis B e antigen.
