# Supplementary material for: Effect of switching from nucleos(t)ide maintenance therapy to PegIFN alfa-2a in patients with HBeAg-positive chronic hepatitis B: A randomized trial
Source: PLoS One. 2022 Jul 22;17(7):e0270716. doi: 10.1371/journal.pone.0270716 (PMC9307167; doi:10.1371/journal.pone.0270716)
Supplement: S7 Table — (DOCX) [file pone.0270716.s008.docx]

**S7 Table. Prognostic factors associated with HBeAg seroconversion at week 48 in all 149 patients.**

| **Variable** | **Overall study population (n = 149)** | | | |
| --- | --- | --- | --- | --- |
|  | **Univariate analysis** | | **Multivariate analysis** | |
|  | **OR (95% CI)** | **p*** | **OR (95% CI)** | **p*** |
| **Age, years** | 0.996(0.953,1.041) | 0.870 |  |  |
| **Gender (male *vs.* female)** | 0.949(0.319,2.822) | 0.925 |  |  |
| **Liver cirrhosis** | 1.450(0.377,5.575) | 0.589 |  |  |
| **Duration of previous NA treatment (months)** | 0.995(0.982,1.009) | 0.470 |  |  |
| **Baseline HBsAg, log_10_ IU/mL** | 0.317(0.126,0.797) | 0.015 | 0.370(0.121,1.134) | 0.082 |
| **HBsAg reduction at week-24, log_10_ IU/mL** | 0.873(0.377,2.021) | 0.752 |  |  |
| **HBsAg reduction at week-48, log_10_ IU/mL** | 0.927(0.461,1.867) | 0.832 |  |  |
| **Baseline HBV DNA by PCR, log_10_ IU/mL** | 0.000(0.000,0.000) | 0.999 |  |  |
| **Baseline ALT, U/L** | 1.021(1.001,1.042) | 0.040 | 1.020(0.988,1.052) | 0.226 |
| **ALT elevation after PegIFNα-2a** | 1.538(0.583,4.060) | 0.384 |  |  |
| **Previous antiviral agent** |  |  |  |  |
| Entecavir | 1.025(0.822,1.279) | 0.825 |  |  |
| Lamivudine |  |  |  |  |
| Tenofovir |  |  |  |  |
| Lamivudine + adefovir |  |  |  |  |
| Entecavir + adefovir |  |  |  |  |
| Entecavir + tenofovir |  |  |  |  |
| Lamivudine + tenofovir |  |  |  |  |
| **PegIFNα-2a/NA** | 3.450(1.184,10.054) | 0.023 | 4.038(1.170,13.934) | 0.027 |

*Logistic regression was performed for comparison

OR: odds ratio; CI: confidence interval; NA, nucleos(t)ide analogues; ALT, alanine transaminase; PegIFNα-2a, peginterferon α-2a; HBsAg, hepatitis B surface antigen; HBeAg, hepatitis B e antigen.
