# Supplementary material for: Effect of switching from nucleos(t)ide maintenance therapy to PegIFN alfa-2a in patients with HBeAg-positive chronic hepatitis B: A randomized trial
Source: PLoS One. 2022 Jul 22;17(7):e0270716. doi: 10.1371/journal.pone.0270716 (PMC9307167; doi:10.1371/journal.pone.0270716)
Supplement: S8 Table — (DOCX) [file pone.0270716.s009.docx]

**S8 Table. Achievement of two different end-points by patients in the two groups.**

|  | **Group** | |  |
| --- | --- | --- | --- |
| **Variable** | **PegIFNα-2a**  **(n=75)** | **NA**  **(n=74)** | **p** |
| **HBsAg<100** **IU/mL** |  |  |  |
| at 48 weeks | 8 (10.7%) | 2 (2.7%) | .098^2^ |
| **HBsAg reduction>0.5 log_10_IU/mL per year** |  |  |  |
| at 48 weeks | 20 (26.7%) | 2 (2.7%) | <.001^1^ |

Categorical variables are presented as numbers with percentages. (%)

^1^ P value from the chi-square test.

^2^ P value from Fisher’s exact test.

PegIFNα-2a, peginterferon α-2a; NA, nucleos(t)ide analogues; HBsAg, hepatitis B surface antigen.
