# Supplementary figures and images for: Effect of switching from nucleos(t)ide maintenance therapy to PegIFN alfa-2a in patients with HBeAg-positive chronic hepatitis B: A randomized trial
Source: PLoS One. 2022 Jul 22;17(7):e0270716. doi: 10.1371/journal.pone.0270716 (PMC9307167; doi:10.1371/journal.pone.0270716)

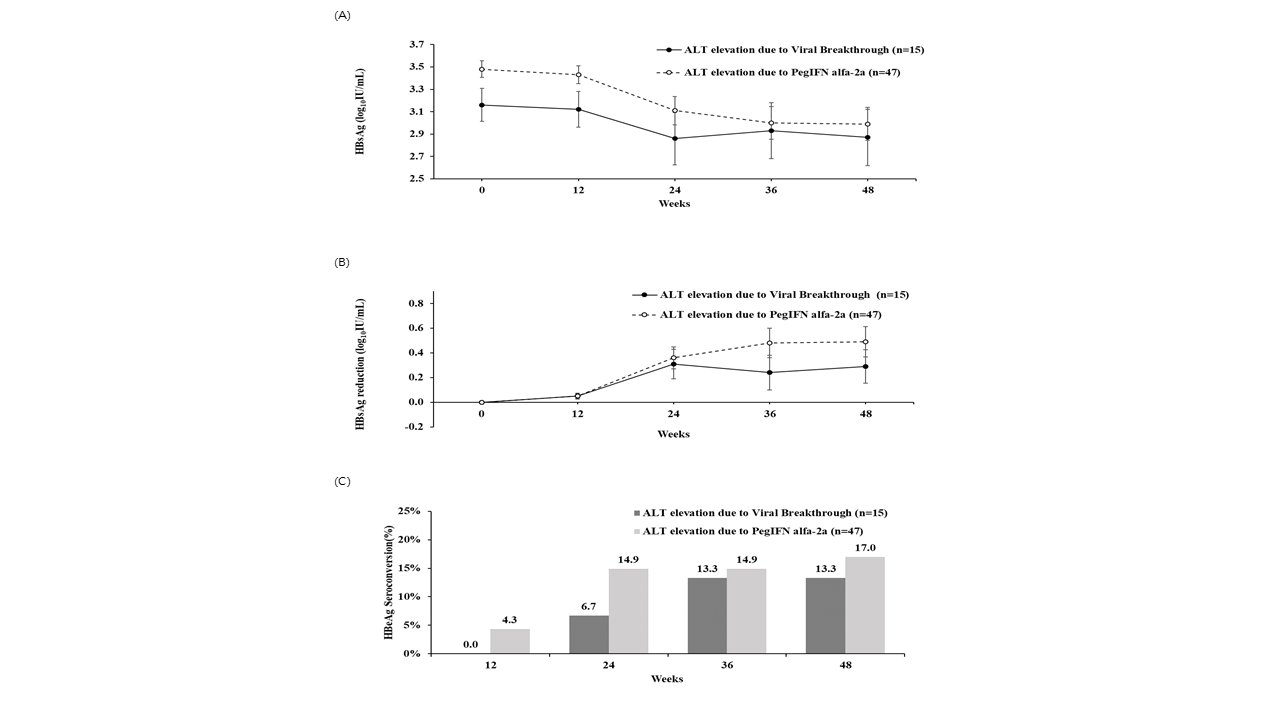

Supplement: S1 Fig — Changes of HBsAg level (A), HBsAg reduction (B), and HBeAg seroconversion (C) in patients with ALT elevation due to viral breakthrough or due to use of PegIFN alfa-2a. HBsAg, Hepatis B surface Antigen; ALT, alanine aminotransferase; HBeAg, hepatitis B e Antigen; PegIFNα-2a, peginterferon α-2a. (TIF) [file pone.0270716.s010.tif]

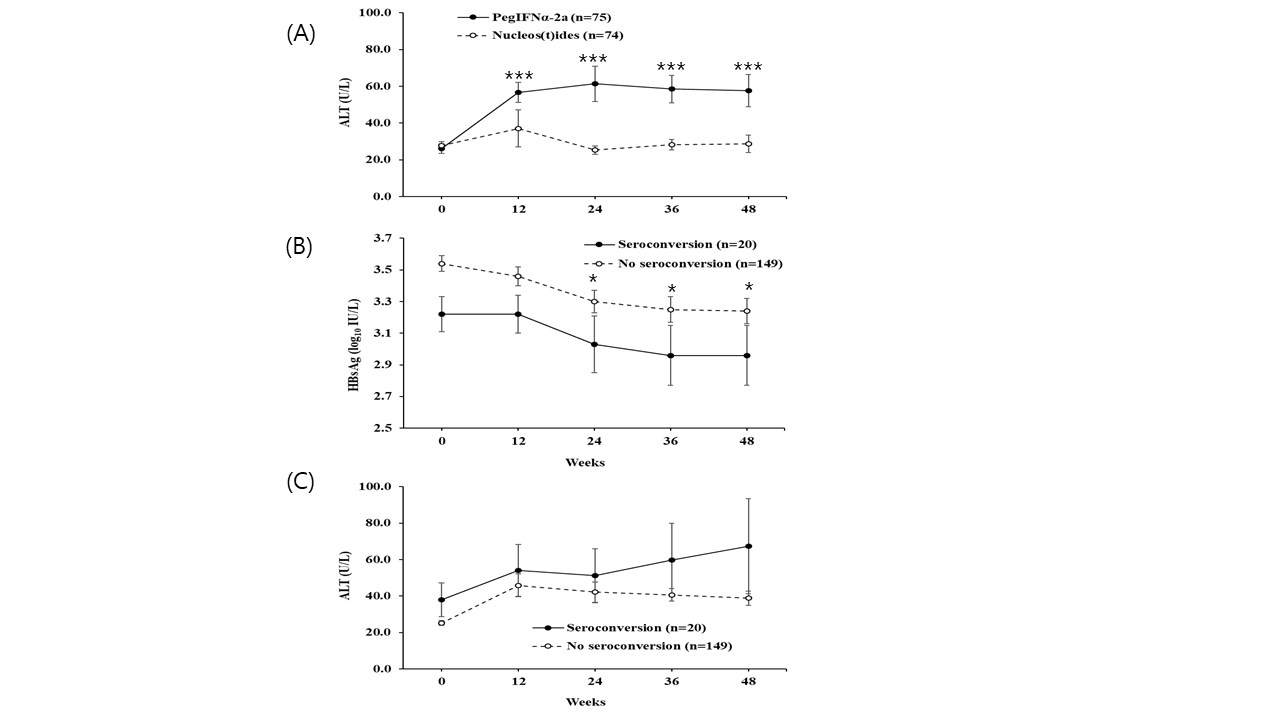

Supplement: S2 Fig — Change in ALT level (A) during treatment for the PegIFN and NA groups and changes in HBsAg level (B) and ALT level (C) according to HBeAg seroconversion at week 48. HBsAg, Hepatis B surface Antigen; ALT, alanine aminotransferase; NA, nucleos(t)ide analogues; PegIFNα-2a, peginterferon α-2a. * p < .05, *** p < .001. (TIF) [file pone.0270716.s011.tif]
