# Supplementary material for: Effect of switching from nucleos(t)ide maintenance therapy to PegIFN alfa-2a in patients with HBeAg-positive chronic hepatitis B: A randomized trial
Source: PLoS One. 2022 Jul 22;17(7):e0270716. doi: 10.1371/journal.pone.0270716 (PMC9307167; doi:10.1371/journal.pone.0270716)
Supplement: S1 File — (PDF) [file pone.0270716.s013.pdf]

IRB 승인 신청 자료

장기간 뉴클레오타이드 유지요법 중인 e항원 양성 만성 B형간염환자에서 페그인터페론 48주 투여에 따른 표면항원 정량 변화양상과 e항원 혈청전환 관련성에 대한 연구

A study to evaluate the dynamics changes of HBsAg quantity and its relation with HBeAg seroconversion following 48 weeks pegylated-interferon-alpha treatment in patients with HBeAg positive chronic hepatitis B after long term nucleos(t)ide analogue maintenance therapy

부산대학교병원 소화기내과

허　　정

목 차

1. 임상연구 심의의뢰서

2. 임상연구 계획서 요약

3. 임상연구 계획서

4. 피험자 설명문 및 동의서

5. 증례기록서 - 별첨

6. 주연구자 이력서

7. 임상연구 계획서 영문 요약

1. 임상연구 심의의뢰서

제출기관 : ▣ 부산대학교병원
심의종류 : ▣ 초심의 □ 보완 심의

임상연구 심의 의뢰서

수신 : 부산대학교 병원장

참조 : 임상연구심의위원회 위원장

연구주최	▣ 연구자 주도 □ 의뢰자 주도			
과제명	국 문	장기간 뉴클레오타이드 제재 유지요법 중인 e항원 양성 만성 B형간염환자에서 페그인터페론 48주 투여에 따른 표면항원 정량 변화양상과 e항원 혈청전환 관련성에 대한 연구		
	영 문	A study to evaluate the dynamics changes of HBsAg quantity and its relation with HBeAg seroconversion following 48 weeks pegylated-interferon-alpha treatment in patients with HBeAg positive chronic hepatitis B after long term nucleos(t)ide analogue maintenance therapy		
	Protocol No.		Version No.	Version 0.9

시험자		소속	직위	성명	연락처	이메일
	시험책임자	부산대학교 병원 소화기내과	부교수	허 정	240-7869	jheo@pusan.ac.kr
	시험담당자	부산대학교 병원 소화기내과	조교수	우 현 영	240-XXXX	who54@hanmail.net

연구내용			
	▣ 임상시험 (▣ 약물, □ 건강기능식품, □ 의료기기, □ 의료행위(시술, 수술, 진단방법) □ 유전자 치료연구, □ 배아줄기세포 연구 □ 세포치료연구 □ 기타)<br>□ 관찰연구 (□ 임상정보 □ 임상시료 □ 설문조사)□ 기타		
	연구약제	일반명:<br>Peginterferon α2a<br>Lamivudine<br>Entecavir<br>Adefovir	상품명:<br>Pegasys®<br>Zeffix®<br>Baraclude®<br>Hepsera®
	임상시험	▣ 학술용 □ 국내 허가용(KFDA) □ 해외 허가용 (국가명 :) □ 해당없음	
		Phase	□I □II(a , b) ▣III □IV □생물학적 동등성 □기타( )

다기관 공동연구			
	□ 국제 ▣ 국내		
	참가국가수 1 개국	참가기관수 1 개 센터	국내기관 3 개 센터
	□ 전체 P.I (국가명: 센터명: 책임자명 :)		
	▣ 국내 P.I (기관명: 부산대학교병원 책임자명: 허 정)		

연구대상수	전체: 144 명(국내: 144 명/국외 명) ⁝ 본 기관 배정: 70 명
피험자 동의취득	▣ 필요 □ 불필요 (사유서 제출)
연구예정기간	시작일자 ⁝ 2012 년 2 월 1 일 ⁝ 종료예상일자 ⁝ 2020 년 12 월 31 일
연구비	현금 ⁝ 총 원(간접비 포함) ⁝ 현물 ⁝
연구비 지원기관	□ 정부기관 ( ) ▣ 기업체 (한국로슈)<br>□ 기타 ( ) □ 개인 ( ) □ 없음
연구의뢰자	회사명 : ⁝ 사업자 등록번호 :<br>대표(직위) : ⁝ 성명 : (인)
연구의 필요성 및 개요	만성 B형간염 환자에서 HBV DNA와 HBeAg은 바이러스의 증식, 활동성 간염의 지표로서 혈청 HBV DNA가 높고 HBeAg 양성인 B형간염은 간경변증이나 간세포암종의 위험을 증가시킨다. B형간염의 자연경과에서 HBeAg의 혈청소실이나 전환이 이뤄진 환자는 간경변증이나 간세포암종 발생 빈도가 낮아 장기예후가 좋은 것으로 알려져 있다. HBV DNA의 감소가 더 중요한 치료의 목표로 제시되고 있다. HBV DNA가 증가되어 있는 활동성 간염 환자에서 치료를 통해 HBV DNA 수치를 감소시키면 조직 소견 호전, HBeAg의 혈청전환, ALT가 정상화되며 간염의 진행을 억제할 수 있다.<br>경구용 항바이러스제를 사용하여 바이러스의 증식이 억제된 후에 언제까지 치료를 지속해야 하는지에 대해서는 아직 정해진 것이 없다. HBeAg 음성 간염은 치료 종결 후 대부분 재발을 하며, HBeAg 양성 간염에서는 혈청전환 후에도 치료를 지속한 경우 바이러스 증식 억제 지속률이 높아지나 HBeAg 혈청전환률은 1년에 20% 내외에 불과하고 상당수의 환자는 HBV DNA가 상업적인 HBV DNA PCR감도 이하로 낮게 측정되어도 수 년간 계속 HBeAg 양성을 유지하며, 이런 경우 경구용 항바이러스제를 투여 중지하면 대부분 치료이전의 상태로 되돌아간다.<br>최근 치료평가의 대체지표로 HBsAg에 대한 연구가 많이 이뤄지고 있다. B형간염의 자연경과에서 HBsAg의 혈청소실률은 연간 1~2%정도 일어나고, HBV DNA 감소와 관계되어 간경변증, 간세포암종 등 간 관련 합병증의 가능성이 감소한다. 인터페론 알파 치료를 통해 바이러스 증식이 억제된 환자에서 HBsAg혈청소실률이 높았고 HBsAg 혈청소실이 일어난 환자에서 간기능소실, 간세포암종 등과 같은 간 관련 사망률의 감소를 보였지만, HBsAg의 소실률은 매우 낮은 편이다.<br>HBsAg의 혈청소실률은 경구용 항바이러스 치료 군보다 인터페론 치료 군에서더 높지만, 경구용 항바이러스제 사용 기간이 길어지면 HBsAg의 소실률도 인터페론 치료만큼 증가될 수도 있을 가능성은 있다. 페그인터페론 치료 후 HBsAg 역가의 감소와 HBV DNA 감소 사이에 상관관계를 보이고 있어, HBsAg 혈청소실 뿐만이 아니라 혈청 내 역가에 대한 연구가 활발히 이뤄지고 있어 향후 B형간염 치료의 좋은 지표로 이용될 것으로 기대된다.<br>장기간 경구용항바이러스제재를 투여받아 HBV DNA가 PCR감도 이하로 낮게 측정되는 HBeAg 양성 간염 환자에서 장기간 페그인터페론 알파 치료를 통해 HBsAg정량변화와 HBeAg의 혈청전환과의 상관성을 규명하는 연구가 필요할 것이다.

피험자 정보	연구 대상군 : ▣ 환자 □ 건강인 □ 취약한 피험자 군 (아래에 체크하시오)<br>□ 임산부 □ 영/소아 □ 외국인 □ 학교의 학생<br>□ 연구기관, 연구자, 의뢰자 등의 피고용인 □ 시험책임자의 연구원이나 학생<br>□ 수감자 □ 시설에 수용된 자 □ 사회적 낙인이 찍힌 질환을 가진 자<br>□ 군인 또는 군대 조직에 의한 피험자 모집<br>□ 장애인 (□ 정신적, □ 육체적, □ 인지적)
특성	피험자 모집 문건(광고, 전단, 인터넷, 이메일 등)의 사용:<br>□ 예 ▣ 아니오<br>(사용할 경우 반드시 IRB 심의를 받고 승인을 득한 뒤 사용- 본원 양식 참조)<br>시술/투약/검사 등의 중재여부: ▣ 예 / □ 아니오<br>피험자의 사적 정보의 이용 여부: ▣ 예 / □ 아니오<br>시료의 수집 및 보관 여부: ▣ 예 / □ 아니오<br>Sample 해외 유출: □ 예 / ▣ 아니오<br>유전학적 정보의 수집 및 보관 여부: ▣ 예 (유전자동의서 제출) / □ 아니오<br>경제적 이해관계 여부: □ 예 / ▣ 아니오<br>사용되는 시술: □ 침습적 / ▣ 비침습적 / □ 해당 사항 없음
제출자료 목록 (Version No. 첨부자료 제출)	▣ 계획서 요약 / ▣ 연구계획서<br>▣ 피험자 설명문 및 동의서 / □ 피험자 동의서 면제 사유서<br>□ 유전자 동의서 제출 / ▣ 증례기록서<br>▣ 연구비 내역서 / ▣ 연구 책임자 최근 3년 이력 및 경력에 관한 문서<br>□ 피험자 모집 공고안 / □ 피험자 보상 규약
피험자 비용 부담	▣ 통상진료 □ 시험약 □ 대조약 ▣ 진료비 ▣ 검사비 □ 기타<br>▣ 통상진료외 □ 시험약 □ 대조약 ▣ 진료비 ▣ 검사비 □ 기타
중간보고서 제출	▣ 있음 □ 없음 (사유서 제출)
연락처 - 연구원	회사명 / 부서명: 부산대학교병원 소화기내과 / 성명: 우 현 영 (인)<br>Tel:240-7869 / HP: 010-9067-0411 / FAX: / E-mail: who54@hanmail.net
연락처 - 코디네이터	성명 (인)<br>Tel: / HP : / FAX: / E-mail:

위와 같이 임상연구심의를 의뢰합니다. 본인은 제출한 연구계획서에 의거하여 성실하게 연구를 수행하겠습니다.

신청일자: 2016 년 10 월 일

시험책임자: 허 정 (인)

※ 접수처 기재사항

IRB No.		접수일		접수처 확인	(인)

2. 임상연구 계획서 요약

◈ 계획서 요약

<table>
<tr><td>과제명</td><td colspan="3">장기간 뉴클레오타이드 제재 유지요법 중인 e항원 양성 만성 B형간염환자에서 페그인터페론 48주 투여에 따른 표면항원 정량 변화양상과 e항원 혈청전환 관련성에 대한 연구</td></tr>
<tr><td>시험책임자</td><td colspan="3">부산대학교 소화기내과 부교수 허 정</td></tr>
<tr><td>시험담당자</td><td colspan="3">부산대학교 소화기내과 조교수 우현영</td></tr>
<tr><td>실시기관</td><td colspan="3">부산대학교병원</td></tr>
<tr><td>담당 관리약사</td><td colspan="3"></td></tr>
<tr><td>공동연구기관</td><td colspan="3">양산부산대학교병원</td></tr>
<tr><td>목적</td><td colspan="3">장기간 뉴클레오타이드 제재 유지요법 중인 e항원 양성 만성 B형간염환자를 대상으로 페그인터페론 48주 투여를 통한 표면항원 정량 변화양상과 e항원 혈청전환 관련성 연구하고자 한다.</td></tr>
<tr><td>시험설계 개요</td><td colspan="3">본 연구는 전향적, 무작위 배정, 공개형, 다기관 임상 연구로서 장기간 뉴클레오타이드 제재 유지요법으로 낮은 HBV DNA치를 유지하고 있지만 e항원 양성인 만성 B형간염환자를 대상으로 기존 뉴클레오타이드 유지요법군과 페그인터페론 투여군 사이에 표면항원 정량 변화양상과 e항원 혈청전환률을 비교하는 것이다.</td></tr>
<tr><td>연구약</td><td colspan="3">페가시스 (Pegasys)
1) 약품명/상품명 : Peginterferon α2a /Pegasys®
2) 원료 약품/성분의 분량 : 1 프리필드 시린지(0.5 mL) 중 주성분 180 ug 함유
3) 제형 : 프리필드 시린지
4) 제약회사/제조사 : 한국로슈</td></tr>
<tr><td>대조약</td><td colspan="3">라미부딘 (Lamivudine)
1) 약품명/상품명 : Lamivudine/Zeffix®
2) 원료 약품/성분의 분량 : 1 정 중 100 mg 함유
3) 제형 : 필름코팅정
4) 제약회사/제조사 : 글락소 스미스클라인
엔테카비어 (Entecavir)
1) 약품명/상품명 : Entecavir/Baraclude®
2) 원료 약품/성분의 분량 : 1 정 중 0.5 mg 함유
3) 제형 : 필름코팅정
4) 제약회사/제조사 : 한국비엠에스제약
아데포비어 (Adefovir)
1) 약품명/상품명 : Adefovir/Hepsera®
2) 원료 약품/성분의 분량 : 1 정 중 10 mg 함유
3) 제형 : 필름코팅정
4) 제약회사/제조사 : 글락소 스미스클라인</td></tr>
<tr><td>대상질환명</td><td>대상성 간기능을 가진 장기간 뉴클레오타이드 제재 유지요법 중인 e항원 양성 만성 B형 간염 환자 중 저바이러스혈증 (HBV DNA ≤ 400 copies/mL)인 환자</td><td>상병명</td><td>만성 B형 간염</td></tr>
</table>

피험자수	전체 144 명(국내: 144 명, 국외: 명) | 본 기관 배정: 70 명<br>산출근거*<br>기존 문헌정보로부터 두 군간의 예상되는 차이를 0.6, 표준편차를 1.1 로 가정하고, 여기에 5%의 유의수준과 90%의 검정력 하에서<br>필요한 피험자 수는 한 군당 72 명이 필요하다.<br>여기에 10%의 탈락율을 고려시 한 군당 80 명 총 160 명을 모집할 예정이다.<br>Reference) Hou et al., Efficacy and safety Peginterferon Alfa-2a versus Adefovir Dipivoxil(ADV) in treating Lamivudine Resistant HBeAg-Positive CHB, AASLD 2008
선정기준	1. 18세 이상의 남성 또는 여성<br>2. HBsAg이 양성인 만성 B형 간염 환자<br>3. 텔비부딘을 제외한 라미부딘, 아데포비어, 엔테카비어, 테노포비어 단독 또는 병용 투여를 최소 18개월 이상 받은 환자 중 HBV DNA undetectable(400 copies/ml 이하)이 12개월 이상 지속된 환자<br>4. HBeAg 양성 만성 B형간염환자<br>5. ALT 수치가 정상 상한치의 10배 이하인 경우<br>6. 기준 HBsAg 정량 100 IU/mL 이상<br>7. 시험약 첫 투여 전 24시간 이내에 확인된 뇨 또는 혈청 임신검사 결과가 음성(가임기 여성의 경우). 추가적으로 가임기의 배우자가 있는 남성 환자와 여성 환자들은 시험 기간 동안과 치료 완료 후 3개월 동안 확실한 피임법을 사용하여야 한다.<br>8. 본 연구계획서에 따라 치료와 추적관찰이 가능하며 본인 혹은 보호자로부터 서면동의를 받은 자
제외기준	1. 비대상성 간경변증 환자(Childs B-C): 자발성 세균성 복막염, 정맥류에 의한 출혈, 간성 뇌증 또는 간기능 소실의 다른 징후를 보인 병력이 있는 경우<br>2. 간세포암을 시사할 수 있는 임상적, 방사선학적 증거가 있는 경우 (스크리닝 α-fetoprotein 수치 > 50ng/ml, 이형성 결절이 존재하는 경우 (CT, MRI 상 결절의 크기가 1cm 보다 큰 경우이거나 복부초음파 소견상 1.5cm 보다 큰 결절 소견이 있는 경우))<br>3. HCV 또는 HIV의 동반 감염<br>4. 간질환의 다른 원인이 존재하는 경우<br>5. 임신했거나 수유 중인 여성 환자<br>6. 시험 등록 전 6개월 이내에 면역조절제/면역억제제를 투여 받은 경력이 있는 경우<br>7. 간질환 외에 시험에 영향을 줄 수 있을 것으로 판단되는 심각한 질환이 있는 경우 (예를 들어 울혈성 심부전, 신부전, 만성 췌장염, 조절되지 않는 당뇨병, 알코올 중독, 악성종양 등)<br>8. 간이식을 하였거나 간이식을 계획하고 있는 경우<br>9. 인터페론에 대하여 과민성의 병력이 있는 경우<br>10. 현재 투여 중인 뉴클레오타이드 유사체에 대한 저항성이 있는 경우<br>11. 이전에 텔비부딘을 사용한 환자

스크리닝	장기간 뉴클레오타이드 제재 유지요법 중인 e 항원 양성 만성 B 형간염환자에서 혈청 HBV DNA 가 400 copies/mL 이하인 환자를 대상으로 선정기준과 제외기준을 적용하여 스크리닝을 실시한다. 피험자의 적합성 screening 과 baseline 검사결과를 파악하기 위해 치료 전 검사를 실시할 수 있으며 유의한 이상이 있는 대상자는 제외한다.
연구방법	장기간 뉴클레오타이드 제재 유지요법 중인 e항원 양성 만성 B형간염환자 중 혈청 HBV DNA가 400 copies/mL 이하인 환자를 대상으로 스크리닝 시행 → 기존 항바이러스제 투여군 또는 페가시스 투여군으로 일대일 무작위 배정 → 48주간 약물 투여 → 치료 중/종료 후 평가
유효성 평가 항목 및 방법 *	일차 결과변수(primary endpoint)<br>각 군의 항바이러스 제재 투여 중 HBsAg 정량(log_{10} HBsAg) 변화<br><br>이차 결과변수(secondary endpoint)<br>1. 각 군의 항바이러스 제재 투여, 경과관찰 중 혈청 HBV DNA 수치의 베이스라인으로부터의 변화 및 HBV DNA 불검출률과 HBV DNA < 20 IU/mL를 비교<br>① 각 군의 항바이러스 제재 투여, 경과관찰 중 HBV DNA < 2,000 IU/mL 비율비교<br>② 각 군의 항바이러스 제재 투여, 경과관찰 중 HBV DNA < 20,000 IU/mL 비율비교<br>2. 각 군의 항바이러스 제재 투여, 경과관찰 중 e항원 혈청전환률과 소실률 비교<br>3. 각 군의 항바이러스 제재 투여/종료 후 1, 2년째 s항원 혈청전환률과 소실률 비교<br>4. 각 군의 항바이러스 제재 투여/종료 후 HBsAg 정량(log_{10} HBsAg) 변화
통계분석방법	일차 결과변수는 e항원 만성 B형 간염 환자에서 혈청전환을 예측하는 지표이다. 본 임상연구는 시험약(Peginterferon α2a 180ug/mL)의 유효율이 대조약(Lamivudine 100mg, Entecavir 0.5 mg, Adefovir 10 mg)의 유효율에 비해 우등하다는 것을 평가하기 위한 우등성 임상시험(superiority clinical trial)이다. 각각의 치료 효과에 의한 log_{10} HBsAg 은 independent T test 을 이용하여 비교한다. e항원 혈청전환률과 소실률, s항원 혈청전환률과 소실률 등 이차 결과변수에 대한 분석은 로지스틱 회귀분석(logistic regression)을 이용하여 분석한다. 본 임상시험에 대한 유효성 및 안정성 분석은 Intent-to-treat (ITT)분석을 원칙으로 한다.

임상연구 계획서

1. 연구 제목

국문: 장기간 뉴클레오타이드 제재 유지요법 중인 e항원 양성 만성 B형간염환자에서 페그인터페론 48주 투여에 따른 표면항원 정량 변화양상과 e항원 혈청전환 관련성에 대한 연구

영문: A study to evaluate the dynamics changes of HBsAg quantity and its relation with HBeAg seroconversion following 48 weeks pegylated-interferon-alpha treatment in patients with HBeAg positive chronic hepatitis B after long term nucleos(t)ide analogue maintenance therapy

2. 연구 배경 및 근거

1) 연구 배경

전 세계적으로 약 3억 명이 B형간염 바이러스(HBV)에 감염되어 있으며 우리나라는 20세 이상 남자의 5.8%-10.9%, 여자의 1.5%-4.4%가 감염되어 있다. 이 중 25%는 만성 B형 간염과 관련한 심각한 합병증을 앓는다.

1998년 투약이 간편하고 부작용이 적은 경구용 항바이러스제로 lamivudine이 소개된 이후 여러 뉴클레오사이드 및 뉴클레오타이드 구조 유도체들이 새롭게 개발되고 있으며 이들은 HBV 복제를 억제하고 ALT를 정상화시키며 조직학적 소견을 개선시킨다고 알려져 있다. 이러한 항바이러스제 치료의 목표는 단기적으로 HBV DNA를 억제하고 혈청 알라닌 아미노전이효소(alanine aminotransferase, ALT)치를 정상화하고, 간조직의 염증과 괴사를 감소시키는데 있다. 장기적인 목표로서는 간경변증과 말기 간질환으로의 진행, 간암의 예방에 있다.

만성 B형간염 환자에서HBV DNA와 HBeAg은 바이러스의 증식, 활동성 간염의 지표로서 혈청 HBV DNA가 높고 HBeAg 양성인 B형간염은 간경변증이나 간세포암종의 위험을 증가시킨다. B형간염의 자연경과에서 HBeAg의 혈청소실이나 전환이 이뤄진 환자는 간경변증이나 간세포암종 발생 빈도가 낮아 장기예후가 좋은 것으로 알려져 있다. HBV DNA의 감소가 더 중요한 치료의 목표로 제시되고 있다. HBV DNA가 증가되어 있는 활동성 간염 환자에서 치료를 통해 HBV DNA 수치를 감소시키면 조직 소견 호전, HBeAg의 혈청전환, ALT가 정상화되며 간염의 진행을 억제할 수 있다.

경구용 항바이러스제를 사용하여 바이러스의 증식이 억제된 후에 언제까지 치료를 지속해야 하는지에 대해서는 아직 정해진 것이 없다. HBeAg 음성 간염은 치료 종결 후 대부분 재발을 하며, HBeAg 양성 간염에서는 혈청전환 후에도 치료를 지속한 경우 바이러스 증식 억제 지속률이 높아지나 HBeAg 혈청전환률은 1년에 20% 내외에 불과하고 상당수의 환자는 HBV DNA가 상업적인 HBV DNA PCR감도 이하로 낮게 측정되어도 수 년간 계속 HBeAg 양성을 유지하며, 이런 경우 경구용 항바이러스제를 투여 중지하면 대부분 치료이전의 상태로 되돌아 간다.

최근 치료평가의 대체지표로 HBsAg에 대한 연구가 많이 이뤄지고 있다. B형간염의 자연경과에서 HBsAg의 혈청소실률은 연간 1~2%정도 일어나고, HBV DNA 감소와 관계되어 간경변증, 간세포암종 등 간 관련 합병증의 가능성이 감소한다. 인터페론 알파 치료를 통해 바이러스 증식이 억제된 환자에서 HBsAg혈청소실률이 높았고 HBsAg 혈청소실이 일어난 환자에서 간기능소실, 간세포암종 등과 같은 간 관련 사망률의 감소를 보였지만, HBsAg의 소실률은 매우 낮은 편이다.

HBsAg의 혈청소실률은 경구용 항바이러스 치료 군보다 인터페론 치료 군에서 더 높지만, 경구용 항바이러스제 사용 기간이 길어지면HBsAg의 소실률도 인터페론 치료만큼 증가될 수도 있을 가능성은 있다. 페그인터페론 치료 후 HBsAg 역가의 감소와 HBV DNA 감소 사이에 상관관계를 보이고 있어, HBsAg 혈청소실 뿐만

이 아니라 혈청 내 역가에 대한 연구가 활발히 이뤄지고 있어 향후 B형간염 치료의 좋은 지표로 이용될 것으로 기대된다.

본 임상 연구는 장기간 경구용항바이러스제재를 투여받아 HBV DNA가 PCR감도 이하로 낮게 측정되는 HBeAg 양성 간염 환자에서 장기간 페그인터페론 알파 치료를 통해 HBsAg정량변화와 HBeAg의 혈청전환과의 상관성을 규명하고자 한다.

2) 임상 시험의 근거

경구용 항바이러스제를 사용하여 e항원 양성 B형간염환자에서 바이러스의 증식이 억제되더라도 HBeAg 혈청전환률은 1년에 20% 내외에 불과하고, e항원 양성이면 지속적으로 경구용 항바이러스제를 투여해야한다. 과거 단기간의 경구용 항바이러스제와 페그인터페론 α2a 병용/순차요법으로 경구용 항바이러스제 단독요법에 비해 우월한 효용성을 보이지 못하였으나, 1년 이상 장기간 항바이러스제 투여받은 e항원 음성 B형간염 환자에서 페그인터페론 α2a 투여 후 표면항원 감소가 뚜렷하였던 대상자에서 치료종료 후 바이러스 활성 억제가 유지되었다.

본 임상 시험에서는 장기간 뉴클레오타이드 유지요법 중인 e항원 양성 만성 B형간염환자를 대상으로 페그인터페론 48주 투여에 따른 표면항원 정량 변화양상과 e항원 혈청전환 관련성을 통해 임상적 이점을 연구하고 분석하고자 한다.

3. 연구 목적

1) 일차 목적 :
각 군의 항바이러스 제재 투여 중 HBsAg 정량(log10 HBsAg) 변화

2) 이차 목적

① 각 군의 항바이러스 제재 투여, 경과관찰 중 혈청 HBV DNA 수치의 베이스라인으로부터의 변화 및 HBV DNA 불검출률과 HBV DNA < 20 IU/mL를 비교
  ㉮ 각 군의 항바이러스 제재 투여, 경과관찰 중 HBV DNA < 2,000 IU/mL 비율비교
  ㉯ 각 군의 항바이러스 제재 투여, 경과관찰 중 HBV DNA < 20,000 IU/mL 비율비교

② 각 군의 항바이러스 제재 투여, 경과관찰 중 e항원 혈청전환률과 소실률 비교
③ 각 군의 항바이러스 제재 투여/종료 후 1, 2년째 s항원 혈청전환률과 소실률 비교
④ 각 군의 항바이러스 제재 투여/종료 후 HBsAg 정량(log10HBsAg) 변화

4. 연구 실시 기관 및 기간

1) 연구 실시 기관명 및 주소
기관: 부산대학교 병원
주소: 부산광역시 서구 구덕로, 602-73

2) 다기관 공동 연구기관 및 책임연구자 (가나다순)
경북대학교 병원 / 탁원영 교수
계명대학교 동산의료원 / 황재석 교수
부산대학교 병원 / 허 정 교수
영남대학교 의료원 / 이헌주 교수

3) 기간
공개 연구 시작 후 모든 대상자가 투약 48주 후 경과관찰 24개월 도달 시까지
(연구예정기간: IRB 승인일자 - 2020 년 12 월 31일)

5. 대상자의 선정 또는 제외기준

1) 대상 환자군
연구 모집단은 대상성 간기능을 가진 장기간 뉴클레오타이드 제재 유지요법 중인 e항원 양성 만성 B형 간염 환자 중 저바이러스혈증 (HBV DNA > 400 copies/mL)인 환자 인 환자이다. 연구 참여자들은 1:1의 비율로 기존 경구용 항바이러스제재 투여군과 페가시스 투여군으로 배정된다.

2) 선정기준
① 본 연구계획서에 따라 치료와 추적관찰이 가능하며 본인 혹은 보호자로부터 서면동의를 받은 자
② 18세 이상의 남성 또는 여성
③ 텔비부딘을 제외한 라미부딘, 아데포비어, 클레부딘, 엔테카비어, 테노포비어 단독 또는 병용 투여를 최소 18개월 이상 받은 환자 중 HBV DNA undetectable (400 copies/mL 이하)이 12개월 이상 지속된 환자
④ HBsAg이 양성인 만성 B형 간염 환자
⑤ Baseline HBsAg 정량 100 IU/mL 이상
⑥ HBeAg 양성 만성 B형간염환자
⑦ ALT 수치가 정상 상한치의 10배 이하인 경우
⑧ 시험약 첫 투여 전 24시간 이내에 확인된 뇨 또는 혈청 임신검사 결과가 음성 (가임기 여성의 경우). 추가적으로 가임기 배우자가 있는 모든 남성 환자와 임신 가능성이 있는 모든 여성 환자들은 시험 기간 동안과 치료 완료 후 3개월 동안 확실한 피임법을 사용하여야 한다.

3) 제외기준
① 서면동의를 할 수 없는 자
② 비대상성 간경변증 환자(Childs B-C)
: 자발성 세균성 복막염, 정맥류에 의한 출혈, 간성 뇌증 또는 간기능 소실의 다른 징후를 보인 병력이 있는 경우
③ 간세포암을 시사할 수 있는 임상적, 방사선학적 증거가 있는 경우
㉮ 스크리닝 alpha-fetoprotein 수치 > 100 ng/ml,
㉯ 이형성 결절이 존재하는 경우 (CT, MRI 상 결절의 크기가 1cm 보다 큰 경우이거나 복부초음파 소견상 1.5cm 보다 큰 결절 소견이 있는 경우))
④ HAV, HCV, HDV 또는 HIV의 동반 감염
⑤ 간질환의 다른 원인이 존재하는 경우
⑥ 임신했거나 수유 중인 여성 환자
⑦ 시험 등록 전 6개월 이내에 면역조절제/면역억제제를 투여 받은 경력이 있는 경우
⑧ 간질환 외에 시험에 영향을 줄 수 있을 것으로 판단되는 심각한 질환이 있는 경우 (예를 들어 울혈성 심부전, 신부전, 만성 췌장염, 조절되지 않는 당뇨병, 알코올 중독, 악성종양 등)
⑨ 시험 등록 전 1년 이내에 알콜 또는 약물 중독 병력이 있는 경우
⑩ 주요 장기 이식을 하였거나 이식을 계획하고 있는 경우
⑪ 인터페론 투여 금기증이 있는 경우
⑫ 인터페론에 대하여 과민성의 병력이 있는 경우
⑬ 현재 투여 중인 뉴클레오타이드 유사체에 대한 저항성이 있는 경우
⑭ 이전에 텔비부딘을 사용한 환자

6. 목표 대상자의 수 및 산출 근거

1) 목표 대상자 수
   각 치료군당 72명 (총 144명)

2) 산출 근거
   기존 문헌정보로부터 두 군간의 예상되는 차이를 0.6, 표준편차를1.1로 가정하고, 여기에 5%의 유의수준과 90%의 검정력 하에서 필요한 대상자 수는 한 군당 72명이 필요하다. 여기에 10%의 탈락율을 고려시 한 군당 80명 총 160명을 모집할 예정이다.
   Reference) Hou et al., Efficacy and safety Peginterferon Alfa-2a versus Adefovir Dipivoxil(ADV) in treating Lamivudine Resistant HBeAg-Positive CHB, AASLD 2008

7. 연구 설계 및 방법

1) 임상 시험의 개요

   장기간 뉴클레오타이드 제재 유지요법 중인 e항원 양성 만성 B형간염환자 중 혈청 HBV DNA가 400 copies/mL 이하인 환자를 대상으로 스크리닝 시행 → 기존 항바이러스제 투여군 또는 페가시스 투여군으로 일대일 무작위 배정 → 48주간 약물 투여 → 치료 중/종료 후 평가

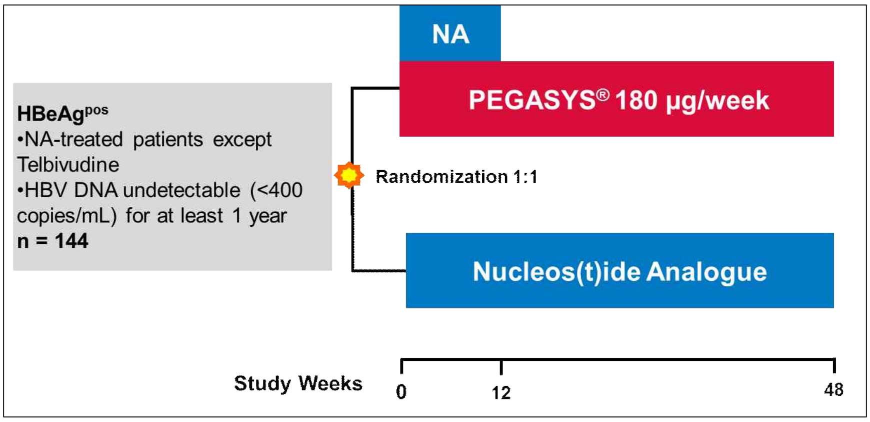

2) 임상 시험의 대상 의약품
   ① 시험약: Peginterferon α2a
      ㉮ 약품명/상품명: Peginterferon α2a /Pegasys®
      ㉯ 원료 약품/성분의 분량
         ⓐ 1 프리필드 시린지 180 (0.5 mL) 중 주성분 180 ug함유
         ⓑ 1 프리필드 시린지 135 (0.5 mL) 중 주성분 135 ug함유
         ⓒ 180 마이크로그램프로클릭 (0.5 mL) 중 주성분 180 ug함유
         ⓓ 135 마이크로그램프로클릭 (0.5 mL) 중 주성분 135 ug함유

      ㉰ 제형
         ⓐ 1 프리필드 시린지 180
         ⓑ 1 프리필드 시린지 135

ⓒ 180 마이크로그램프로클릭
ⓓ 135 마이크로그램프로클릭

㉱ 제약회사/제조사 : 한국로슈

② 대조약: Lamivudine
㉮ 약품명/상품명: Lamivudine/Zeffix®
㉯ 원료 약품/성분의 분량: 1 정 중 100 mg 함유
㉰ 제형: 필름코팅정
㉱ 사용방법: 1일 1회 경구투약
㉲ 제약회사/제조사: 글락소 스미스클라인

③ 대조약: Entecavir
㉮ 약품명/상품명: Entecavir/Baraclude®
㉯ 원료 약품/성분의 분량: 1정 중 0.5 mg 함유
㉰ 제형: 필름코팅정
㉱ 사용방법: 1일 1회 경구투약
㉲ 제약회사/제조사: 한국비엠에스제약, Bristol-Myers Squibb

④ 대조약: Adefovir
㉮ 약품명/상품명: adefovir/Hepsera®
㉯ 원료 약품/성분의 분량: 1정 중 10 mg 함유
㉰ 제형: 필름코팅정
㉱ 사용방법: 1일 1회 경구투약
㉲ 제약회사/제조사: 글락소 스미스클라인

⑤ 대조약: 레보비르
㉮ 약품명/상품명: clevudine/Levovir®
㉯ 원료 약품/성분의 분량: 1정 중 10 mg 함유
㉰ 제형: 경질캡슐
㉱ 사용방법: 1일 1회 경구투약
㉲ 제약회사/제조사: 부광약품

⑥ 대조약: 비리어드
㉮ 약품명/상품명: tenofovir/Viread®
㉯ 원료 약품/성분의 분량: 1정 중 300 mg 함유
㉰ 제형: 필름코팅정
㉱ 사용방법: 1일 1회 경구투약
㉲ 제약회사/제조사: 길리어드 사이언스

3) 대상 의약품의 투여 용량 및 기간
① Peginterferon α2a 투여군: Peginterferon α2a 180 ug/주 48주
② 라미부딘 투여군: Lamivudine 100 mg QD 지속
③ 엔테카비어 투여군: Entecavir 0.5 mg QD 지속
④ 아데포비어 투여군: adefovir 10 mg QD 지속
⑤ 클레부딘 투여군: clevudine 10 mg QD 지속
⑥ 테노포비어 투여군 : tenofovir 300 mg QD 지속

4) 병용 금지 약물
① B형 간염에 대한 항바이러스 효과를 가진 기타 다른 약제들은 허용되지 않는다. 단, 아래의 경우에는 허용한다.

㉮ 약제 투여 중 약제에 대한 내성으로 인해 '임상적 돌파(clinical breakthrough)'가 발생하는 경우
* 임상적 돌파(clinical breakthrough)는 '바이러스 돌파(virologic breakthrough)'와 동반하여 ALT 정상 후 정상 상한치의 2배 이상 상승한 경우로 본 연구에서 'biochemical flare'로 정의함. – 이 경우에는 Adefovir rescue therapy를 시행할 수 있다.
* 치료 기간 중 '바이러스 돌파(virologic breakthrough)'는 발생하였으나, '생화학적 돌파(biochemical breakthrough)'의 정도가 ALT 상승이 정상 상한치의 2배 미만인 경우는 시험자의 판단에 따라 rescue therapy 여부를 결정할 수 있다..

② 면역억제제 (systemic corticosteroids, Mycophenolate moferil, cyclosporine 및 anti-metabolite (azathioprine 등) 복용은 허용되지 않는다.
③ 연구기간 중 Biphenyldimethyldicarboxylate(DDB) 계통의 약물 복용은 허용하지 않으나 기타 간기능을 증진/보호하는 약제는 연구자의 판단으로 일부 허용할 수 있다.

5) 무작위 배정 방법 및 눈가림화 여부
연구 시작 시점에 Peginterferon α2a 투여군과 기존 항바이러스 제재 투여군으로 일대일 무작위 배정 후 공개 연구로 시행한다. 약제를 배정하는 데 있어 층화확률화계획법을 적용한다. 다기관 연구에 참여하는 각 병원별로 따로 마련된 확률화배정표(randomization table)에 따라서 환자들이 할당되도록 한다. 그러나, 대상자 모집이 원활하지 못할 경우 모든 연구자의 동의 하에 전체 모집 예정수가 채워질 때까지 경쟁등록 방식으로 무작위배정을 변경하기로 한다.

8. 관찰 항목

1) 검사
① 치료 전 스크리닝
대상자 동의, 선정/제외 기준 검토, 병력 확인, 활력징후, 신체검사, HBV DNA, serology lab (HBeAg, Anti-HBe), hematology lab (WBC, RBC, Hemoglobin, Platelet count, Differential count), serum chemistry (Calcium, Inorganic P, Glucose, BUN, Creatinine, Uric acid, Cholesterol, Total protein, Albumin, Alkaline phosphatase, AST/ALT, T. bilirubin), PT, α-fetoprotein, Abdomen U/S (or CT, MRI), 기초 안과 검사
(이들 검사 중 Alpha-fetoprotein, 영상학적 검사, HBV DNA, serology lab은 3달 이내의 검사, Hematology lab, Serum chemistry는 1달 이내의 검사는 스크리닝 및 베이스라인 검사로 인정한다.)

② 치료 시작 후 매 12주 마다 (투약 48주까지)
질병의 악화 여부 확인, 이상반응 & 병용약물 확인, Serum chemistry(Calcium, Inorganic P, Glucose, BUN, Creatinine, Uric acid, Cholesterol, Total protein, Albumin, Alkaline phosphatase, AST/ALT, T. bilirubin), HBV DNA, HBsAg 정량

③ 치료 시작 후 매 24주 마다 (투약 48주까지)
α-fetoprotein , Abdomen U/S (or CT, MRI) 시행
④ 치료 시작시 12주 마다 HBeAg/Ab check 하며 seroconversion이 연속 2회에서 확인이 되면 연구자 판단에 의해 중단 가능

⑤ 그 외의 검사 항목은 연구자의 판단 하에 진행한다.

9. 예측 부작용 및 사용상의 주의사항

1) Peginterferon α2a

① 다음 환자에게는 투여하지 말 것

이 약 또는 이 약의 다른 성분에 대한 과민증 환자, 인터페론 알파 제제에 과민한 환자, 자가면역성 간염 환자, 중증의 정신 이상이 있거나 병력이 있는 환자 약물요법으로 조절되지 않는 갑상선 질환이 있는 환자, 중증 간장애, 대상부전 간 질환자, 간 이외의 기관 이식 환자, 통제할 수 없는 발작 및/또는 중추신경계 기능이상 환자, 임산부, 신생아 및 3세 이하의 소아(이 약은 벤질 알코올을 함유하고 있음, 경고항 참조), 중증 심장 질환이 있는 환자, 중증 신장 질환이 있는 환자, 중증 골수기능 장애 환자, 백신 등 생물학적 제제에 과민증 환자, Child-Pugh score 6이상인 간경화증을 동반한 HIV-HCV 중복 감염 환자.

② 다음 환자에게는 신중히 투여할 것

㉮ 심질환 또는 그 병력이 있는 환자 : 직접적인 심장 독성은 증명되지 않았으나 이 약 투여 후 흔히 나타나는 약물자체의 급성 부작용(예:발열, 오한)이 기존 심장 상태를 악화시킬 수 있다.

㉯ 신장, 간 또는 골수기능 장애 환자 : 경미-중등도의 기능장애가 있을 경우 이들 기능을 주의 깊게 관찰해야 한다.

㉰ 알레르기 소인이 있는 환자

㉱ 당뇨병 또는 그 병력, 가족력이 있는 환자, 내당력 장애 환자(당뇨병이 악화 또는 발생하기 쉽다)

㉲ 자가면역질환 또는 그 소인이 있는 환자

㉳ 고혈압환자

㉴ 혈액학적검사 기저치로서 호중구수가 1,500/㎣미만이거나, 혈소판수 75,000/㎣미만 또는 적혈구수가 10g/dl(빈혈)미만인 환자

㉵ 폐질환(예:만성 기도 폐색 질환) 또는 그 병력이 있는 환자

㉶ 건선환자(건선이 악화될 수 있다)

㉷ 정신질환 병력이 있는 환자

③ 부작용

㉮ 일반증상 : 피로, 근육통, 경직, 주사부위 국소반응, 무력증, 통증, 인플루엔자양 증상, 권태, 졸음증, 떨림, 열성 홍조, 허약, 단순포진. 특히 인플루엔자양증상(피로, 발열, 오한, 식욕부진, 두통, 관절통, 근육통, 발한 등)이 나타나며, 이들 증상은 아세트아미노펜에 의해 부분적으로 억제된다. 대개는 감량으로 부작용의 정도가 감소된다.

㉯ 소화기계 : 식욕부진, 구역, 구토, 소화불량, 설사, 복통, 위염, 구강건조, 구강궤양, 잇몸출혈, 드물게 변비, 복부팽만감, 구내염, 설염, 운동항진증, 가슴쓰림, 소화성궤양의 재발 및 생명에 위험성이 없는 위장관 출혈이 몇 예 보고된 바 있다.

㉰ 당뇨병(인슐린 의존형(IDDM) 및 인슐린 비의존형(NIDDM)) : 정기적으로 검사(혈당치, 뇨당 등)하고 이상이 나타난 경우에는 적절한 처치를 한다.

㉱ 간장 : AST, ALT, ALP, LDH 및 빌리루빈치 상승과 같은 간기능 변화가 관찰될 수 있다.

㉲ 정신신경계 : 어지러움, 의식장애, 착란, 두통, 수면장애, 신경과민, 초조감, 불면, 혼수, 졸음, 우울증, 집중력저하, 불안, 기억상실, 감정적장애, 기분변화, 신경질, 공격성, 성욕감퇴, 조루, 자극과민성, light-headedness(현훈없이), 뇨실금, 경련, 치매양 증상(특히 고령자), 환청, 방향감각상실 등이 나타날 수 있다.

㉳ 말초신경계 : 때때로 지각이상, 감각저하, 미각이상, 혼몽, 저림, 신경질환, 진전, CPK의 상승 등이 나타날 수 있다.

㉴ 심혈관계 : 심근증, 때때로 말초성 허혈, 심부전, 협심증, 심전도이상(기외수축, 심실성빈맥, 심방세동, ST저하 등), 심근 장애, ?통, 빈맥, 서맥, 심계항진, 일과성 저혈압 및 고혈압, 사지부종, 안면홍조, 청색증, 부정맥, 심계항진, 두근거림, 드물게 폐부종, 울혈성심부전, 심장성 호흡정지 및 심근경색증이 보고된 바 있다.

㉵ 피부, 점막, 부속기계 : 구순 포진의 재악화, 피부혈관염 및 피부질환, 발진, 습진, 가려움, 경미-중등도의 탈모, 피부건조, 콧물, 비출혈, 홍반, 발톱변색, 건선, 두드러기, 광과민증 반응, 발한 증가, 수면중 발한, 전신성 홍반성 루푸스 등이 나타날 수 있다.

㉶ 신장 및 요로계 : 드물게 급성신부전등의 중등도의 신장해가 나타날 수 있으므로(주로 신질환 및 신독성 약물과 병용치료중인 암환자) 정기적으로 검사를 하는 등 관찰을 충분히 하고 이상이 인정되는 경우에는 투여를 중지하고 적절한 처치를 한다. 단백뇨, 뇨당양성, 배뇨곤란, 뇨량감소, 다뇨, 요침사이상, 방광염, 혈뇨, 드물게 BUN, 혈청 크레아티닌및 뇨산치의 상승이 나타날 수 있다.

㉾ 혈액계: 백혈구 감소, 혈소판 감소, 적혈구 감소, 과립구 감소, 헤모글로빈 및 헤마토크릿치 감소, 빈혈, 림프절병증 등이 나타날 수 있다.

㉿ 자가면역 현상 : 자가면역 현상에 의한 것이라고 생각되는 증상[갑상선 기능이상(갑상선기능 저하증 또는 갑상성기능 항진증), 간염, 용혈성 빈혈, 궤양성대장염의 악화, 관절 류마치스의 악화, 인슐린 의존형 당뇨병(IDDM)의 악화 또는 발생 등]이 나타날 수 있다.

㉠ 호흡기계 : 발열, 해소, 호흡곤란, 흉부 X선 이상을 수반한 폐렴 및 간질성 폐렴, 폐침윤, 상기도감염, 인후통, 비염, 비인두염, 부비강울혈, 폐울혈, 가슴이 답답함, 운동호흡곤란, 때때로 폐장염이 나타날 수 있으므로 이러한 증상이 나타나는 경우쬐는 투여를 중지하고 부신피질호르몬제를 투여하는 등적절한 처치를 한다.

㉡ 췌장 : 드물게 급성 췌장염이 일어날 수 있으므로 관찰을 충분히 하고 복통, 혈청 아밀라제치의 상승 등이 일어날 경우에는 투여를 중지하고 적절한 처치를 한다.

㉢ 뇌출혈 : 뇌출혈(0.1% 미만)이 발생할 수 있으므로 세밀하게 관찰하여 이상이 발견되면 투여를 중지하고 적절한 처치를 한다.

㉮ 근골격계 : 관절통, 근육통, 뼈의 통증, 경부통, 요통, 근육경련, 근육허약

㉯ 눈 : 흐린시력, 안구건조, 눈염증, 안구통증. 드물게 망막출혈, 면모반, 시각신경유두부종 또는 망막 동맥이나 정맥의 폐색 등 안구 이상이 알파-인터페론 치료 후 드물게 보고된다. 시력 감소나 시력 상실을 보고하는 환자들은 안구검사를 해야 한다. 이러한 종류의 징후는 다른 질환 상태와 연관될 수 있기 때문에, 당뇨병이나 고혈압 환자들은 치료를 시작하기 전에 안과검사를 실시할 것이 권장된다.

㉰ 기타 : 때때로 체중감소, 혈청단백질감소, 주사부동통, 혈당상승, 류마티양 관절염, 망막출혈, 허혈성 망막병증 등이 나타날 수있다. 또한 패혈증이 나타날 수 있으므로 환자의 전신상태를 충분히 관찰하고 이상이 인정되는 경우에는 투여를 중지하고 적절한 처치를 한다. 유사약(다른 인터페론 알파 제제)에서 혈소판 감소, 용혈성 빈혈, 신부전을 주증상으로 하는 용혈성 요독증 증후군이 발생했다는 보고가 있다.

㉱ 호중구감소증, 혈소판감소증이 표준 인터페론보다 이 약에서 더 많이 발생하였다. 빈혈로 용량조정이 필요한 경우는 경화증 환자를 포함하여 1% 미만이었다. 약 4% 의 환자에서 일시적으로 절대 호중구수가 치료 중에 500/㎣이하로 떨어졌다. 혈소판 수가 50,000/㎣미만으로 감소한 경우는 5%정도에서 관찰되었고 대부분 경화증이 있는 환자이거나 시험에 참가할 당시 혈소판 수가 75,000/㎣으로 낮은 환자들이었다.

④ 약물 상호작용

㉮ 이 약 180㎍을 주 1회 건강한 대상자에게 4주간 투여한 결과 mephenytoin, dapsone, debrisoquin, 또는 tolbutamide의 약물속도론에 아무런 영향을 끼치지 않았다. 따라서, 이 약은 동종효소인 P450 3A4, 2C9, 2C19나 2D6의 생체내 대사활성에 영향을 주지 않는다.

㉯ 이 약의 병용투여로 theophylline(싸이토크롬 P450 1A2 활성의 표식자)의AUC가 25% 증가함으로써 이 약이 싸이토크롬 P450 1A2의 작용을 중등도로 저해한다는 것이 확인되었다. 이 약과 함께 theophylline을 투여하는 환자들은 theophylline의 혈청 농도를 모니터해야 하고 그에 따라 theophylline의 양을 조정해야 한다. Theophylline과 이 약의 최대 상호작용은 이 약의 투여 4주 후에 나타날 것으로 예측된다.

㉰ 인터페론은 이전에 투여한 약물이나 병용하는 약물의 신경독성, 혈액독성 및 심장독성 효과를 증가시키는 것으로 관찰되어 왔으므로 이 약에서도 유사한 결과가 예측된다.

㉱ 다른 인터페론제제와 마찬가지로, 이 약을 다른 골수억제성 약물과 병용시 주의하여야 한다.

㉲ 이 약과 리바비린의 병용 임상시험에서 약물속도론적 상호작용은 관찰되지 않았다.

㉳ 배합금기에 대한 시험은 실시되지 않았으므로 이 약물을 다른 약물과 혼합해서는 안된다.

㉴ 소시호탕과의 병용 예에서 간질성 폐렴의 발현이 보고되었으므로 병용하지 않는다.

㉵ 예방요법으로 메타돈(평균 95mg, 30~150mg)을 동시에 투여받은 후 이 약 180 μg을 매주 피하주사한 HCV 감염 환자 24명을 대상으로 한 약물동력학 시험에서 평균 메타돈의 약물동력학 변수는 4주간의 이 약 투여 전과 비교했을 때, 투여 후에 10~15% 높았다. 이 시험의 임상적 의의는 잘 밝혀지지 않았으나, 환자에게 메타돈의 이상 반응에 대해 주지시켜야 한다.

㉶ HIV-HCV 중복 감염 환자 : 핵산계 역전사효소 억제제(NRTIs, 라미부딘, 지도부딘, 스타부딘)의 세포 인산화(cellular phosphorylation)에 대한 리바비린의 효과를 검증하기 위한 12주간의 약물동력학 substudy을 실시한 47명의 HIV-HCV 중복 감염 환자에서 약물 상호작용은 나타나지 않았다. 리바비린

의 혈장 노출은 NRTIs의 병용 투여에 의해 영향을 받지 않는 것으로 나타났다.

㉲ 리바비린과 디다노신의 병용투여는 권장되지 않는다. 디다노신 또는 활성 대사체(dideoxyadenosine 5'-triphosphate)의노출은 디다노신과 리바비린 병용 투여시 증가된다. 리바비린 사용으로 인한 말초 신경병증, 췌장염, 증후성 유산혈증과 함께 치명적인 간부전이 보고 되었다.

㉳ 정확한 기전은 밝혀지지 않았으나 치료요법의 일부로 지도부딘을 HIV에 투여 받았을 때 리바비린에 의한 빈혈의 악화가 보고되었다. 빈혈의 위험이 증가될 수 있으므로 리바비린과 지도부딘의 병용투여는 권장되지 않는다. ART 복합 요법에서 지도부딘을 교체하는 것을 고려해보아야 한다. 지도부딘으로 인한 빈혈 병력이 있는 환자에서 특히 주의가 필요하다.

㉴ 임상시험에서 1일 1회 텔비부딘 600mg과 매주 이 약 180 μg의 병용투여는 말초 신경병증의 위험 증가와 관련이 있었다. 이와 관련된 작용기전은 알려지지 않았으나 다른 인터페론에 대해서도 이러한 위험이 배제될 수는 없다. 또한, 텔비부딘과 인터페론 알파(페그인터페론 포함) 병용투여 시의 이점은 현재 확립되지 않았다.

㉵ 아자치오프린 : 리바비린은inosine monophosphate dehydrogenase 저해 작용으로 아자치오프린의 대사를 저해하여 6- methylthionosine monophoasphate(6-MTIMP)를 축적시킬 수 있으며 이는 아자치오프린 투여 환자에서의 골수 독성과 관련이 있었다. 페그인터페론 알파-2a 와 리바비린 및 아자치오프린의 병용은 삼가야 한다. 리바비린과 아자치오프린 병용 투여의 이점이 잠재적 위험을 상회하는 경우, 아자치오프린 병용투여 동안 골수독성의 징후를 확인하기 위한 면밀한 혈액학적 모니터링의 실시가 권장되며, 이러한 징후가 확인된 경우 약물 치료를 중단해야 한다.

⑤ 임부 및 수유부에 대한 투여

㉮ 이 약을 임산부에 사용한 경험은 없다. 이 약을 임신 중에 투여해서는 안된다.

2) Lamivudine

① 다음 환자에게는 투여하지 말 것

㉮ 라미부딘 또는 라미부딘의 다른 성분에 과민증이 있는 환자

② 다음 환자에게는 신중히 투여할 것

㉮ 신기능 장애 환자에는 투여 감량이 추천됨

㉯ 췌장염 병력이 있거나 췌장염에 걸릴 위험도가 큰 소아에게 투여시 주의를 요함

㉰ 심각한 지방간에 의한 간비대와 유산증이 보고된 바 있으니 주의 깊게 관찰할 것

㉱ 임신, 비만, 치료 지연에 의해서 유산증과 간손상의 위험이 커질 수 있으니 유의할 것

③ 부작용

㉮ >10%: 중추신경계(두통, 피로), 위장관계(오심, 설사, 구토, 췌장염), 신경계와 근골격계(말초신경염, 감각이상, 근골격 통증)

㉯ 1~10%: 중추신경계(현기증, 우울증, 발열, 오한, 불면증), 피부(발진), 위장관계(식욕부진, 위복부통증, 흉통, amylase증가), 혈액학적(중성구 감소증), 간(AST, ALT증가), 신경계와 근골격계(근육통, 관절통), 호흡기계(기침)

㉰ <1%: 탈모증, 아나필락시스, 빈혈, 간비대, 고빌리루빈혈증, 고혈당, CPK의 증가, 유산증, 림프절종대, 말초 신경병증, 소양증, 횡문근 융해, 무력감, 혈소판 감소증, 위염

④ 약물 상호작용

㉮ 지도부딘(Zidovudine)과 함께 투여 시 지도부딘의 농도를 39%까지 증가 시킬 수 있음

㉯ 트리메토프림-설파메톡사졸은 이 약의 혈중 농도를 증가시킴

㉰ 잘시타빈(Zalcitabine)과 함께 투여 시 세포내 인산화 과정을 서로 저해하여 효력을 떨어뜨리므로 병용을 피할 것.

⑤ 임부 및 수유부에 대한 투여

㉮ 미국(FDA)분규: C, 호주(ADEC)분류: B3

㉯ 임신 초기 3개월 동안의 투여는 권장되지 않는다

3) Entecavir

① 다음 환자에게는 투여하지 말 것.

㉮ 엔테카비어 또는 엔테카비어의 성분에 과민반응이 있는 환자

② 다음 환자에게는 신중히 투여할 것

㉮ 신부전 환자에서는 용량 조절이 권장된다.

㉯ 싸이클로스포린이나 타크롤리무스를 투여 받고 있는 간이식 수여자에서 본제의 요법을 개시하기 전 및 치료 중에 신기능을 주의하여 평가해야 한다.

③ 부작용

㉮ >10%: 간(ALT 증가)

㉯ 1-10%: 중추 신경계(두통, 피로), 내분비 및 대사계(고지혈증), 소화기계(lipase증가, amylase증가, 설사, 소화불량)

㉰ <1%: 현기증, 저알부민혈증, 불면증, 오심, 졸음, 저혈소판증, 구토

④ 약물 상호작용

㉮ 리바비린(Ribavirin)과 병용 투여 시, 간부전이나 기타 미토콘드리아 독성 징후를 보일 수 있음(췌장염, 유산산증 등)

㉯ Gancyclovir, valgancyclovir: 이상반응, 독성 증가 (예, 혈액장애)

⑤ 임부 및 수유부에 대한 투여

㉮ 임신 중에는 유익성이 태아에 대한 위험성을 상회하는 경우에만 사용해야 한다.

㉯ 본제를 복용하는 경우에는 모유를 수유하지 않도록 해야 한다.

4) Adefovir

① 다음 환자에게는 투여하지 말 것.

㉮ 아데포비어 또는 이 약의 다른 성분에 과민증이 있는 환자

② 다음 환자에게는 신중히 투여할 것

라미부딘 내성 B형 간염의 임상적 근거를 보이는 간이식 전(n=226) 및 후(n=24 만성 B형 간염 환자를 대상으로 각각 평균 치료기간 51주 및 99주로 203주까지 이 약으로 공개 임상시험을 진행하였다. 신 기능장애에 대하여 사이클로스포린과 타크로리무스와의 병용투여, 치료초기 신 기능부족, 고혈압, 당뇨, 장기 이식 등을 포함한 위험요소를 지닌 간이식 전 후 환자들에게서 신기능의 변화가 발생하였다. 따라서, 신기능에서의 이러한 변화들에 대해 이 약이 미치는 영향은 평가하기가 어렵다. Kaplan-Meier estimate에 의해 볼 때, 간이식 전 환자들 가운데 48주까지 12%의 환자에서, 96주까지 28%의 환자에서, 144주까지 30%의 환자에서 초기보다 ≥0.5mg/dL의 혈청 크레아티닌 상승이 관찰되었고, 간이식후 환자들 가운데 48주까지 18%의 환자에서, 96주까지 35%의 환자에서, 144주까지 35%의 환자에서 초기보다 ≥0.5mg/dL의 혈청 크레아티닌 상승이 관찰되었다. 치료초기에 간 이식 전이었던 환자들을 고려하면, 48주까지 혈청 크레아티닌이 최소 0.5mg/dL 증가한 것으로 확인된 환자들의 퍼센트(%)에 대한 Kaplan-Meier estimate가 간이식 전보다 간이식 후에서 더 높았다(52% vs 5%). 최종 방문까지 간이식 전 환자 중 1.3%(226명 가운데 3명), 간이식 후 환자 중 2.5%(241명 가운데 6명)에서 <2.0 mg/dL 의 혈청 인 수치를 보였다. 간이식 전 후 환자들 가운데 4%의 환자가(467명 가운데 19명) 신장 반응으로 인해 이 약의 투여를 중단하였다. 이 약으로 치료 받은 간 이식 전후 환자에서 치료와 관련된 것으로 2% 혹은 그 이상의 빈도로 가장 빈번하게 보고된 이상반응은 다음과 같다.

㉮ 전신 : 무력증, 두통, 발열

㉯ 위장관 : 복통, 구역, 구토, 위창자내공기참, 설사, 간부전, 소화불량

㉰ 대사 및 영양 : ALT 및 AST 상승, 비정상적 간기능, 저인산혈증

㉱ 호흡기 : 기침 증가, 인두염, 부비동염

㉲ 피부 및 피부 부속기관 : 가려움증, 발진

㉳ 비뇨기계 : 크레아티닌상승, 신부전, 신기능 부족

③ 부작용

㉮ >10%: 무력증

㉯ 1-10%: 두통, 복통, 구역, 위, 창자 내 가스 팽만, 설사, 소화불량

㉰ <1%: 근육병증, 골연화증, 근위요세관질환, 판코니 증후군, 저인산혈증, 췌장염

④ 약물 상호작용

㉮ 아데포비어는 신장으로 배설되므로 신기능을 감소시키는 약물 또는 양성 세뇨관 분비에 대해 경쟁적으로 작용하는 약물과 이 약을 병용투여 할 때 아데포비어 또는 병용투여되는 약물의 혈청농도를 증가시킬 수 있다.

㉯ 라미부딘과 트리메토프림/설파메톡사졸, 아세트아미노펜, 이부프로펜, 테노포비어를 제외하고는 신장으로 배설되는 약물이나 신기능에 영향을 미치는 것으로 알려진 약물과 이 약을 병용투여 하였을 때 신기능에 미치는 영향은 연구된 바 없다.

㉰ 이 약은 트리메토프림/설파메톡사졸, 아세트아미노펜, 이부프로펜, 라미부딘의 약물동태를 변화시키지 않는다.

㉱ 신장으로 배설되는 약물 또는 신기능에 영향을 미치는 것으로 알려진 기타 약물과 이 약을 병용투여 할 때 이상반응에 대해 주의 깊게 모니터링 하여야 한다.

㉲ 이부프로펜 800밀리그람을 1일 3회 투여하였을 때 아데포비어의 AUC는 약 23%, Cmax는 약 33%가 증가되었다. 이들 증가는 이 약의 신 클리어런스의 감소보다는 생물학적 이용률이 더 높아진 것에 기인한 것으로 생각되며, 아데포비어 노출 증가의 임상적 유의성은 알려지지 않았다.

㉳ 아데포비어는 일반적으로 CYP450 효소를 저해하지 않지만 아데포비어가 CYP450 효소를 유도할 가능성은 알려져 있지 않다.

㉴ 사이클로스포린 및 타크로리무스 농도에 대한 아데포비어의 영향은 알려져 있지 않다.

㉵ 이 약과 라미부딘 100mg을 병용투여했을때 각 약물의 약물동태 양상을 변화시키지는 않았다.

⑤ 임부 및 수유부에 대한 투여

㉮ 임신 중에는 유익성이 태아에 대한 위험성을 상회하는 경우에만 사용해야 한다.

㉯ 본제를 복용하는 경우에는 모유를 수유하지 않도록 해야 한다.

5) Clevudine

① 다음 환자에는 투여하지 말 것

㉮ 클레부딘 또는 이 약의 다른 성분에 과민증이 있는 환자

㉯ 만18세 미만의 환자

㉰ 신기능 장애 환자

㉱ 이 약은 주로 신장을 통해 배설되므로 신장애환자의 경우 클리어런스 감소로 인하여 클레부딘의 배설반감기가 연장될 수 있으므로 용량 조절이 필요하나, 신기능 장애 환자의 용법용량 조절에 대한 임상적 연구는 이루어지지 않은바, 크레아티닌 클리어런스가 60mL/분 미만인 환자는 투여하지 말 것.

② 다음 환자에는 신중히 투여할 것

㉮ 다른 약물과 병용투여 해야 하는 환자

ⓐ 이 약은 신장으로 주로 배설되어, 신기능을 감소시키거나 능동적인 세뇨관분비에 경쟁적인 약물과의 병용투여는 이들 약물의 혈청 농도를 상승시킬 수 있다. 신장으로 배설되거나 신기능에 영향을 미치는 것으로 알려진 다른 약물과 이 약의 병용투여에 대한 영향이 평가되지 않은바, 이 약을 이들 약물과 병용 투여할 때에는 이상반응에 대해 환자를 면밀히 관찰해야 한다.

ⓑ 약물의 대표적 대사 효소인 CYP450 효소계의 저해여부에 대한 in vitro 시험결과, CYP450 1A2, 2C9, 2C19, 2D6, 3A4에 대하여 이 약은 저해제로 작용하지 않음이 확인되었으나, 기질여부, 유도여부에 대해 연구된 바 없으므로 본 효소에 의하여 영향 받는 약물과 병용 투여시 이상반응에 대해 면밀히 관찰해야 한다.

③ 이상반응

4주간 이 약(n=246) 또는 위약(n=84)을 투여한 만성 B형 간염 환자 330명에 대한 두 개의 치료적 확증

임상시험을 근거로 이상반응을 평가하였다. 이들 임상연구에서 이상반응이나 임상검사치 이상으로 시험을 중단한 환자는 위약 치료군에서 약 2.4%이었고, 이 약을 투여 받은 환자군에서는 없었다.

㉮ 임상적 이상반응

이 약의 치료(24주간 1일1회 클레부딘 30mg 투여) 동안 5% 이상 보고된 이상반응은 감염(감기 또는 상기도 감염), 무력감, 복통, 소화불량, 두통 등이다. 이 약과 관련가능성이 있는 중등도~중증의 임상적 이상반응은 감기 또는 상기도감염, 발진, 졸음, 두통이었고 그 발생율은 클레부딘 투여군 에서는 모두 1% 미만이었다. 아래 표는 치료적 확증 임상시험에서 24주 투약 기간 동안 보고된 중등도~중증(Grade 2~4)의 임상적 이상반응이다.

	클레부딘 30mg	위약
두통	0.8%(2/246)	1.2%(1/84)
감기 또는 상기도감염	0.4%(1/246)	2.4%(2/84)
골절	0.4%(1/246)	1.2%(1/84)
발진	0.4%(1/246)	0%
졸음	0.4%(1/246)	0%
봉소염	0.4%(1/246)	0%
결막염	0.4%(1/246)	0%
자궁내막염	0.4%(1/246)	0%
치은염	0.4%(1/246)	0%
위궤양	0.4%(1/246)	0%

㉯ 임상적 검사치 이상

이 약과 위약을 비교한 두 개의 치료적 확증 임상시험에서 치료 중 ALT, ALT, Creatinin phosphokinase, lipase 가 정상 상한치의 2-5배 이상 증가되었다. 이들 연구에서, 이 약 치료 환자 중, 치료 초기의 ALT 또는 AST 상승은 전반적으로 이 약의 투여를 계속함에 따라 소실되었다. 치료 중에도 주기적인 간기능 검사가 권장된다.

㉰ 간염의 악화

이 약의 치료적 확증 임상시험에서 ALT 또는 AST 수치가 > 20×ULN 또는 > 10×ULN 및 기저값의 10배를 초과할 때 '간염의 악화'라고 정의하였다. 치료기간 동안 및 치료 중단 후 추적기간 동안 클레부딘 투여 군에서는 2-5.5%, 위약군에서는 4.9-13%에서 '간염의 악화'가 나타났다.

④ 부작용 (24주 투여 시)

㉮ >10%: 감염(감기 또는 상기도 감염), 무력감, 간효소치증가

㉯ 1-10%: 복통, 소화불량, 두통, 간염악화

㉰ <1%: 골절, 발진, 졸음, 봉소염, 결막염, 자궁내막염, 치은염, 위궤양

⑤ 일반적 주의

㉮ 이 약의 안전성과 유효성은 이 약 30mg을 1일1회 24주간 투여한 위약대조 치료적 확증 임상시험 결과에 근거한 것이며, 활성약을 대조군으로 하여 비교한 임상시험은 연구된 바 없다.

㉯ 치료적 확증 임상시험 결과, 혈청 HBV DNA의 기준시점 대비감소량, HBV DNA의 검출한계 미만인 대상자의 비율, ALT 정상화 비율 측면에서 위약군에 비해 통계적 유의한 차이를 나타내었으나, HBeAg 양성 환자에 있어 혈청전환 비율은 이 약 투여군에서 위약군에 비해 통계적 유의한 차이가 나타나지 않았으며, 간생검을 통한 조직학적 변화는 평가되지 않았다.

㉰ 만성B형 간염환자에서의 이 약의 최적 치료기간과 치료 및 장기간의 결과(간경변 및 간세포성암) 결과와의 상관관계는 알려져 있지 않으며, 진행성 간경변 환자는 간경변 진행상황에 대해 주의깊게 모니터링 해야 한다.

㉱ 이 약으로의 치료가 성관계나 혈액오염으로 인한 HBV전염 위험성을 감소시킨다고 밝혀진바 없으며, 따라서 적절하게 주의를 해야 한다는 것을 환자에게 주지시켜야 한다.

㉲ 뉴클레오시드 저항성 돌연변이 바이러스에 대한 클레부딘의 치료효과

ⓐ in vitro 효과

단독 돌연변이인 M550V, V519L, M553I, A546V를 함유한 HBV에 대해서는 클레부딘이 효과를 나타내지만 M550I에 대해서는 효과가 없었다. 단독 돌연변이인 L526M을 함유한 바이러스에 대한 클

레부딘의 효과는 일정하지 않았다. 클레부딘은 다중 돌연 변이인 L526M/M550I 또는 L526M/M550V를 함유한 HBV에는 활성이 없었다.

ⓑ 임상적 효과

가) 소수(n=7명)의 뉴클레오시드유사체인 라미부딘으로 치료 받은 경험이 있고 HBV DNA polymerase의 YMDD 변이(M550I/V)에 대한 유전학적 증거가 있는 환자를 대상으로 클레부딘의 항바이러스 활성을 평가하는 임상시험 결과, 기준치 대비 HBV DNA 변화 측면에서 평가할 때 현저한 억제 효과는 나타나지 않았다.

나) 클레부딘의 임상시험 결과, 계획한 투여기간동안 클레부딘 군에서 라미부딘 저항성 변이인 L526M, M550I/V는 발견되지 않았으나, 대상자들의 혈청으로부터 분리한 HBV DNA 염기 서열을 비교분석한 결과 polymerase의 conserved site상의 변화가 일부 보고되었다. 이러한 변화들의 일관성 및 HBV DNA 수치 rebound 와의 관련성은 없었으나 HBV의 클레부딘 저항성 유전형에 관한 연구 결과는 제한적이다.

㉥ C형 및 D형간염에 대한 이 약의 임상적 효과는 알려져 있지 않다.

㉦ 비대상성 간질환 환자 또는 장기이식 환자, HCV, HDV 또는 HIV와 HBV에 동시에 감염된 환자 등에서 이 약의 임상경험은 없다.

㉧ 암 화학요법제를 포함한 면역억제제를 투여받고 있는 환자에게 있어서 이 약의 임상경험은 없다.

㉨ 운전이나 기계조작 능력에 이 약이 미치는 영향은 연구된 바 없으며, 이 약의 약리작용으로부터 이러한 능력의 손상을 예측할 수도 없다. 그럼에도 불구하고, 환자의 운전 또는 기계조작 능력을 고려할 때는 그 환자의 임상적 상태와 이 약의 이상반응 측면을 염두에 두어야 한다.

㉩ 시판 후 부작용 조사에서 이 약을 사용했을 때 근육병증의 사례가 보고되었다. 근육병증은 이 계열의 다른 약물에서도 보고되었다.

클레부딘 투여 환자에서 합병증을 수반하지 않은 근육통이 보고되었다. 원인을 알 수 없는 확산형 근육통, 근육압통, 근력약화를 호소하는 환자에서 근육병증을 고려해야 한다. 근육병증은 크레아티닌 키나제(CK)의 상승 정도와 관계없이 지속적으로 원인을 알 수 없는 근육통 그리고/또는 근력 약화로 정의한다. 클레부딘과 관계가 있는 근육병증 환자 사이에서 CK 수치의 정도나 시간과 관련한 일정한 패턴이 존재하지 않는다. 또한 클레부딘 투여 환자에서 근육병증이 발병하는 소인도 밝혀지지 않았다. 지속적으로 알 수 없는 근육통, 근육압통, 근무력이 나타날 때에는 환자가 반드시 사실을 알리도록 해야 한다. 그리고 근육병증이 진단되었을 경우 클레부딘의 투여를 중지해야 한다.

이 계열의 약물을 투여했을 때 근육병증의 위험이 증가하는 것이 근육병증과 관련된 다른 약물과의 병용투여 때문인지에 대한 인과관계는 알려지지 않았다. 의사는 이 약 투여 시 근육병증과 관련이 있는 다른 약물을 함께 투여할 때 잠재적 유익성과 위험성을 주의 깊게 따져보아야 하며 알 수 없는 근육통, 근육압통, 근무력의 증상이나 징후에 대한 모니터링이 필요하다.

㉪ 클레부딘을 6개월 또는 1년, 또는 1년 6개월 복용한 환자를 대상으로 3개월 또는 6개월간 추적 조사한 시점에서 HBV DNA 수치가 4,700 copies/mL 미만, ALT 수치가 정상, HBeAg 양성 환자의 경우 최종 2시점에서 연속적으로 HBeAg seroconversion을 보인 환자를 대상으로 추가로 2년간 추적 조사 하였다. 이 임상시험에 등록된 환자 중 위의 기준을 만족한 환자 63명(HBeAg 양성 환자 : 26명, HBeAg 음성환자 : 37명) 중 2년간 추적 조사 이후에 HBV DNA 수치가 4,700 copies/mL 미만인 환자는 35%이었고(HBeAg 양성환자 : 46%, HBeAg 음성환자 : 27%), HBV DNA 수치가 141,500 copies/mL 미만인 환자는 73%(HBeAg 양성환자 : 77%, HBeAg 음성환자 : 70%)이었다. 그리고 ALT 수치가 정상인 환자는 75%(HBeAg 양성환자 : 88%, HBeAg 음성환자 : 65%)이었으며 HBeAg seroconversion이 81%에서 유지되었다.

⑥ 상호작용

㉮ 음식물과의 상호작용

공복상태와 고지방식이 후의 본 약물 (클레부딘 600mg)의 약물동태 평가 결과 식사상태에서 Cmax는 감소한 반면 Tmax는 증가하여 흡수율이 감소하는 것으로 나타났으나, AUC 및 CL/F, Cmax에 대한 분산계수를 고려할 때 생물학적 이용률은 일정한 것으로 나타났다.

㉯ 약물과의 상호작용

ⓐ 이 약은 신장으로 주로 배설되기 때문에, 신기능을 감소시키거나 능동적인 세뇨관분비에 경쟁적인 약물과의 병용투여는 이들 약물의 혈청 농도를 상승시킬 수 있다. 신장으로 배설되거나 신기능에 영향을 미치는 것으로 알려진 다른 약물과 이 약의 병용투여에 대한 영향을 평가하지는 않았다.

이 약을 이들 약물과 병용 투여할 때에는 이상반응에 대해 환자를 면밀히 관찰해야 한다.

ⓑ 약물의 대표적 대사 효소인 CYP450 효소계의 저해여부에 대한 in vitro 시험결과, CYP450 1A2, 2C9, 2C19, 2D6, 3A4에 대하여 이 약은 저해제로 작용하지 않음이 확인되었으나, 기질여부, 유도여부에 대해 연구된 바 없으므로 본 효소에 의하여 영향 받는 약물과 병용 투여시 이상반응에 대해 면밀히 관찰해야 한다.

⑦ 임부 및 수유부, 소아 및 노인환자 등에 대한 투여

㉮ 임부

랫드 및 토끼에서의 생식독성시험 결과, 최기형성의 증거나, 암수의 생식능력 또는 태자에 미치는 영향은 없었다. 클레부딘을 경구로 랫드에 하루에 1000mg/kg 까지, 토끼에 하루에 500mg/kg까지 투여한 생식독성시험에서 투약에 기인한 태자의 외형적 기형이나 발달 변이는 나타나지 않았다. 동물에서의 생식독성시험을 제외하고 임신부에 대해 진행된 연구는 없었다. 이 약은 임신 중에는 유익성이 태아에 대한 위험성을 상회하는 경우에만 사용해야 한다. 모체로부터 유아에게 HBV의 전염에 대한 이 약의 영향에 대한 데이터는 없다.

㉯ 수유부 : 이 약이 사람의 유즙으로 분비되는지는 밝혀지지 않았으므로 이 약을 복용하는 경우에는 모유를 수유하지 않도록 해야 한다.

㉰ 소아 : 만 18세 미만의 환자에 대한 이 약의 임상적 경험은 없다.

㉱ 고령자 : 이 약의 임상연구에는 65세 이상 대상자가 참여하지 않아서, 청년 대상자와 다르게 반응하는지 확인할 수 없다. 이 약은 상당량이 신장으로 배설되며 고령 환자의 신기능은 더 감소될 것이기 때문에 용량 선정에 주의를 기울여야 하며 투여기간 동안 신기능을 관찰하는 것이 유용하다.

6) Tenofovir

① 다음 환자에는 투여하지 말 것

㉮ 이 약 또는 이 약에 함유된 다른 성분에 과민증이 있는 환자

㉯ 이 약은 유당을 함유하고 있으므로, 갈락토오스 불내성(galactose intolerance), Lapp 유당분해효소 결핍증(Lapp lactase deficiency) 또는 포도당-갈락토오스 흡수장애(glucose-galactose malabsorption) 등의 유전적인 문제가 있는 환자에게는 투여하면 안된다.

② 임상 시험에서의 이상반응

임상 시험은 광범위하고 다양한 조건에서 시행되므로, 약물의 임상 시험에서 관찰되는 이상반응 비율을 다른 약물의 임상 시험에서 관찰되는 이상반응 비율과 직접적으로 비교할 수 없으며, 실제로 관찰되는 비율에 반영되지 않을 수도 있다.

㉮ 만성 B형 간염 환자의 임상 시험

ⓐ 만성 B형 간염 및 대상성 간 질환을 앓고 있는 성인 환자에 대한 임상 시험

치료와 관련된 이상반응: 만성B형간염 환자에 대한 대조 임상시험(0102 및 0103)에서 48주간의 이중 맹검 기간 동안 이약 치료를 받은 대상자가 더 많이 구역을 경험했다(이 약 9% vs 아데포비어 디피복실 제제 2%). 이 약으로 치료를 받은 환자 중 5% 이상이 보고한 기타 치료 관련 이상반응으로는 복통, 설사, 두통, 현기증, 피로, 코인두염, 요통, 피부 발진이 포함된다.

이 시험에서 최대 144주 동안 이 약으로 지속적인 치료를 받은 환자에게서 내약성 프로파일(이상반응의 특성 또는 심각도)에서의 유의한 변화는 관찰되지 않았다.

ⓑ 만성 B형 간염 및 비대상성 간 질환을 앓고 있는 성인 대상자에 대한 임상 시험

소규모의 무작위 이중 맹검, 활성 대조 시험(0108)에서 CHB와 비대상성 간 질환을 앓고 있는 환자를 최대 48주 동안 이 약 또는 기타 항바이러스 약물로 치료했다. 이 약 치료를 받은 45명의 환자가 가장 자주 보고했던 치료 관련 이상반응은 복통(22%), 오심(20%), 불면증(18%), 가려움증(16%), 구토(13%), 현기증(13%), 발열(11%)이었다. 45명 환자 중 2명(4%)은 48주간의 시험 기간 중 간 질환이 진행됨에 따라 사망했다. 45명의 환자 중 3명(7%)은 이상반응으로 인해 치료를 중단했다. 45명의 환자 중 4명(9%)은 혈청 크레아틴이 0.5 mg/dL 증가되었다(또한 1명은 48주 동안 혈청 인산이 2mg/dL 미만으로 되었다). 이러한 환자 중 3명(초기에 Child-Pugh 지수가 10이하이고, MELD 지수가 14이상이었음)은 신장 손상이 악화되었다. 이 약와 비대상성 간 질환이 모두 신장 기능에 영향을 줄 수 있으므로, 이 모집단에서 이 약이 신장 손상에 미친 영향이 어느 정도인지 알아내기는 어렵다.

45명의 대상자 중 한 명은 48주의 시험기간 중 치료 시 간염 발적을 겪었다.

㉯ 부작용 (48주 투여 시)

ⓐ >10%: 무력증, 통증, 설사, 구역

ⓑ 1-10%: 두통, 복통, 요통, 흉통, 발열, 구토, 식욕부진, 소화불량, 복부팽만, 폐렴, 우울증, 불면증, 말초신경병즐, 어지러움, 발진반응, 발한, 근육통, 체중감소

㉰ 시판 후 조사

테노포비어가 승인된 후 사용하는 동안 다음과 같은 이상반응이 확인되었다. 시판 후 이상반응은 불명확한 규모의 모집단에서 자발적으로 보고되기 때문에 항상 확실하게 발생율을 예측하거나 약물 투여에 대한 인과 관계를 입증하지 않는다.

ⓐ 면역계 장애 : 알레르기 반응(혈관부종 포함)

ⓑ 대사 및 영양 장애 : 유산증, 저칼륨증, 저인산혈증

ⓒ 호흡계, 흉부 및 종격 장애 : 호흡곤란

ⓓ 위장 장애 : 췌장염, 아밀라아제 증가, 복통

ⓔ 간담낭 장애 : 간지방증, 간염, 간 효소 증가(가장 일반적으로 AST 및 ALT 감마 GT)

ⓕ 피부 및 피하 조직 장애 : 발진

ⓖ 근골격계 및 결합 조직 장애 : 횡문근 융해증, 골연화증(골통으로 나타나며, 골절을 유발할 수 있음), 근육 약화, 근질환

ⓗ 신장 및 비뇨기 장애 : 급성 신부전, 신부전, 급성 세뇨관 괴사, 판코니 증후군, 근위 세뇨관증, 간질성 신염(급성인 경우 포함), 신장성 요붕증, 신부전, 크레아티닌 증가, 단백뇨, 다뇨증

ⓘ 일반 장애 및 투약 부위 병태 : 무력증

ⓙ 상기 나열된 이상반응에서 횡문근 융해증, 골연화증, 저칼륨증, 근육 약화, 근질환, 저인산혈증은 근위 신장 세뇨관증의 결과로 발생할 수 있다.

㉱ 신장 기능에 영향을 주는 약물

테노포비어는 원칙적으로 신장에서 배설되기 때문에 신장기능을 감소시키거나 능동적 세뇨관 분비에 대해 경쟁하는 약물과 이 약을 함께 투약하면 테노포비어의 혈청 농도를 증가시키거나 신장에서 제거되는 다른 약물의 농도를 높일 수 있다. 반드시 이에 한정되지는 않지만 몇 가지 예로는 시도포비어, 아시클로비어, 간시크로비어, 발라사이클로비어 및 발간시클로비어 등이 있다. 신기능을 약화시키는 약물도 테노포비어의 혈청 농도를 높일 수 있다.

10. 임상연구 중지 또는 탈락 기준

1) 중도 탈락 기준

① 대상자가 본 시험 참여에 대한 동의를 철회한 경우

② 심각한 부작용이 발생한 경우

2) 시험 종료 기준

① 무작위배정 후 Peginterferon α2a 를 48주간 투약 종료 후

11. 유효성 평가기준, 평가방법 및 해석 방법(통계분석 방법)

1) 유효성 평가 기준

① 주 결과변수(primary endpoint): 각 군의 항바이러스 제재 투여 중 HBsAg 정량(log10 HBsAg) 변화

② 보조 결과변수(secondary endpoint)

㉮ 각 군의 항바이러스 제재 투여, 경과관찰 중 혈청 HBV DNA 수치의 베이스라인으로부터의 변화 및

HBV DNA 불검출률과 HBV DNA < 20 IU/mL를 비교

ⓐ 각 군의 항바이러스 제재 투여, 경과관찰 중 HBV DNA < 2,000 IU/mL 비율비교

ⓑ 각 군의 항바이러스 제재 투여, 경과관찰 중 HBV DNA < 20,000 IU/mL 비율비교

㉯ 각 군의 항바이러스 제재 투여, 경과관찰 중 e항원 혈청전환률과 소실률 비교

㉰ 각 군의 항바이러스 제재 투여/종료 후 1, 2년째 s항원 혈청전환률과 소실률 비교

㉱ 각 군의 항바이러스 제재 투여/종료 후 HBsAg 정량(log10HBsAg) 변화

2) 통계 분석 방법

일차 결과변수는 e항원 만성 B형 간염 환자에서 혈청전환을 예측하는 지표이다. 본 임상연구는 시험약(Peginterferon α2a 180ug/mL)의 유효율이 대조약(Lamivudine 100mg, Entecavir 0.5 mg, Adefovir 10 mg)의 유효율에 비해 우등하다는 것을 평가하기 위한 우등성 임상시험(superiority clinical trial)이다. 각각의 치료효과에 의한 log10 HBsAg은 independent t test를 이용하여 비교한다. e항원 혈청전환률과 소실률, s항원 혈청전환률과 소실률 등 이차 결과변수에 대한 분석은 로지스틱 회귀분석(logistic regression)을 이용하여 분석한다. 본 임상시험에 대한 유효성 및 안정성 분석은 Intent-to-treat (ITT)분석을 원칙으로 한다.

12. 부작용을 포함한 안전성의 평가기준, 평가방법 및 보고방법

1) 이상 반응의 정의

이상반응(Adverse Event, AE)이라 함은 임상시험에 사용되는 의약품을 투여 받은 대상자에서 발생한, 바람직하지 않고 의도되지 않은 징후(sign, 예 실험실적 검사치의 이상), 증상(symptom), 질병을 말하며, 해당 임상시험에 사용된 의약품과 반드시 인과관계를 가져야 하는 것은 아니다. 따라서 임상시험 연구자는 어떤 이상반응이라도 발생할 경우 환자가 이를 보고하도록 지시한다. 이 정의는 병발성의 질병이나 손상, 기존 상태의 악화를 포함한다. 예기치 않은 이상반응이란 현재 허가된 임상시험에 사용되는 의약품들의 자료집에서 그 성질, 중증도, 혹은 빈도가 확인되지 않는 이상반응을 말한다.

임상시험 기간 동안에 발생한 모든 이상반응은 임상시험용의약품의 투약과는 관계없는 것이라도 시험자의 정상적인 의료행위에 따라 대상자의 의무기록과 증례기록서의 이상반응란에 기록되어야 한다. 시험자는 징후, 증상 그리고/또는 그 밖의 임상적 정보에 기초하여 이상반응에 대한 진단을 내려야 한다. 이 경우 개별적인 징후나 증상이 아니라 진단명을 이상반응으로 문서화하여 기록한다. 명확한 진단이 이루어졌을 경우, 비정형적이거나 과도하게 표현되지 않았다면 개별 징후와 증상은 별도의 사건으로 보고되지 않아도 된다. 명확한 진단명이 없다면, 개별 징후와 증상은 모두 각각 기록되어야 한다.

임상적으로 중요한 실험실적 이상소견이나 다른 비정상적 측정치가 이상반응에 해당 되면 증례기록서의 이상반응란에 기입해야 한다. 모든 이상반응은 해결되거나 상태가 안정화되거나 이상반응이 설명 될 수 있거나 또는 더 이상 추적조사를 할 수 없을 때까지 추적조사가 되어야 한다.

시험자가 이상반응이 해결 또는 안정화되었다거나 또는 원인에 대해 설명할 수 있는지 확인하기 전에 대상자에 대한 추적조사가 불가능할 경우 임상시험실시기관은 정보를 취하기 위해 대상자와 계속 연락을 시도(적어도 2번) 하였다는 기록을 대상자의 의무기록과 증례기록서의 일반적인 Comment 란에 문서로 남겨야 한다. 이러한 기록에는 날짜와 통신수단이 포함되어야 한다.

2) 중증도 평가 기준

발생한 이상반응 중 진단의학적 검사에서 발생한 이상반응은 WHO 이상반응 기준에 따라 분류하며, 이에 포함되지 않는 이상반응은 Spiker 등의 3단계 분류법을 이용한다.

중증도 표시	내 용
경증(mild)	처치가 필요하지 않고 대상자의 정상생활(기능)을 크게 저해하지 않는 경우
중증도(moderate)	대상자의 정상 생활(기능)을 유의하게 저해하며, 처치가 필요할 수도 있으며 처치 후 회복되는 경우
중증(severe)	심한 이상반응으로 고도의 처치가 필요하며, 후유증이 남는 경우나 생명의 위협을 주는 경우

3) 중대한 이상반응 / 이상약물반응의 정의 및 보고 기준

① 정의

중대한 이상반응/이상약물반응(Serious AE/ADR)이라 함은 임상시험에 사용되는 의약품의 임의의 용량에서 발생한 이상반응 또는 이상약물반응 중에서 다음 각목의 1에 해당하는 경우를 말한다.

㉮ 사망

㉯ 생명을 위협하는 경우: 사건 발생 시점에서 대상자가 즉각적인 죽음에 처할 수 있다고 판단되는 경우를 말하며, 보다 심각한 상태로 발전했다면 사망을 초래 했을지도 모르는 이상반응은 심각한 것으로 간주 하지 않는다.

㉰ 입원 또는 입원기간의 연장이 필요한 경우: 의학적 이유로 지속적인 관찰을 위해 입원을 요하는 경우를 말한다. 입원은 의학적 이유로 병원에 공식적으로 입원하는 경우를 말하며 응급실에 방문 한 것 중 의학적으로 입원이 필요로 되는 상황만 포함된다. 또한 대상자의 개인 사유 및 선택적 의사로 입원을 하였을 경우에는 포함되지 않는다.

㉱ 불구나 기능저하를 초래하는 경우

㉲ 선천적 기형이나 이상을 초래할 경우

㉳ B형 간염의 악화는 중대성에 대한 상기 기준에 포함되지 않더라도 의학적 또는 외과적 치료를 필요로 하는 경우 중대한 이상반응으로 포함한다.

② 보고 기준

㉮ 모든 중대한 이상반응은 임상시험용의약품과의 관련성 여부와 상관없이 연구자가 인지한 시점에서 근무시간으로 1일 이내에 임상시험심사위원회와 한국 로슈(전화 02-3451-3844, 팩스 02-557-7201)에 보고해야 한다. 최초 보고는 가능한 현재 질환 및 중대한 이상반응, 이상반응과 시험약과의 인과관계에 대한 평가를 포함하여 가능한 완전해야 한다.

SUSAR 보고

ⓐ 사망을 초래하거나 생명을 위협하는 경우 : 책임연구자가 이 사실을 보고받거나 알게 된 날로부터 7일 이내. 다만, 이 경우 상세한 정보를 최초 보고일로부터 8일 이내에 추가 보고

ⓑ 다른 모든 중대하고 예상하지 못한 이상약물반응 : 책임연구자가 이 사실을 보고받거나 알게 된 날로부터 15일 이내

㉯ 중대한 이상반응을 나타낸 모든 대상자는 그 결과를 추적 관찰해야 하며, 책임 연구자는 상기 보고와 관련하여 추가적인 안전성 정보를 주기적으로 해당 이상 약물 반응이 종결(해당 이상약물반응의 소실 또는 추적조사의 불가 등)될 때까지 보고하여야 한다.

4) 인과관계 판단 기준

① 시험약 투여와의 관련유무

시험약 투여와 이상반응 발생의 관련 정도는 연구자에 의해 결정된다. 그러나 아래의 기준은 시험자가 시험약의 투여와 이상반응 발생의 관련 정도를 측정하는데 도움을 주고자 현재 가용한 정보에 근거하여 마련한 참고 기준이다. 각 항목과 그 구성요소와의 관련도(횟수 또는 강도 측면에서)가 클수록 시험약 투여와 이상반응의 관련도 역시 높아진다.

㉮ 노출 정도 (Exposure)
대상자가 실제로 약물을 투여 받은 근거자료가 있는가? (예: 믿을만한 약물 복용력,인정할 만한 순응도 측정, 예상된 약리학적 효과, 생체 내 약물/대사체 측정 등)

㉯ 시간적 순서 (Time Course)
ⓐ 이상반응의 발생시 시험약 투여 후 적당한 시간적 순서에 따라 나타났는가?
ⓑ 이상반응 발생 시점이 시험약 투여에 의한 효과와 잘 들어맞는가?

㉰ 개연성 (Likely Cause)
시험약이 대상자가 가지고 있던 근원질환(underlying disease), 다른 약물 혹은 다른 숙주-환자 요인(host-environment factor) 등, 다른 병인론 보다 이상반응을 설명하는 원인으로서 더욱 합리적인가?

㉱ 투여중단 (Dechallenge)
이상반응이 시험약의 투여 중단 또는 감량에 의하여 소실되거나 개선되는가? (주: 이 항목은 이상반응이 사망이나 영구적 불구를 초래한 경우, 또는 이상반응이 시험약의 계속적인 사용에도 불구하고 소실되거나 개선되는 경우에는 적용할 수 없다.)

㉲ 재투여 (Rechallenge)
이상반응이 시험약의 반복 투여에 의해 재발하거나 악화되지는 않는가, 또는 대상자가 동일한 시험약 또는 연구약과 유사한 계열의 약물에 유사한 이상반응을 일으킨 과거력이 있는가? (주: 이 항목은 이상반응이 사망이나 영구적 불구를 초래한 경우에는 적용할 수 없다)

㉳ 시험약 profile의 일관성
이상반응의 임상적/병리학적 발현이 동등한 또는 유사한 계열에 해당하는 약물의 약리학 또는 독성학에 관한 기존의 지식과 일치하는가?

② 시험약 유무와의 관련성 평가

㉮ 명확히 연관이 있음(Definitely related)
ⓐ 시험약을 투여하였다는 증거가 있는 경우
ⓑ 시험약 투여와 이상반응 발현의 시간적 순서가 타당한 경우
ⓒ 이상반응이 다른 어떤 이유보다 시험약 투여에 의해 가장 개연성 있게 설명되는 경우
ⓓ 투여 중단으로 이상반응이 사라지는 경우
ⓔ 재투여 (rechallenge, 가능한 경우에만 실시) 결과가 양성인 경우
ⓕ 이상반응이 시험약 또는 동일계열의 시험약에 대해 이미 알려져 있는 정보와 일관된 양상을 보이는 경우

㉯ 연관이 있음(probably related)
ⓐ 시험약을 투여하였다는 증거가 있는 경우
ⓑ 시험약 투여와 이상반응 발현의 시간적 순서가 타당한 경우
ⓒ 이상반응이 다른 원인보다 시험약 투여에 의해 더욱 개연성 있게 설명되는 경우
ⓓ 투여 중단으로 이상반응이 사라지는 경우

㉰ 연관의 가능성이 있음(possibly related)
ⓐ 시험약을 투여하였다는 증거가 있는 경우
ⓑ 시험약 투여와 이상반응 발현의 시간적 순서가 타당한 경우
ⓒ 이상반응이 다른 가능성 있는 원인들과 같은 수준으로 시험약에 기인한다고 판단되는 경우
ⓓ 투여 중단으로(실시된 경우) 이상반응이 사라지는 경우

㉣ 연관이 없음(probably not related)

ⓐ 시험약을 투여하였다는 증거가 있는 경우

ⓑ 이상반응에 대해 보다 가능성 있는 원인이 있는 경우

ⓒ 투여 중단 결과(실시된 경우)가 음성이거나 모호한 경우

ⓓ 재투여 (실시된 경우) 결과가 음성이거나 모호한 경우

㉤ 명확히 연관이 없음(definitely not related)

ⓐ 대상자/환자가 시험약을 투여 받지 않은 경우, 또는

ⓑ 시험약 투여와 이상반응 발현간의 시간적 순서가 타당하지 않은 경우, 또는

ⓒ 이상반응에 대해 다른 명백한 원인이 있는 경우

㉥ "연관의 가능성이 있음(possibly related)" 이상인 경우 시험약과 인과론적 관련이 있다고 판단한다.

5) 안전성 정보 보고

시험자는 대상자 위험을 야기하는 모든 예상되지 않은 문제들을 해당 임상시험 심사위원회 또는 윤리위원회에 즉각 보고 하여야 한다. 원인을 불문한 사망 및 시험 약 투약과 연관되거나 연관이 의심되는 모든 중대한 이상반응이 이에 포함된다. 인과관계와 무관하게 모든 중대한 이상반응은 시험 임상시험심사위원회에 보고하여야 하며 임상시험실시기관에서 연구자 파일에 사본을 보관하여야 한다.

13. 중간 분석 및 임상 시험계획서 변경

1) 중간 분석

임상시험 개시 후 24주에 중간분석을 실시한다.

2) 시험 계획서 변경

연구자는 임상시험을 진행하던 중 계획했던 치료방법에 변경이 필요하다고 판단될 경우 임의로 치료계획을 변경하여서는 안되며, 변경 계획 절차에 따라 계획서를 변경하여야 하며 변경하는 계획서는 임상시험심사위원회에 심사를 거쳐 적용되어야 한다.

14. 임상 시험의 모니터링

임상시험관리기준을 준수하기 위해서 보건복지부 지정 간경변증 센터에서 지정하는 모니터에 의해 모니터링 절차가 수행된다. 임상시험실시기관에서 임상시험기록과 의무기록에 대한 직접 접근이 반드시 보장되어야 한다. 모니터링은 임상시험증례기록서의 완결성과 정확성을 점검하고 근거서류와의 일치성을 판단하기 위해 시험책임자가 지정한 모니터의 방문을 통하여 모니터링이 이루어진다. 모니터링 방문 외에도, 임상시험 모니터와의 잦은 의사소통(편지, 전화, 또는 팩스)을 통하여 임상시험이 계획서의 설계와 관련 규정을 따라서 수행될 수 있도록 한다. 임상시험 종료 절차는 임상시험 종료 시 임상시험 모니터에 의해 수행된다.

15. 동의서 및 대상자에 대한 보호 조치

시험자는 대상자에게 본 임상시험에 대해 충분히 설명하고, 각 대상자가 본 시험에 참여할 것인가에 대해 참여 전 충분한 시간을 제공 후 대상자로부터 반드시 자발적인 참여에 대한 문서 동의를 받아야 한다. 동의는 동의서 양식에 대상자의 서명과 날짜를 기입함으로써 문서화한다.

대상자가 법적 무능력자(예: 정신박약자, 한정 또는 금치산자 등)인 경우에는 부모나 법적 보호자 또는 법적 대리인으로부터 반드시 문서 동의를 받아야 한다.

시험자가 관계법규와 규정을 준수하고 각종 관련 문헌과 권장사항, 제안에 따라 엄격히 임상시험을 실시했음에도 불구하고, 대상자가 본 임상시험에서 사용된 시험약과 인과론적인 관련이 있는 이상반응의 발생으로 고통을 당하게 되면, 피해자 보상규약에 따른 적절한 보상 규정을 적용하도록 시험자는 노력하여야 한다.

16. 대상자 안전보호에 관한 대책

임상시험실시기관은 본 시험계획서에 규정된 대로 임상시험이 적절히 진행될 수 있도록 임상시험에 필요한 설비와 전문인력을 갖추고 안전보호에 만전을 기해야 한다.
임상시험담당자는 본 계획서에 명시된 이상반응 및 주의사항 등에 대하여 사전에 충분히 숙지하고 시험도중 중대한 이상반응 등이 발생할 경우에는 즉시 해당환자의 임상시험을 중지하고 적절한 조치를 취한 후 임상시험심사위원회 및 시험책임자에게 통보해야 한다.

17. 연구실행 계획표

Study procedure	Screening	Baseline	On peg IFN Rx<br>Weeks 4, 8, 12<br>Months 6, 9, 12	Post IFN Rx<br>3, 6, 9, 12, 15,<br>18, 24 months
Visit window	-30	0	±14	±14
대상자 동의	x			
기본정보 조사	x			
선정/제외기준검토	x	x		
병력	x			
일반혈액검사	x	x	SOC	SOC
일반화학검사, P-time	x	x	SOC	SOC
HBsAg(정량포함)/ Ab	x		X	X
기초안과 검사	x			
혈청 HBV DNA	x	x	SOC	SOC
HBeAg / Ab	x	x	SOC	SOC
HBV genotype	x			
TSH	x		SOC	SOC
Alpha-fetoprotein	x		SOC	SOC
Innate immunity study	x		TBA	TBA
복부초음파(or CT, MRI 등의 영상학적 검사)	x		SOC	SOC
무작위배정		x		
연구약물지급		x	x	
이상반응			x	
병용약물	x	x	x	
질병악화여부확인			x	x

1. HBV DNA, HBeAg/Ab, HBsAg(정량포함)/ Ab 은 3달 이내의 검사, Hematology, Serum chemistry는 1개월 이내의 검사는 스크리닝 및 베이스라인 검사로 인정한다. (스크리닝 검사를 베이스라인 검사로 인용할 수 있다.)
2. alpha-fetoprotein, 영상학적 검사는 3개월 이내는 스크리닝 검사로 인정한다.
3. 혈액학적 검사 – WBC, RBC, Hemoglobin, Platelet count.
4. 일반화학검사 – ALT, AST, ALK-P, T-Bil., Alb/Total Protein, Ca/I-P, Glu, BUN/Cr, Uric acid, Cholesterol.
5. 치료 시작시 6개월 마다 HBeAg/Ab check 하며 seroconversion이 연속 2회에서 확인이 되면 연구자 판단에 의해 중단 가능
6. 그 외의 검사 항목은 연구자의 판단 하에 진행한다.
7. SOC, standard of care
8. TBA, To be announced
9. HBsAg(정량포함)/ Ab 검사는 스크리닝시 local 검사와 함께 시행하고 그 이후 방문시에는 central 검사로만 진행한다.

18. 참고문헌

1) Ahn YO. Screening target groups for hepatitis B virus infection. InZuckerman A. ed. Hepatitis B in the Asian-Pacific Region, vol. 1. Screening, Diagnosis and Control. London: Royal College of Physicians of London, 1997;13-19.
2) Chen, C.J., et al., Risk of hepatocellular carcinoma across a biological gradient of serum hepatitis B virus DNA level. JAMA, 2006. 295(1): p. 65-73.
3) Iloeje, U.H., et al., Predicting cirrhosis risk based on the level of circulating hepatitis B viral load. Gastroenterology, 2006. 130(3): p. 678-86.
4) Yang, H.I., et al., Hepatitis B e antigen and the risk of hepatocellular carcinoma. N Engl J Med, 2002. 347(3): p. 168-74.
5) Hsu, Y.S., et al., Long-term outcome after spontaneous HBeAg seroconversion in patients with chronic hepatitis B. Hepatology, 2002. 35(6): p. 1522-7.
6) Chu, C.M., et al., Natural history of hepatitis B e antigen to antibody seroconversion in patients with normal serum aminotransferase levels. Am J Med, 2004. 116(12): p. 829-34.
7) Lin, S.M., et al., Interferon therapy in HBeAg positive chronic hepatitis reduces progression to cirrhosis and hepatocellular carcinoma. J Hepatol, 2007. 46(1): p. 45-52.
8) Niederau, C., et al., Long-term follow-up of HBeAg-positive patients treated with interferon alfa for chronic hepatitis B. N Engl J Med, 1996. 334(22): p. 1422-7.
9) Mommeja-Marin, H., et al., Serum HBV DNA as a marker of efficacy during therapy for chronic HBV infection: analysis and review of the literature. Hepatology, 2003. 37(6): p. 1309-19.
10) Schiff, E.R., et al., Long-term treatment with entecavir induces reversal of advanced fibrosis or cirrhosis in patients with chronic hepatitis B. Clin Gastroenterol Hepatol, 2011. 9(3): p. 274-276 e1.
11) Yuan, H.J., et al., The relationship between HBV-DNA levels and cirrhosis-related complications in Chinese with chronic hepatitis B. J Viral Hepat, 2005. 12(4): p. 373-9.
12) Hsu, Y.S., et al., Long-term outcome after spontaneous HBeAg seroconversion in patients with chronic hepatitis B. Hepatology, 2002. 35(6): p. 1522-7.
13) Chu, C.M. and Y.F. Liaw, HBsAg seroclearance in asymptomatic carriers of high endemic areas: appreciably high rates during a long-term follow-up. Hepatology, 2007. 45(5): p. 1187-92.
14) Liu, J., et al., Incidence and determinants of spontaneous hepatitis B surface antigen seroclearance: a community-based follow-up study. Gastroenterology, 2010. 139(2): p. 474-82.
15) Liaw, Y.F., M.R. Brunetto, and S. Hadziyannis, The natural history of chronic HBV infection and geographical differences. Antivir Ther, 2010. 15 Suppl 3: p. 25-33.
16) Fattovich, G., et al., Delayed clearance of serum HBsAg in compensated cirrhosis B:relation to interferon alpha therapy and disease prognosis. European Concerted Action on Viral Hepatitis (EUROHEP). Am J Gastroenterol, 1998. 93(6): p. 896-900.
17) van Zonneveld, M., et al., Long-term follow-up of alpha-interferon treatment of patients with chronic hepatitis B. Hepatology, 2004. 39(3): p. 804-10.
18) Lampertico, P., et al., Long-term suppression of hepatitis B e antigen-negative chronic hepatitis B by 24-month interferon therapy. Hepatology, 2003. 37(4): p. 756-63.
19) Keeffe, E.B., et al., A treatment algorithm for the management of chronic hepatitis B virus infection in the United States: 2008 update. Clin Gastroenterol Hepatol, 2008. 6(12): p. 1315-41; quiz 1286.
20) Gish, R.G., et al., Entecavir therapy for up to 96 weeks in patients with HBeAg-positive chronic hepatitis B. Gastroenterology, 2007. 133(5): p. 1437-44.
21) Jung, Y.K., et al., Change in serum hepatitis B surface antigen level and its clinical significance in treatment-naive, hepatitis B e antigen-positive patients receiving entecavir. J Clin Gastroenterol, 2010. 44(9): p. 653-7.
22) Brunetto, M.R., et al., Hepatitis B virus surface antigen levels: a guide to sustained response to peginterferon alfa-2a in HBeAg-negative chronic hepatitis B. Hepatology, 2009. 49(4): p. 1141-50.
23) Hadziyannis, S.J., etal., Long-term therapy with adefovir dipivoxil for HBeAg-negative chronic hepatitis B. N Engl J Med, 2005. 352(26): p. 2673-81.

24) Liu, F., et al., Poor durability of lamivudine effectiveness despite stringent cessation criteria: A prospective clinical studyin hepatitis B e antigen-negative chronic hepatitis B patients. J Gastroenterol Hepatol, 2011. 26(3): p. 456-60.
25) Fung, J., et al., The duration of lamivudine therapy for chronic hepatitis B: cessation vs. continuation of treatment after HBeAg seroconversion. Am J Gastroenterol, 2009. 104(8): p. 1940-6; quiz 1947.
26) Lee, H.W., et al., Lamivudine maintenance beyond one year after HBeAg seroconversion is a major factor for sustained virologic response in HBeAg-positive chronic hepatitis B. Hepatology, 2010. 51(2): p. 415-21.
27) Chang, T.T., et al., Entecavir treatment for up to 5 years in patients with hepatitis B e antigen-positive chronic hepatitis B. Hepatology, 2010. 51(2): p. 422-30.
28) Leung, N.W., et al., Extended lamivudine treatment in patients with chronic hepatitis B enhances hepatitis B e antigen seroconversion rates: results after 3 years of therapy. Hepatology, 2001. 33(6): p. 1527-32.

4. 피험자 설명문 및 동의서

피험자 설명문 및 동의서

이 동의서는 여러분에게 이번 임상연구에 대한 정보를 제공하기 위하여 제작되었습니다. 본 연구의 참여 결정은 귀하의 의사에 달려 있습니다. 모든 사항은 귀하의 자유의사에 따라 참여를 결정하거나 포기를 결정할 수 있습니다. 또한 귀하의 결정으로 인한 어떠한 불이익도 받지 않을 것입니다. 아래의 설명을 읽어 보신 후 임상시험에 참여를 원하시면 자발적으로 서명 동의를 하신 분에 한하여 임상시험을 진행하게 됩니다.

1. 연구 제목: 장기간 뉴클레오타이드 제재 유지요법 중인 e항원 양성 만성 B형간염환자에서 페그인터페론 48주 투여에 따른 표면항원 정량 변화양상과 e항원 혈청전환 관련성에 대한 연구

2. 본 연구의 시험책임자 및 시행 기관:
허　정
부산대학교병원
주소: 부산광역시 서구 구덕로 305, 602-739

3. 임상연구의 목적: 장기간 뉴클레오타이드 제재 유지요법 중인 e항원 양성 만성 B형간염환자를 대상으로 페그인터페론 48주 투여를 통한 표면항원 정량 변화양상과 e항원 혈청전환 관련성을 평가하고자 합니다.

만성 B형간염은 심각한 전세계적 보건 문제로 만성 간질환, 간경변증, 원발성 간암을 일으키는 주된 요인입니다. 1998년 투약이 간편하고 부작용이 적은 경구용 항바이러스제로 라미부딘이 소개된 이후 여러 항바이러스제들이 새롭게 개발되고 있으며 이들은 바이러스의 복제를 억제하고 간효소 수치를 정상화시키며 조직학적 소견을 개선시킨다고 알려져 있습니다. 경구용 항바이러스제를 사용하여 바이러스의 증식이 억제된 후에 언제까지 치료를 지속해야 하는지에 대해서는 아직 정해진 것이 없습니다. e항원 음성 간염은 치료 종결 후 대부분 재발을 하며, e항원 양성 간염에서는 혈청전환 후에도 치료를 지속한 경우 바이러스 증식 억제 지속률이 높아지나 e항원 혈청전환률은 1년에 20% 내외에 불과하고 상당수의 환자는 B형간염바이러스 DNA가 병원에서 시행하는 B형간염바이러스 DNA PCR 감도 이하로 낮게 측정되어도 수 년간 계속 HBeAg 양성을 유지하며, 이런 경우 경구용 항바이러스제를 투여 중지하면 대부분 치료이전의 상태로 되돌아 갑니다.

최근 치료평가의 대체지표로 표면항원에 대한 연구가 많이 이뤄지고 있습니다. B형간염의 자연경과에서 표면항원의 혈청소실률은 연간 1~2%정도 일어나고, B형간염바이러스 DNA 감소와 관계되어 간경변증, 간세포암종 등 간 관련 합병증의 가능성이 감소합니다. 과거 인터페론 알파 치료를 통해 바이러스 증식이 억제된 환자에서 표면항원 혈청소실률이 높았고 표면항원 혈청소실이 일어난 환자에서 간기능소실, 간세포암종 등과 같은 간 관련 사망률의 감소를 보였지만, 궁극적인 완치를 의미하는 표면항원의 소실률은 매우 낮은 편입니다.

표면항원의 혈청소실률은 경구용 항바이러스 치료 군보다 인터페론 치료 군에서 더 높지만, 경구용 항바이러스제 사용 기간이 길어지면 표면항원의 소실률도 인터페론 치료만큼 증가될 수도 있을 가능성은 있다. 페그인터페론(페가시스) 치료 후 표면항원 역가의 감소와

B형간염바이러스 DNA 감소 사이에 상관관계를 보이고 있어, 표면항원 혈청소실 뿐만이 아니라 혈청 내 역가에 대한 연구가 활발히 이뤄지고 있어 향후 B형간염 치료의 좋은 지표로 이용될 것으로 기대됩니다.

본 임상 연구는 장기간 경구용 항바이러스제재를 투여받아 B형간염바이러스 DNA가 PCR감도 이하로 낮게 측정되는 e항원 양성 간염 환자에서 장기간 페그인터페론 알파 치료를 통해 표면항원 정량변화와 e항원 형성 및 소실(혈청전환)과의 상관성을 알고자 합니다.

이에 본 연구에서는 장기간 뉴클레오타이드 제재 유지요법 중인 e항원 양성 만성 B형간염환자를 대상으로 페그인터페론 48주 투여를 통한 표면항원변화를 규명하여 e항원 형성 및 소실(혈청전환)과 관계를 확인하여 경구용 항바이러스제재 투여 중단법을 연구하고자 합니다.

4. 연구 참여 기준:

본 연구에 참여하기 위해서 귀하는 만 18세 이상의 연령이어야 하고, 장기간 뉴클레오타이드 제재 유지요법 중인 e항원 양성 만성 B형간염환자를 대상이며, 그 밖에는 건강한 상태이어야 합니다. 연구 관계자는 귀하의 병력, 신체검사, 일반적인 안전성 실험실 검사(혈액에 대한 실험실 검사), 그리고 선별과정과 첫 약물 투여 전에 시행되는 다른 검사 결과들을 근거로 본 연구가 귀하에게 적합한지를 평가할 것입니다.

5. 본 연구에 사용되는 약제의 종류

본 연구에서 사용되는 약제는 다음 약제가 될 것이며 이 약제들의 효능과 안전성은 이미 보고되어 있습니다. 약제의 선택은 아래 페가시스와 기존 병용 항바이러스 제재가 1:1의 비율로 무작위로 선정됩니다.

페가시스

1) 약품명/상품명: Peginterferon α2a /Pegasys®
2) 원료 약품/성분의 분량: 1 프리필드 시린지(0.5 mL) 중 주성분 180 ug 함유
3) 제형: 프리필드 시린지
4) 사용방법: 1 주 1 회 피하투약
5) 제약회사/제조사: 한국로슈

제픽스

1) 약품명/상품명: Lamivudine/Zeffix®
2) 원료 약품/성분의 분량: 1 정 중 100 mg 함유
3) 제형: 필름코팅정
4) 사용방법: 1 일 1 회 경구투약
5) 제약회사/제조사: 글락소 스미스클라인

바라크루드

1) 약품명/상품명: Entecavir/Baraclude®
2) 원료 약품/성분의 분량: 1 정 중 0.5 mg 함유
3) 제형: 필름코팅정
4) 사용방법: 1 일 1 회 경구투약
4) 제약회사/제조사: 한국비엠에스제약

헵세라

1) 약품명/상품명: adefovir/Hepsera®
2) 원료 약품/성분의 분량: 1정 중 10 mg 함유
3) 제형: 필름코팅정
4) 사용방법: 1일 1회 경구투약
4) 제약회사/제조사: 글락소 스미스클라인

6. 이 연구에 참여하게 된다면 어떤 치료가 이루어지는가?

본 연구에 참여하는 경우 무작위(동전을 던져 한 곳으로 배정하는 방식으로 한 곳에) 배정함에 따라 페가시스 또는 기존 경구용 항바이러스 제재를 투여하게 됩니다. 약제의 투약은 페가시스 군에서는 48주간 지속하게 되며 일정 간격으로 치료 반응을 판정하기 위한 혈액검사 등을 실시 받게 됩니다. 본 연구는 만성 B형 간염의 치료에 이미 효과가 입증된 치료약들 중 무작위(임의로)로 선택한다는 점 이외에는 귀하의 진단 및 치료 과정에 전혀 영향을 주지 않습니다. 참고로 페가시스의 경우 현재의 상태에서는 국내 보험심사 기준에서는 이를 급여로 인정하고 있지 않고 있습니다.

7. 본 연구에 사용되는 약제의 부작용

본 연구에 사용되는 약제의 일반적으로 알려진 부작용은 다음과 같습니다. 그러나 이러한 부작용이 나타나는 빈도는 드물며 일반인에 비하여 특별히 더 흔한 것도 아닙니다. 만약의 가능성에 대비하여 이러한 부작용에 대한 모니터링(지속적인 관찰)을 같이 시행합니다.

1) 페가시스(Pegasys)

1. 일반증상 : 피로, 근육통, 경직, 주사부위 국소반응, 무력증, 통증, 인플루엔자양 증상, 권태, 졸음증, 떨림, 열성 홍조, 허약, 단순포진. 특히 인플루엔자양증상(피로, 발열, 오한, 식욕부진, 두통, 관절통, 근육통, 발한 등)이 나타나며, 이들 증상은 아세트아미노펜에 의해 부분적으로 억제된다. 대개는 감량으로 부작용의 정도가 감소된다.
2. 소화기계 : 식욕부진, 구역, 구토, 소화불량, 설사, 복통, 구강건조, 드물게 변비, 복부팽만감, 구내염, 설염, 운동항진증, 가슴쓰림.
3. 간장 : AST, ALT, ALP, LDH 및 빌리루빈치 상승과 같은 간기능 변화가 관찰될 수 있다.
4. 정신신경계 : 어지러움, 두통, 수면장애, 신경과민, 초조감, 불면, 졸음, 우울증, 집중력저하, 불안 등이 나타날 수 있다.
5. 말초신경계 : 때때로 지각이상, 감각저하, 미각이상, 저림, 신경질환, 진전, 등이 나타날 수 있다.
6. 혈액계: 백혈구 감소, 혈소판 감소, 적혈구 감소, 과립구 감소, 헤모글로빈 및 헤마토크릿치 감소, 빈혈, 등이 나타날 수 있다.
7. 자가면역 현상 : 자가면역 현상에 의한 것이라고 생각되는 증상[갑상선 기능이상(갑상선기능 저하증 또는 갑상성기능 항진증), 관절 류마치스의 악화 등]이 나타날 수 있다.

8. 기타 : 때때로 체중감소, 혈청단백질감소, 주사부동통, 혈당상승, 류마티양 관절염, 등이 나타날 수 있다.

2) 제픽스 (Lamivudine)

1. 10% 초과의 환자에게서 두통, 피로와 같은 증상과 오심,설사,구토 등의 위장관 증상들이 보고되고 있으며 간혹 췌장염 등이 나타나기도 합니다. 또한 말초신경염 및 감각이상, 근골격 통증이 보고되기도 합니다.
2. 1~10%의 환자에서는 중추신경계(현기증, 우울증, 발열, 오한, 불면증), 피부(발진), 위장관계(식욕부진, 위복부통증, 흉통, amylase증가), 혈액학적(중성구 감소증), 간(AST, ALT증가), 신경계와 근골격계(근육통, 관절통), 호흡기계(기침) 등이 동반될 수 있습니다.
3. 1% 미만의 환자에서는 드물게 탈모증, 아나필락시스, 빈혈, 간비대, 고빌리루빈혈증, 고혈당, CPK의 증가, 유산증, 림프절종대, 말초 신경병증, 소양증, 횡문근 융해, 무력감, 혈소판 감소증, 위염과 같은 증상이 동반될 수 잇습니다.

3) 바라크루드 (Entecavir)

1. 10% 초과의 환자에서 일시적으로 간수치가 증가(ALT 증가)할 수 있습니다.
2. 1-10%의 환자에서는 중추 신경계(두통, 피로), 내분비 및 대사계(고지혈증), 소화기계(lipase증가, amylase증가, 설사, 소화불량) 증상이 동반할 수도 있습니다.
3. 1% 미만에서는 현기증, 저알부민혈증, 불면증, 오심, 졸음, 저혈소판증, 구토 등의 증상이 동반할 수 있습니다.

4) 헵세라 (adefovir)

1. 10%이상의 환자에서 무력증보고 되었습니다.
2. 1-10%의 환자에서 두통, 복통, 구역, 위, 창자 내 가스 팽만, 설사, 소화불량이 보고되엇습니다.
3. 1% 미만에서 근육병증, 췌장염 등이 동반할 수 있습니다.

8. 연구 기록의 기밀의 보호

본 연구로부터 얻게 되는 정보는 연구자에게만 주어지게 될 것입니다. 귀하의 의무기록과 귀하가 서명한 동의서는 연구자와 연구참여팀원에게만 연구 등의 목적으로 보여질 수 있습니다. 하지만 여러분의 신원을 파악할 수 있는 기록은 비밀로 보장될 것이며 건강 정보는 여러분의 성명을 가린 채로 제공되기 때문에 자료상으로 여러분이 어디에 사는 누구인지, 어떤 사람인지 알 수 없습니다. 또한 임상연구의 결과가 출판될 경우에도 여러분의 신상정보는 비밀 상태로 유지될 것입니다.

여러분은 언제라도 임상연구 담당자에게 통보하여 동의를 취소할 수 있으며, 이런 경우 임상연구 담당자는 여러분의 의학 정보를 더 이상 사용할 수 없습니다.

9. 본 연구 참여와 관련된 비용

본 연구에 참여함으로써 귀하에게 추가적으로 발생하는 비용은 전혀 없습니다. 연구 기간 중 귀하는 B형 간염 환자의 추적 검사 시에 시행되는 필수적인 검사만 시행할 것이며 이는

연구 참여와 상관없이 질병의 진행 및 간암 검진을 위하여 시행되는 기본적인 검사입니다. 따라서 임상시험을 위해 발생하는 추가 비용은 없다는 것입니다. 본 연구는 만성 B형 간염의 치료에 이미 효과가 입증된 약제를 사용하는 것으로 연구에 참여하시게 되면 무작위 배정에 따라 페가시스 군에 배정이 된 경우 약물을 무상으로 공급받을 것입니다.

10. 부작용에 대한 보상기준

본 연구에서 시험자가 관계법규와 규정을 준수하고 각종 관련 문헌과 권장사항, 제안에 따라 엄격히 임상시험을 실시할 것입니다.

진행성 간질환 환자에서 위약에 비해 항바이러스제 투여 시 효과는 이미 확립된 상태입니다. 본 연구에 이용되는 약물은 이미 효과 및 안정성에 대한 자료들이 보고되어 있어 현재 국내뿐만 아니라 전세계적으로 B형 간염 치료에 널리 적용되고 있어 연구 중 귀하에게 발생한 부작용에 대해서는 따로 보상기준을 마련하지 않았습니다.

11. 기타사항

1) 본 연구에서 귀하의 참여는 언제든지 다음과 같은 이유로 시험책임자에 의해 귀하의 동의 없이 종료될 수 있습니다.

- 시험자가 귀하의 건강과 안전을 위하여 필요하다고 판단될 때,
- 귀하가 연구의 지침을 따르지 않았을 때,
- 연구팀에서 연구의 중단을 결정하였을 때, 또는 행정상의 이유

2) 만일 귀하가 본 연구와 관련될 수 있는 이상반응 또는 손상을 경험하거나, 어떤 이유로든 의학적 치료를 받기 위해 예정에 없던 방문을 할 경우에는, 만일 귀하가 이 연구의 진행 절차 등에 관해 문의할 사항이 있는 경우에는 본 임상시험의 시험책임자인 허 정 교수(051-240-7869) 또는 시험담당자 우현영 조교수(051-240-7869)에게 연락하여 주십시오. 참가자로서의 귀하의 권리 등에 관해 문의할 사항이 있는 경우에는 본 병원의 임상시험심사위원회(051-240-XXXX)로 전화하여 문의하여 주십시오.

3) 기타 피험자의 안전보호에 관하여 필요한 사항: 본 연구는 임상시험 심사위원회에서 본 연구의 윤리적, 법적 요건을 충분히 검토하여 승인한 임상시험계획서에 의하여 시행되며 또한 시험의 전 과정에 걸쳐 KGCP(의약품 임상시험 관리 기준) 및 임상연구에서 의사들의 지침인 헬싱키 선언의 근본정신을 준수하게 될 것입니다. 본 연구도중에 귀하의 인권에 침해가 발생했을 경우 임상시험 심사위원회 또는 보건당국에 통보될 것입니다.

피 험 자 동 의 서

임상연구 제목: 장기간 뉴클레오타이드 제재 유지요법 중인 e항원 양성 만성 B형간염환자에서 페그인터페론 48주 투여에 따른 표면항원 정량 변화양상과 e항원 혈청전환 관련성에 대한 연구 유효성 비교를 위한 무작위 배정, 공개 연구

아래 내용을 읽으시고 내용을 완전히 이해하시면 네모 칸에 표시하여 주십시오.

□ 본인은 이 동의서를 읽었고, 내용을 충분히 이해합니다.

□ 본인은 담당의사로부터 자세하게 설명을 듣고 궁금한 사항이 있으면 질문을 하였고 적절한 답변을 들었습니다.

□ 본인은 자발적으로 이 연구에 참여합니다.

□ 본인은 이 동의서에 기술된 바에 따라 본인의 건강정보를 사용하고 공유하는 것을 허락합니다.

□ 본인은 임상연구 기간 중 언제라도 중도에 임상연구 참여를 거부하거나 중단할 수 있습니다. 또 본인은 이 연구 참여를 중단하더라도 본인에게 어떠한 불이익도 없다는 것을 알고 있습니다.

□ 본인은 자유로운 의사에 따라 임상연구 참여를 요청하여 동의서 사본 1부를 수령합니다.

서 명

본 임상시험에 참여하기 위하여 귀하 또는 법적 대리인은 서명항목에 서명을 하고 날짜를 적어야만 합니다.

년 월 일

피험자	성명	(서명)
	주소	
연구자	성명	(서명)

(이하 해당되는 경우)

보호자 또는 대리인	성명	(서명)
	관계	
	주소	

5. 증례기록서

6. 주연구자 이력서

Version 2.3 2017-07
Roll_Over study
