# Supplementary material for: Effect of switching from nucleos(t)ide maintenance therapy to PegIFN alfa-2a in patients with HBeAg-positive chronic hepatitis B: A randomized trial
Source: PLoS One. 2022 Jul 22;17(7):e0270716. doi: 10.1371/journal.pone.0270716 (PMC9307167; doi:10.1371/journal.pone.0270716)
Supplement: S2 File — (PDF) [file pone.0270716.s014.pdf]

IRB Materials for approval application

A study to evaluate the dynamics changes of HBsAg quantity and its relation with HBeAg seroconversion following 48 weeks pegylated-interferon-alpha treatment in patients with HBeAg positive chronic hepatitis B after long term nucleos(t)ide analogue maintenance therapy

Department of Gastroenterology, Pusan National University Hospital

Jeong Heo

Version 2.3 2017-07
Roll-Over study

Contents

1. Clinical Research Review Request Form

2. Summary of Clinical Research Protocol

3. Clinical research protocol

4. Subject explanation and consent

5. Case Report-Attachment

6. Resume of main researcher

1. Clinical Research Review Request Form

Submitting Organization : ▣ Pusan National University Hospital

Deliberation type : ▣ Initial deliberation □ Complementary deliberation

Clinical Research Review Request Form

To : Pusan National University Hospital Director

Reference : Chairman of the Clinical Research Review Committee

Research Organizer	▣ Researcher- led □ Client- led					
Project Title	Korean	장기간 뉴클레오타이드 제재 유지요법 중인 e항원 양성 만성 B형간염환자에서 페그인터페론 48주 투여에 따른 표면항원 정량 변화양상과 e항원 혈청전환 관련성에 대한 연구				
	English	A study to evaluate the dynamics changes of HBsAg quantity and its relation with HBeAg seroconversion following 48 weeks pegylated-interferon-alpha treatment in patients with HBeAg positive chronic hepatitis B after long term nucleos(t)ide analogue maintenance therapy				
	Protocol No.				Version No.	Version 2.0
Investigator	Principal Investigator	Affiliation	Position	Name	Phone	e-mail
		Department of Gastroenterology, Pusan National University Hospital	Associate Professor	Jeong Heo	240-7869	jheo@pusan.ac.kr
	Sub-I	Department of Gastroenterology, Pusan National University Hospital	Assistant Professor	Hyun Young Woo	240-7895	who54@hanmail.net

<table>
<tr><td rowspan="5">Research content</td><td colspan="5">▣ Clinical trial (▣ , □ Health functional food , □ Medical device , □ Medical practice (procedure , surgery , diagnosis method) □ Gene therapy research , □ Embryonic stem cell research □ Cell therapy research □ Others) □ Observational study (□ Clinical information □ Clinical sample □ Survey)□ Others</td></tr>
<tr><td>Study drug</td><td colspan="2">Generic name:
Peginterferon α2a
Lamivudine
Entecavir
Clevudine
Adefovir
Tenofovir</td><td colspan="2">Product name:
Pegasys®
Zeffix®
Baraclude®
Levovir®
Hepsera®
Viread®</td></tr>
<tr><td rowspan="2">Clinical trial</td><td colspan="4">▣ Academic use □ Domestic permission (KFDA) □ Overseas permission (country name :) □ Not applicable</td></tr>
<tr><td>Phase</td><td colspan="3">□ Ⅰ □Ⅱ(a , b) ▣Ⅲ □Ⅳ □ biological equivalency □ other ()</td></tr>
<tr></tr>
<tr><td rowspan="4">Multi-institution joint research</td><td colspan="5">□ International ▣ Domestic</td></tr>
<tr><td colspan="2">Number of participating countries 1 country</td><td colspan="2">Organizations can participate 1 Centers</td><td>Domestic institutions 3 Centers</td></tr>
<tr><td colspan="5">□ All PI (Country : Name of Center : Name of Manager :)</td></tr>
<tr><td colspan="5">▣ Korean P.I (Organization : Pusan National University Hospital Director Name : Jeong Heo).
Kyungpook National University Hospital / Professor Won-Young Tak
Keimyung University Dongsan Medical Center / Professor Jae-Seok Hwang
Yeungnam University Medical Center / Professor Heon-Ju Lee</td></tr>
<tr><td>Number of research subjects</td><td colspan="2">Total : 144 patients (Domestic : 144 patients / 0 foreign patient)</td><td colspan="3">Assignment to this institution : 70 patients</td></tr>
<tr><td>Acquisition of subject consent</td><td colspan="5">▣ needed □ not needed (written explanation submitted)</td></tr>
<tr><td>Study period</td><td>Start date</td><td>2012 years Feb 01</td><td>Expected end date</td><td colspan="2">2020 years Dec 31</td></tr>
<tr><td>Research expenses</td><td>cash</td><td>Total won (including overhead costs)</td><td>cash</td><td colspan="2"></td></tr>
<tr><td>Research fund support organization</td><td colspan="5">□ Government agency ( ) ▣ Enterprise (Roche Korea)
□ Other ( ) □ Individual ( ) □ None</td></tr>
<tr><td>Research</td><td colspan="2">Company name :</td><td colspan="3">Business registration number :</td></tr>
</table>

<table>
<tr><td>sponsor</td><td>Representative
(position):</td><td>Name :   (sign)</td></tr>
<tr><td>The need for research
And overview</td><td colspan="2">In patients with chronic hepatitis B, HBV DNA and HBeAg is proliferation of virus, high serum HBV DNA as an index of the active hepatitis HBeAg-positive hepatitis B increases the risk of liver cirrhosis and hepatocellular carcinoma.
Patients with HBeAg serum loss or conversion in the natural course of hepatitis B have a low incidence of cirrhosis or hepatocellular carcinoma and have a good long-term prognosis. Reduction of HBV DNA has been proposed as a more important therapeutic target. In patients with active hepatitis with increased HBV DNA, reducing HBV DNA levels through treatment improves tissue findings, normalizes seroconversion of HBeAg, and normalizes ALT and can inhibit the progression of hepatitis.
It is not yet established how long treatment should be continued after viral proliferation is suppressed using oral antiviral agents. HBeAg-negative hepatitis mostly recurs after treatment is terminated, and in HBeAg-positive hepatitis, if the treatment is continued after seroconversion, the persistence of inhibition of viral proliferation increases. Even if it is measured as low as the commercial HBV DNA PCR sensitivity, HBeAg remains positive for several years.
Recently, many studies have been conducted on HBsAg as an alternative index for treatment evaluation. In the natural course of hepatitis B, the rate of serum loss of HBsAg occurs about 1~2% per year, and the likelihood of liver-related complications such as cirrhosis and hepatocellular carcinoma decreases due to a decrease in HBV DNA. The HBsAg serum loss rate was high in patients whose viral proliferation was suppressed by interferon alpha treatment, and liver-related mortality rates such as liver function loss and hepatocellular carcinoma were decreased in patients with HBsAg serum loss, but the loss rate of HBsAg was very low.
The serum loss rate of HBsAg was higher in the interferon treatment group than in the oral antiviral treatment group, but if the duration of use of the oral antiviral drug is prolonged, the loss rate of HBsAg may be increased as well as the interferon treatment. There is a correlation between a decrease in HBsAg titer and a decrease in HBV DNA after peginterferon treatment. As a result of active research on not only HBsAg serum loss but also serum titer, it is expected to be used as a good indicator of hepatitis B treatment in the future.
In HBeAg-positive hepatitis patients whose HBV DNA is measured as low as PCR sensitivity after a long-term oral antiviral drug administration, a study to determine the relationship between HBsAg quantitative change and HBeAg seroconversion through long-term peginterferon alpha treatment will be needed.</td></tr>
<tr><td>Subject information</td><td colspan="2">Research subjects : ▣ Patient □ healthy □ vulnerable subjects group (below</td></tr>
</table>

	check)					
	□ Pregnant women	□ Young / Child	□ Foreigner	□ Student at school		
	□ Employees such as research institutes, researchers , clients, etc. □ Researcher or student in charge of testing					
	□ Inmate	□ Those held in the facility	□ social stigma is taken for diseases with party			
	□ Recruitment of subjects by soldiers or military organizations					
	□ Handicapped (□ mental , □ physical , □ cognitive)					
Characteristic	Subject recruitment documents (advertising , flyer , internet , email , etc.) of use :<br>□ Yes ▣ No<br>(Use case must IRB deliberations being approved after use - the present form reference)					
	Treatment / medication / inspection of such intervention if	▣ Yes	□ No			
	Whether to use the subject's private information	▣ Yes	□ No			
	Whether samples are collected and stored	▣ Yes	□ No			
	Sample overseas leak	□ Yes	▣ No			
	Whether genetic information is collected and stored	▣ Yes<br>(Submit genetic consent form)	□ No			
	Whether there is an economic interest	□ Yes	▣ No			
	The procedure used	□ Invasive	▣ Non-invasive	□applicable requirements None		
List of submitted materials<br>(Version No.<br>Submit Attachment)	▣ Summary of plan	▣ Research proposal				
	▣ Subject explanation and consent	□ subject consent waiver explanation				
	□ Submission of genetic consent	▣ Case report				
	▣ Statement of research expenses	▣ Research Officer recent 3 years history and the career of documents				
	□ Subject recruitment announcement	□ Subject compensation agreement				
Subject cost burden	▣ Regular	□ Test	□	▣ Medical	▣	□ Other

<table>
<tr><td colspan="2"></td><td>treatment
▣ Other than normal treatment</td><td>drug
□ Test drug</td><td>Reference drug
□ Reference drug</td><td>expenses
▣ Medical expenses</td><td>Inspection fee
▣ Inspection fee</td><td colspan="2">□ Other</td></tr>
<tr><td colspan="2">Submission of interim report</td><td colspan="7">▣ Yes □ No (explanation submitted)</td></tr>
<tr><td rowspan="4">Contact</td><td rowspan="2">researcher</td><td>Company Name</td><td colspan="4">Department Name : Department of Gastroenterology, Pusan National University Hospital</td><td colspan="2">Name : Hyun Young Woo</td></tr>
<tr><td>Tel : 240-7869</td><td colspan="3">HP : 010- 9067-0411</td><td>FAX:</td><td colspan="3">E-mail: who54@hanmail.net</td></tr>
<tr><td rowspan="2">coordinator</td><td colspan="7">Name ;</td></tr>
<tr><td>Tel :</td><td colspan="3">HP :</td><td>FAX:</td><td colspan="3">E-mail:</td></tr>
</table>

Above , as the clinical research review , we ask . I will faithfully conduct research in accordance with the submitted research proposal .

Application Date : Oct. 2016

Principal Investigator Jeong Heo (sign)

※ Information to be submitted

IRB No.		Reception date		Confirm reception desk	(sign)

2. Summary of Clinical Research Protocol

◈ Summary of plan

Title	A study to evaluate the dynamics changes of HBsAg quantity and its relation with HBeAg seroconversion following 48 weeks pegylated-interferon-alp ha treatment in patients with HBeAg positive chronic hepatitis B after long term nucleos(t)ide analogue maintenance therapy
Principal Investigator	Jeong Heo, Associate Professor, Department of Gastroenterology, Pusan National University Hospital
Sub-Investigator	Hyun Young Woo, Assistant Professor, Department of Gastroenterology, Pusan National University Hospital
Implementation Institution	Pusan National University Hospital
Responsible for managing pharmacist	TBD
Joint research institute	Kyungpook National University Hospital / Professor Won-Young Tak<br>Keimyung University Dongsan Medical Center / Professor Jae-Seok Hwang<br>Yeungnam University Medical Center / Professor Heon-Ju Lee
purpose	The purpose of this study is to study the relationship between HBs Ag quantitative change and HBeAg seroconversion in patients with HBe antigen-positive chronic hepatitis B who are undergoing long-term nucleotide maintenance therapy.
Trial design overview	This study is a prospective, randomized, open-labled, multicenter clinical trial that maintains low HBV DNA levels with long-term nucleos(t)ide maintained therapy, but targets HBe antigen-positive chronic hepatitis B patients. To compare the quantitative change pattern of the surface antigen and the HBe antigen seroconversion rate.
Research drug	Pegasys<br>1) Drug name / Product name : Peg interferon α2a / Pegasys ®<br>2) Raw medicine / the component amount<br>① 1 Pre-filled syringe 180 (0.5 mL) contains 180 ug of the main ingredient<br>② 1 Pre-filled syringe 135 (0.5 mL) contains 135 ug of the main ingredient<br>③ 180 ug of main ingredient contained in 180 microgram proclick (0.5 mL)<br>④ Contains 135 ug of main ingredient in 135 microgram proclick (0.5 mL)<br>3) Formulation : Pre-filled syringe<br>① 1 pre-filled syringe 180<br>② 1 pre-filled syringe 135<br>③ 180 microgram pro click

<table>
<tr><td></td><td colspan="4">④ 135 microgram pro click
4) pharmaceutical companies / manufacturers : Roche Korea</td></tr>
<tr><td>Reference drug</td><td colspan="4">Lamivudine
1) Drug Name / Product Name : Lamivudine/Zeffix ®
2) Raw medicine / the component quantities : 1 tablet of 100 mg contains
3) Formulation : Film coated tablet
4) Pharmaceutical company / manufacturer : Glaxo Smith Kline
Entecavir
1) Drug Name / Product Name : Entecavir/Baraclude ®
2) Raw medicine / the component quantities : 1 tablet of 0.5 mg -containing
3) Formulation : Film coated tablet
4) Pharmaceutical company / manufacturer : BMS Pharmaceutical Korea
Adefovir
1) Drug Name / Trade Name : Adefovir / Hepsera ®
2) Raw medicine / the component quantities : 1 tablet of 10 mg -containing
3) Formulation : Film coated tablet
4) Pharmaceutical company / manufacturer : Glaxo Smith Kline
Clevudine
1) Drug/brand name: Clevudine/Levovir®
2) Amount of drug substance/ingredient: Contains 10 mg per tablet
3) Formulation: Hard capsule
4) Pharmaceutical company/manufacturer: Bukwang Pharmaceutical
Tenofovir
1) Drug/brand name: tenofovir/Viread®
2) Amount of drug substance/ingredient: Contains 300 mg per tablet
3) Formulation: Film coated tablet
4) Pharmaceutical company/manufacturer: Gilead Science</td></tr>
<tr><td>Target disease name</td><td colspan="2">Compensated liver function with long-term nucleotide analogue maintenance therapy are HBe antigen-positive chronic hepatitis B patients of low viremia (HBV DNA < 400 copies/mL) in patients</td><td>Corporal name</td><td>Chronic hepatitis B</td></tr>
<tr><td>Number of subjects</td><td>Total 144 people (Domestic: 144 patients, Foreigner: 0 patients)</td><td colspan="3">Assignment to this institution : 70 patients</td></tr>
</table>

	Calculation basis *<br>Assuming that the expected difference between the two groups is 0.6 from the existing literature information and the standard deviation is 1.1,<br>Here, under a significance level of 5% and power of 90%<br>The number of subjects required is 72 people per group.<br>Considering the 10% dropout rate, a total of 160 people will be recruited from 80 people per group.<br>Reference) Hou et al., Efficacy and safety Peginterferon Alfa-2a versus Adefovir Dipivoxil (ADV) in treating Lamivudine Resistant HBeAg-Positive CHB, AASLD 2008
Inclusion criteria	1. Men or women over 20 years old<br>2. Patients with chronic hepatitis B with positive HBsAg<br>3. Lamivudine, adefovir, clevudine, entecavir, tenofovir alone or the combined administration except telbivudine of at least 18 or more months of patients HBV DNA undetectable (400 copies / mL or less) are 12 patients continued over months<br>4. HBeAg- positive chronic B hepatitis<br>5. ALT levels are normal upper limit value 10 times or less when the<br>6. Baseline HBsAg quantification 100 IU/mL or more<br>7. Test about the first dose before 24- hours within the identified urine or serum pregnancy test result negative (childbearing women case). Additionally, the fertile spouses in male patients and female patients testing period and during treatment completed after 3 months for sure the contraception should be used.<br>8. Treatment and follow-up observation is possible according to this research plan, and a person who has received written consent from the person or guardian
Exclusion criteria	1. Decompensated cirrhosis (Childs BC) : spontaneous bacterial peritonitis , bleeding caused by varicose veins , showing the other signs of hepatic encephalopathy or liver function loss disease if the force<br>2. Clinical you can suggest liver cancer, if there is radiographic evidence (screeningα-fetoprotein level > 50 ng/mL, in the presence of dysplastic nodules (if the size of a nodule is larger than 1 cm on CT and MRI, or if there is a nodule larger than 1.5 cm on abdominal ultrasound)<br>3. Co- infection with HCV or HIV<br>4. When other causes of liver disease exist<br>5. Pregnant or lactating female patients<br>6. If you have been given an immunomodulatory / immunosuppressant within 6 months before registration<br>7. There is a serious disease that is considered to affect the test besides liver disease

	(eg. congestive heart failure, kidney failure, chronic pancreatitis, uncontrolled diabetes, alcoholism, malignant tumors, etc.)<br>8. If a liver transplant has been performed or a liver transplant is planned<br>9. If you have a history of hypersensitivity to interferon<br>10. If you have resistance to the nucleotide analogs currently being administered<br>11. Patients who have previously used Telbivudine
Screening	In HBe antigen-positive chronic hepatitis B patients undergoing long-term nucleos(t)ide maintenance therapy, screening is performed by applying selection and exclusion criteria for patients with serum HBV DNA of 400 copies/mL or less. Pre-treatment tests may be performed to determine the test subject's suitability screening and baseline test results, and subjects with significant abnormalities are excluded.
Research method	Screening for patients with HBe antigen-positive chronic hepatitis B infection under long-term nucleos(t)ide maintenance therapy with serum HBV DNA of 400 copies/mL or less → one-to-one random assignment to the existing antiviral or pegasus group → drug administration for 48 weeks → under treatment /Evaluate after exit
Effectiveness evaluation Item and method *	Primary endpoint<br>Changes in the quantity of HBsAg (log10 HBsAg) during administration of antiviral agents in each group<br><br>Secondary endpoint<br>1.Comparison of changes from baseline in serum HBV DNA levels and HBV DNA non-detection rates and HBV DNA <20 IU/mL during the administration of antiviral agents in each group and follow-up.<br>① Comparison of the ratio of HBV DNA <2,000 IU/mL during follow-up and administration of antiviral agents of each group<br>② Comparison of the ratio of HBV DNA <20,000 IU/mL during follow-up and administration of antiviral agents of each group<br>2. Comparison of e-antigen seroconversion rate and loss rate during the administration of antiviral agents and follow-up of each group<br>3. Comparison of s-antigen seroconversion rate and loss rate at 1 and 2 years after administration/termination of antiviral agents in each group<br>4. Changes in HBsAg quantity (log10 HBsAg) after administration/termination of antiviral agents in each group
Statistical analysis method	The primary outcome variable is the HBe antigen predictive of seroconversion in chronic hepatitis B patients. This clinical study is a superiority clinical trial to evaluate that the efficacy of the test drug (Peginterferon α2a 180 ug/mL) is superior to that of

	the reference drug (Lamivudine 100 mg, Entecavir 0.5 mg, Adefovir 10 mg). The log10 HBsAg by each treatment effect is compared using independent T test. Analysis of secondary outcome variables such as HBe antigen seroconversion rate and loss rate, and HBs antigen seroconversion rate and loss rate are analyzed using logistic regression. Effectiveness and stability analysis for this clinical trial is based on Intent-to-treat (ITT) analysis.

3. Clinical research protocol

3.1 Research Title

Korean: 장기간 뉴클레오타이드 제재 유지요법 중인 e항원 양성 만성 B형간염환자에서 페그인터페론 48주 투여에 따른 표면항원 정량 변화양상과 e항원 혈청전환 관련성에 대한 연구

English: A study to evaluate the dynamics changes of HBsAg quantity and its relation with HBeAg seroconversion following 48 weeks pegylated-interferon-alpha treatment in patients with HBeAg positive chronic hepatitis B after long term nucleos(t)ide analogue maintenance therapy

3.2 Research background and rationale

3.2.1 Research Background

About 300 million people worldwide are infected with the hepatitis B virus (HBV), and in Korea, 5.8%-10.9% of men over 20 years old and 1.5%-4.4% of women are infected. Of these, 25% suffer from serious complications related to chronic hepatitis B.

Since lamivudine was introduced as an oral antiviral agent that is easy to administer and has few side effects in 1998, several nucleosides and nucleotide structure derivatives have been newly developed, and they are known to inhibit HBV replication, normalize ALT, and improve histological findings. . The goal of these antiviral treatments is to suppress HBV DNA in the short term, normalize serum alanine aminotransferase (ALT) levels, and reduce inflammation and necrosis of liver tissue. The long-term goal is to prevent liver cirrhosis, progression to end-stage liver disease, and liver cancer.

In chronic hepatitis B patients, HBV DNA and HBeAg are indicators of viral proliferation and active hepatitis. Serum HBV DNA is high and HBeAg-positive hepatitis B increases the risk of cirrhosis or hepatocellular carcinoma. Patients with HBeAg serum loss or conversion in the natural course of hepatitis B have a low incidence of cirrhosis or hepatocellular carcinoma and have a good long-term prognosis. Reduction of HBV DNA has been proposed as a more important therapeutic target. In patients with active hepatitis with increased HBV DNA, reducing HBV DNA levels through treatment improves tissue findings, normalizes HBeAg seroconversion, and normalizes ALT and can inhibit the progression of hepatitis.

It is not yet established how long treatment should be continued after viral proliferation is suppressed by using oral antiviral agents. HBeAg-negative hepatitis mostly recurs after treatment is terminated. In HBeAg-positive hepatitis, if the treatment is continued after seroconversion, the persistence of inhibition of virus proliferation increases. Even if it is measured as low as the commercial HBV DNA PCR sensitivity, HBeAg remains positive for several years.

Recently, many studies have been conducted on HBsAg as an alternative index for treatment evaluation. In the natural course of hepatitis B, the serum loss rate of HBsAg occurs about 1~2% per year, and the

likelihood of liver-related complications such as cirrhosis and hepatocellular carcinoma decreases due to a decrease in HBV DNA. The HBsAg serum loss rate was high in patients whose viral proliferation was suppressed by interferon alpha treatment, and liver-related mortality such as liver function loss and hepatocellular carcinoma decreased in patients with HBsAg serum loss, but the loss rate of HBsAg was very low.

The serum loss rate of HBsAg was higher in the interferon treatment group than in the oral antiviral treatment group, but it is possible that the loss rate of HBsAg may increase as much as the interferon treatment if the period of use of the oral antiviral drug is prolonged. There is a correlation between a decrease in HBsAg titer and a decrease in HBV DNA after peginterferon treatment.As a result of active research on not only HBsAg serum loss but also serum titer, it is expected to be used as a good indicator for the treatment of hepatitis B in the future.

This clinical study aims to clarify the relationship between HBsAg quantitative change and HBeAg seroconversion in HBeAg-positive hepatitis patients whose HBV DNA is measured as low as PCR sensitivity after long-term administration of oral antiviral agents through long-term peginterferon alpha treatment.

3.2.2 Rationale for clinical trial

Even if the proliferation of the virus is suppressed in HBe antigen-positive hepatitis B patients by using an oral antiviral drug, the HBeAg seroconversion rate is only around 20% per year, and if HBe antigen is positive, an oral antiviral agent should be continuously administered. In the past, short-term oral antiviral and peginterferon α2a combination/sequential therapy did not show superior efficacy compared to oral antiviral monotherapy. In the subjects whose surface antigen reduction was noticeable after α2a administration, viral activity inhibition was maintained after treatment was completed.

The purpose of this clinical trial is to study and analyze the clinical benefits of e-antigen-positive chronic hepatitis B patients undergoing long-term nucleotide maintenance therapy through peginterferon 48-week administration of the surface antigen and the relationship between e-antigen seroconversion.

3.3 Research purpose

3.3.1 Primary endpoint

Changes in the quantity of HBsAg (log10 HBsAg) during administration of antiviral agents in each group

3.3.2 Secondary endpoints

1. Comparison of changes from baseline in serum HBV DNA levels and HBV DNA non-detection rates and HBV DNA <20 IU/mL during administration of antiviral agents in each group and

follow-up

① Comparison of the ratio of HBV DNA <2,000 IU/mL during follow-up and administration of antiviral agents of each group

② Comparison of the ratio of HBV DNA <20,000 IU/mL during the administration of antiviral agents of each group and follow-up

2. Comparison of HBe-antigen seroconversion rate and loss rate during the administration of antiviral agents in each group and follow-up
3. Comparison of HBs-antigen seroconversion rate and loss rate at 1 and 2 years after administration/termination of antiviral agents in each group
4. Changes in the quantity of HBsAg (log10 HBsAg) after administration/termination of antiviral agents in each group

3.4 Research institute and duration

3.4.1 Implementing institute

Institution: Pusan National University Hospital

Address: 179, Gudeok-ro, Seo-gu, Busan (49241)

3.4.2 Implementing co-institute

Kyungpook National University Hospital / Professor Won-Young Tak

Keimyung University Dongsan Medical Center / Professor Jae-Seok Hwang

Yeungnam University Medical Center / Professor Heon-Ju Lee

3.4.3 Duration

From the start of the open study until all subjects reach 24 months of follow-up after 48 weeks of dosing (Scheduled research period: February 1, 2012 - December 31, 2020)

3.5 Criteria for selection or exclusion of subjects

3.5.1 Target patient group

The study population consisted of patients with low level of viremia (HBV DNA> 400 copies/mL) among HBe Ag-positive chronic hepatitis B patients undergoing long-term nucleotide maintenance therapy with targeted liver function. Participants in the study are assigned to the conventional oral antiviral drug administration group and the Pegasys administration group at a ratio of 1:1.

3.5.2 Inclusion criteria

1. Men or women over the age of 20

2. Chronic hepatitis B patients with positive HBsAg

3. Among patients who received lamivudine, adefovir, clevudine, entecavir, tenofovir alone or in combination for at least 18 months, excluding telbivudine, HBV DNA undetectable (400 copies/mL or less) lasted more than 12 months.

4. HBeAg positive chronic hepatitis B patients

5. If the ALT level is less than 10 times the upper limit of normal

6. Baseline HBsAg quantification 100 IU/mL or more

7. The urine or serum pregnancy test result confirmed within 24 hours before the first administration of the test drug was negative (for women of childbearing age). In addition, male and female patients with spouses of childbearing potential should use robust contraception during the study period and for 3 months after completion of treatment.

8. Treatment and follow-up observation is possible according to this research protocol, and a person who has received written consent from the person or guardian.

3.5.3 **Exclusion criteria**

1. Patients with decompensated cirrhosis (Childs B-C): Has a history of spontaneous bacterial peritonitis, bleeding due to varicose veins, hepatic encephalopathy, or other signs of loss of liver function.

2. If there is clinical or radiographic evidence suggesting hepatocellular carcinoma (screening α-fetoprotein level> 50ng/mL, if there is a dysplastic nodule (CT, MRI, if the size of the nodule is larger than 1cm or abdominal Ultrasound findings with nodules larger than 1.5 cm))

3. Concomitant infection with HCV or HIV

4. When other causes of liver disease exist

5. Pregnant or lactating female patients

6. If you have been given an immunomodulatory/immunosuppressant within 6 months prior to registration

7. There is a serious disease that is considered to affect the test in addition to liver disease (eg, congestive heart failure, kidney failure, chronic pancreatitis, uncontrolled diabetes, alcoholism, malignant tumors, etc.)

8. If a liver transplant has been performed or a liver transplant is planned

9. If you have a history of hypersensitivity to interferon

10. If you are resistant to the nucleotide analogs currently being administered

11. Patients who have previously used Telbivudine

3.6 Number of target subjects and basis for calculation

3.6.1 **Target number of subjects**

72 patients in each treatment group (144 patients in total)

3.6.2 **Calculation basis**

Assuming that the expected difference between the two groups is 0.6 and the standard deviation is 1.1 from the existing literature information, here, 72 subjects per group are required under a significance level of 5% and power of 90%. Considering the 10% dropout rate, a total of 160 people will be recruited from 80 people per group.

Reference) Hou et al., Efficacy and safety Peginterferon Alfa-2a versus Adefovir Dipivoxil(ADV) in treating Lamivudine Resistant HBeAg-Positive CHB, AASLD 2008.

3.7 **Study design and method**

3.7.1 **Overview of clinical trials**

Screening for patients with HBe antigen-positive chronic hepatitis B infection under long-term nucleotide maintenance therapy with serum HBV DNA of 400 copies/mL or less → one-to-one random assignment to the existing antiviral drug or pegasys group → drug administration for 48 weeks → under treatment /Evaluate after exit

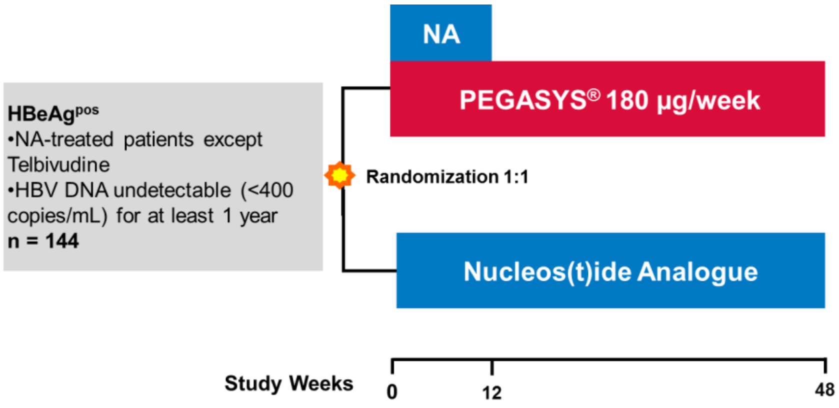

3.7.2 **Drugs subject to clinical trials**

3.7.2.1 Test drug: Peginterferon α2a

1) Drug/brand name: Peginterferon α2a /Pegasys®

2) Amount of drug substance/ingredient: 1 Pre-filled syringe (0.5 mL) contains 180 ug of the main ingredient

① 1 Pre-filled syringe 180 (0.5 mL) contains 180 ug of the main ingredient

② 1 Pre-filled syringe 135 (0.5 mL) contains 135 ug of the main ingredient

③ 180 ug of main ingredient contained in 180 microgram proclick (0.5 mL)

④ Contains 135 ug of main ingredient in 135 microgram proclick (0.5 mL)

3) Formulation: Pre-filled syringe

① 1 pre-filled syringe 180

② 1 pre-filled syringe 135

③ 180 microgram pro click

④ 135 microgram pro click

4) How to use: Subcutaneous administration once a week

5) Pharmaceutical company/manufacturer: Roche Korea

3.7.2.2 Reference drug: Lamivudine

1) Drug/brand name: Lamivudine/Zeffix®

2) Amount of drug substance/ingredient: Contains 100 mg per tablet

3) Formulation: Film coated tablet

4) How to use: Orally administered once a day

5) Pharmaceutical company/manufacturer: Glaxo Smith Kline

3.7.2.3 Reference drug: Entecavir

1) Drug/brand name: Entecavir/Baraclude®

2) Amount of drug substance/ingredient: Contains 0.5 mg per tablet

3) Formulation: Film coated tablet

4) How to use: Orally administered once a day

5) Pharmaceutical company/manufacturer: BMS Pharmaceuticals Korea, Bristol-Myers Squibb

3.7.2.4 Reference drug: Adefovir

1) Drug/brand name: adefovir/Hepsera®

2) Amount of drug substance/ingredient: Contains 10 mg per tablet

3) Formulation: Film coated tablet

4) How to use: Orally administered once a day

5) Pharmaceutical company/manufacturer: Glaxo Smith Kline

3.7.2.5 Reference drug:: Levovir

1) Drug/brand name: clevudine/Levovir®

2) Amount of drug substance/ingredient: Contains 10 mg per tablet

3) Formulation: Hard capsule

4) How to use: Orally administered once a day

5) Pharmaceutical company/manufacturer: Bukwang Pharmaceutical

3.7.2.6 Reference drug: Viread

1) Drug/brand name: tenofovir/Viread®

2) Amount of drug substance/ingredient: Contains 300 mg per tablet

3) Formulation: Film coated tablet

4) How to use: Orally administered once a day

5) Pharmaceutical company/manufacturer: Gilead Science

3.7.3 Dosage and duration of the target drug

3.7.3.1 Peginterferon α2a administration group: Peginterferon α2a 180 ug/week 48 weeks

3.7.3.2 Lamivudine administered group: Lamivudine 100 mg QD continued

3.7.3.3 Entecavir administered group: Entecavir 0.5 mg QD continued

3.7.3.4 Adefovir administered group: adefovir 10 mg QD continued

3.7.3.5 Clvudine administration group: clevudine 10 mg QD continued

3.7.3.6 Tenofovir administration group: tenofovir 300 mg QD continued

3.7.4 Concomitant Prohibited Drugs

Other drugs with antiviral effects against hepatitis B are not permitted.

However, it is allowed in the following cases.

1) When ‘clinical breakthrough’ occurs due to resistance to the drug during drug administration

* Clinical breakthrough is defined as a 'biochemical flare' in this study as a case where ALT is increased by more than twice the upper limit of normal after normal ALT is accompanied by 'virologic breakthrough'. -In this case, Adefovir rescue therapy can be performed.

* If 'virologic breakthrough' occurs during the treatment period, but the degree of 'biochemical breakthrough' is less than twice the normal upper limit, rescue therapy can be determined at the discretion of the investigator. have.

Immunosuppressants (systemic corticosteroids, Mycophenolate moferil, cyclosporine, and anti-metabolite (azathioprine, etc.) are not permitted.

During the study period, biphenyldimethyldicarboxylate (DDB) drugs are not permitted, but other drugs that enhance/protect liver function may be partially allowed at the discretion of the researcher.

3.7.5 Randomization method and blindfolding

At the beginning of the study, a one-to-one random assignment to the Peginterferon α2a-treated group and the conventional antiviral agent-administered group was conducted as an open study. In allocating drugs, a stratified probability scheme is applied. Patients should be allocated according to a

randomization table separately prepared for each hospital participating in the multicenter study. However, if the recruitment of subjects is not smooth, with the consent of all researchers, the randomization will be changed in a competitive registration method until the total number of recruitment plans is filled.

3.8 Observation item

3.8.1 Observation item

3.8.1.1 Examination

1) Screening before treatment

Subject consent, review of selection/exclusion criteria, medical history check, vital signs, physical examination, HBV DNA, serology lab (HBeAg, Anti-HBe), hematology lab (WBC, RBC, Hemoglobin, Platelet count, Differential count), serum chemistry (Calcium, Inorganic P, Glucose, BUN, Creatinine, Uric acid, Cholesterol, Total protein, Albumin, Alkaline phosphatase, AST/ALT, T. bilirubin), PT, α-fetoprotein, Abdomen U/S (or CT, MRI), Basic ophthalmic examination

(Among these tests, Alpha-fetoprotein, imaging tests, HBV DNA, serology lab tests within 3 months, Hematology lab, Serum chemistry tests within 1 month are accepted as screening and baseline tests.)

2) Every 12 weeks after starting treatment (up to 48 weeks of dosing)

Confirmation of worsening of disease, identification of adverse reactions & concomitant drugs, Serum chemistry (Calcium, Inorganic P, Glucose, BUN, Creatinine, Uric acid, Cholesterol, Total protein, Albumin, Alkaline phosphatase, AST/ALT, T. bilirubin), HBV DNA, HBsAg quantification

3) Every 24 weeks after starting treatment (up to 48 weeks of dosing)

α-fetoprotein, Abdomen U/S (or CT, MRI)

HBeAg/Ab check every 12 weeks at the start of treatment, and if seroconversion is confirmed twice in a row, it can be stopped at the discretion of the investigator.

4) Other test items are conducted under the judgment of investigator.

3.9 Predicted side effects and precautions for drug use

3.9.1 **Peginterferon α2a**

1) Do not administer to the following patients

Patients with hypersensitivity to this drug or other ingredients of this drug, patients with hypersensitivity to interferon alpha agents, patients with autoimmune hepatitis, patients with severe psychosis or history of patients with thyroid disease not controlled by medication, severe

liver Disorders, decompensated liver disease patients, patients with organ transplants other than liver, patients with uncontrolled seizures and/or central nervous system dysfunction, pregnant women, newborns and children under 3 years of age (this drug contains benzyl alcohol, warnings Reference), patients with severe heart disease, patients with severe kidney disease, patients with severe bone marrow dysfunction, patients with hypersensitivity to biological agents such as vaccines, and patients with HIV-HCV double infection with cirrhosis with a Child-Pugh score of 6 or higher.

2) Carefully administer to the following patients

   A. Patients with heart disease or its history: Although direct cardiac toxicity has not been proven, acute side effects of the drug itself (eg fever, chills), which are common after administration of this drug, may aggravate the existing heart condition.
   B. Patients with kidney, liver or bone marrow dysfunction: If there is a mild-severe dysfunction, these functions should be carefully monitored.
   C. Patients with predisposition to allergies
   D. Patients with diabetes or its medical history, family history, and impaired glucose tolerance (diabetes is more likely to worsen or develop)
   E. Patients with autoimmune disease or its predisposition
   F. Hypertension patients
   G. Patients with a neutrophil count less than 1,500/3, platelet count less than 75,000/㎣, or red blood cell count less than 10g/dl (anemia) as a baseline hematologic test
   H. Patients with lung disease (eg chronic airway obstruction disease) or a history of it
   I. Psoriasis patients (psoriasis may worsen)
   J. Patients with a history of mental illness

3) Side Effect

   A. General symptoms: Fatigue, muscle pain, stiffness, local reaction at the injection site, asthenia, pain, influenza-like symptoms, boredom, drowsiness, tremor, febrile flushing, weakness, herpes simplex. In particular, influenza-like symptoms (fatigue, fever, chills, loss of appetite, headache, joint pain, muscle pain, sweating, etc.) appear, and these symptoms are partially suppressed by acetaminophen. Usually, reducing the amount reduces the severity of side effects.
   B. Digestive system: loss of appetite, nausea, vomiting, indigestion, diarrhea, abdominal pain, gastritis, dry mouth, mouth ulcer, bleeding gums, rarely constipation, bloating, stomatitis, glossitis, hyperactivity, heartburn, peptic ulcer recurrence And gastrointestinal bleeding that is not life-threatening has been reported in several cases.
   C. Diabetes (insulin-dependent (IDDM) and insulin-independent (NIDDM)): Check regularly (blood sugar level, urine sugar, etc.) and take appropriate treatment if abnormalities appear.

D. Liver: Changes in liver function such as elevated AST, ALT, ALP, LDH and bilirubin level can be observed.

E. Mental nervous system: dizziness, consciousness disorder, confusion, headache, sleep disorder, nervous irritability, agitation, insomnia, lethargy, drowsiness, depression, loss of concentration, anxiety, memory loss, emotional disorder, mood change, nervousness, aggression, decreased libido, Premature ejaculation, irritability, light-headedness (without vertigo), urinary incontinence, convulsions, dementia-like symptoms (especially in the elderly), hallucinations, and disorientation.

F. Peripheral nervous system: Sometimes perception disorders, sensory loss, taste disorders, confusion, tingling, neurological disorders, tremors, and elevated CPK may occur.

G. Cardiovascular system: cardiomyopathy, sometimes peripheral ischemia, heart failure, angina, ECG abnormalities (extra-constriction, ventricular tachycardia, atrial fibrillation, ST depression, etc.), myocardial disorders, pain, tachycardia, bradycardia, palpitations, transient hypotension and hypertension, Limb edema, facial flushing, cyanosis, arrhythmia, palpitations, palpitations, rarely pulmonary edema, congestive heart failure, cardiac respiratory arrest and myocardial infarction have been reported.

H. Skin, mucous membranes, appendages: re-deterioration of cleft lip herpes, skin vasculitis and skin diseases, rash, eczema, itching, mild to moderate hair loss, dry skin, runny nose, nasal bleeding, erythema, toenail discoloration, psoriasis, urticaria, Photosensitive reactions, increased sweating, sweating during sleep, and systemic lupus erythematosus may occur.

I. Kidney and urinary tract: In rare cases, moderate renal injury such as acute renal failure may occur (mainly cancer patients undergoing combination treatment with renal disease and nephrotoxic drugs). Stop and take appropriate treatment. Proteinuria, positive urine glucose, difficulty urinating, decreased urine volume, polyuria, abnormal urinary sedimentation, cystitis, hematuria, rarely BUN, elevated serum creatinine and uric acid levels may occur.

J. Blood system: Leukocyte decrease, platelet decrease, red blood cell decrease, granulocyte decrease, hemoglobin and hematocrit decrease, anemia, lymphadenopathy, etc. may occur.

K. Autoimmune phenomena: Symptoms thought to be caused by autoimmune phenomena (hypothyroidism (hypothyroidism or hyperthyroidism), hepatitis, hemolytic anemia, exacerbation of ulcerative colitis, exacerbation of joint rheumatism, insulin-dependent diabetes mellitus (IDDM)) Deterioration or occurrence, etc.] may appear.

L. Respiratory system: fever, relief, shortness of breath, pneumonia and interstitial pneumonia with chest X-ray abnormalities, lung infiltration, upper respiratory tract infection, sore throat, rhinitis, nasopharyngitis, sinus congestion, pulmonary congestion,

chest tightness, exercise breathing Difficulty, sometimes pneumonia may occur, so if these symptoms appear, stop the exposure to exposure and take appropriate treatment, such as administering adrenocorticotropic hormone.

M. Pancreas: In rare cases, acute pancreatitis may occur. Observe enough. If abdominal pain or elevated serum amylase levels occur, stop administration and take appropriate treatment.

N. Cerebral hemorrhage: Cerebral hemorrhage (less than 0.1%) may occur. Observe closely and if abnormalities are found, stop administration and take appropriate treatment.

O. Musculoskeletal system: joint pain, muscle pain, bone pain, neck pain, back pain, muscle cramps, muscle weakness

P. Eyes: blurred vision, dry eyes, eye irritation, eye pain. Rarely, ocular abnormalities such as retinal hemorrhage, cotton nevus, visual neuropapillary edema, or obstruction of retinal arteries or veins are rarely reported after treatment with alpha-interferon. Patients who report decreased vision or loss of vision should have an eye exam. Because these types of signs can be associated with other disease states, it is recommended that patients with diabetes or hypertension have an ophthalmological examination prior to initiating treatment.

Q. Others: Sometimes weight loss, serum protein loss, rosacea pain, elevated blood sugar, rheumatoid arthritis, retinal hemorrhage, ischemic retinopathy, etc. may occur. In addition, since sepsis may occur, the patient's systemic condition should be sufficiently observed, and if abnormalities are recognized, administration should be discontinued and appropriate treatment should be taken. It has been reported that similar drugs (other interferon alpha agents) developed hemolytic uremic syndrome, which is the main symptoms of platelet reduction, hemolytic anemia, and renal failure.

R. Neutropenia and thrombocytopenia were more common with this drug than standard interferon. Less than 1% of patients with sclerosis required dose adjustment due to anemia. In about 4% of patients, the absolute neutrophil count temporarily dropped below 500/mm3 during treatment. The platelet count decreased to less than 50,000/㎣ was observed in about 5%, and most of them were patients with sclerosis or those with low platelet count of 75,000/㎣ at the time of participation in the test.

4) Drug interaction

A. The results of administering this drug 180㎍ once a week to healthy subjects for 4 weeks did not affect the pharmacokinetics of mephenytoin, dapsone, debrisoquin, or tolbutamide. Therefore, this drug does not affect the in vivo metabolic activity of the isoenzymes P450 3A4, 2C9, 2C19 or 2D6.

B. Concomitant administration of this drug increased the AUC of theophylline (a marker of cytochrome P450 1A2 activity) by 25%, confirming that this drug moderately inhibited the

action of cytochrome P450 1A2. Patients taking theophylline with this drug should monitor the serum concentration of theophylline and adjust the amount of theophylline accordingly. The maximal interaction of theophylline with this drug is expected to occur after 4 weeks of this drug administration.

C. Interferon has been observed to increase the neurotoxic, hematotoxic, and cardiotoxic effects of previously administered or combined drugs, so similar results are expected with this drug.

D. As with other interferon drugs, caution should be exercised when using this drug in combination with other myelosuppressive drugs.

E. No pharmacokinetic interactions were observed in the clinical trials of this drug and ribavirin.

F. Contraindications have not been tested, so this drug should not be mixed with other drugs.

G. Interstitial pneumonia was reported in a case of combination with Soshiho-tang, so it is not used in combination.

H. In a pharmacokinetic study of 24 HCV-infected patients who received methadone (average 95mg, 30~150mg) at the same time as a prophylactic regimen and then subcutaneously injected with this drug 180 μg weekly, the average methadone pharmacokinetic variable was 4 weeks. Compared with before and after administration of this drug, it was 10-15% higher after administration. The clinical significance of this study has not been well elucidated, but patients should be informed of any adverse reactions to methadone.

I. HIV-HCV double-infected patients: 47 patients who underwent a 12-week pharmacokinetic substudy to verify the effect of ribavirin on cellular phosphorylation of nucleic acid reverse transcriptase inhibitors (NRTIs, lamivudine, zidovudine, stavudine). There was no drug interaction in patients with HIV-HCV double infection. Plasma exposure of ribavirin was not shown to be affected by co-administration of NRTIs.

J. Co-administration of ribavirin and didanosine is not recommended. Exposure to didanosine or the active metabolite (dideoxyadenosine 5'-triphosphate) is increased when didanosine and ribavirin are administered in combination. Fatal liver failure has been reported along with peripheral neuropathy, pancreatitis, and symptomatic lactic acidemia due to ribavirin use.

K. The exact mechanism has not been identified, but exacerbation of ribavirin-induced anemia has been reported when zidovudine was administered to HIV as part of the treatment regimen. Co-administration of ribavirin and zidovudine is not recommended as it may increase the risk of anemia. Zidovudine replacement in ART combination therapy should be considered. Particular attention should be paid to patients with a history of anemia due to zidovudine.

L. In clinical trials, co-administration of telbivudine 600 mg once a day and 180 μg of this drug weekly was associated with an increased risk of peripheral neuropathy. The mechanism of action associated with this is unknown, but this risk cannot be ruled out for other interferons. In addition, the benefits of co-administration of telbivudine and interferon alpha (including peginterferon) have not been established at present.

M. Azathioprine: Ribavirin inhibits the metabolism of azathioprine by inhibiting inosine monophosphate dehydrogenase and can accumulate 6-methylthionosine monophoasphate (6-MTIMP), which was associated with bone marrow toxicity in patients treated with azathioprine. Combination of peginterferon alpha-2a with ribavirin and azathioprine should be avoided. If the benefits of co-administration ribavirin and azathioprine outweigh the potential risks, close hematologic monitoring is recommended during the co-administration of azathioprine to check for signs of myelotoxicity, and medication is recommended if such signs are identified. You have to stop.

5) Administration to pregnant and lactating women

A. Never used this drug in pregnant women. This drug should not be given during pregnancy.

3.9.2 **Lamivudine**

1) Do not administer to the following patients

A. Patients with hypersensitivity to lamivudine or other components of lamivudine

2) Carefully administer to the following patients

A. Reduction in dosage is recommended for patients with renal dysfunction.

B. Care should be taken when administering to children with a history of pancreatitis or at high risk of pancreatitis.

C. Observe carefully as hepatomegaly and lactic acidosis have been reported due to severe fatty liver.

D. Be aware that the risk of lactic acidosis and liver damage may increase due to pregnancy, obesity, and delay in treatment.

3) Side effect

A. >10%: Central nervous system (headache, fatigue), gastrointestinal system (nausea, diarrhea, vomiting, pancreatitis), nervous system and musculoskeletal system (peripheral neuritis, paresthesia, musculoskeletal pain)

B. 1-10%: central nervous system (dizziness, depression, fever, chills, insomnia), skin (rash), gastrointestinal system (anorexia, stomach abdominal pain, chest pain, increased amylase), hematology (neutropenia), liver (AST, ALT increase), nervous system and musculoskeletal system (muscle pain, joint pain), respiratory system (cough)

C. <1%: alopecia, anaphylaxis, anemia, hepatomegaly, hyperbilirubinemia, hyperglycemia, increased CPK, lactic acidosis, lymphadenopathy, peripheral neuropathy, pruritus, rhabdomyolysis, weakness, thrombocytopenia, gastritis

4) Drug interaction

A. When administered with zidovudine, the concentration of zidovudine can be increased by 39%.

B. Trimethoprim-sulfamethoxazole increases blood levels of this drug

C. When administered with Zalcitabine, avoid using it together as it inhibits the intracellular phosphorylation process and reduces the effect.

5) Administration to pregnant and lactating women

A. US (FDA) dispute: C, Australia (ADEC) classification: B3

B. Dosing during the first 3 months of pregnancy is not recommended.

3.9.3 **Entecavir**

1) Do not administer to the following patients

A. Patients with hypersensitivity reaction to entecavir or components of entecavir

2) Carefully administer to the following patients

A. Dose adjustment is recommended in patients with renal failure.

B. In liver transplant recipients receiving cyclosporine or tacrolimus, renal function should be carefully evaluated prior to and during treatment with this drug.

3) Side effect

A. >10%: liver (increased ALT)

B. 1-10%: Central nervous system (headache, fatigue), endocrine and metabolic system (hyperlipidemia), digestive system (increased lipase, increased amylase, diarrhea, indigestion)

C. <1%: dizziness, hypoalbuminemia, insomnia, nausea, drowsiness, thrombocytopenia, vomiting

4) Drug interaction

A. A. When co-administered with Ribavirin, liver failure or other signs of mitochondrial toxicity may be shown (pancreatitis, lactic acidosis, etc.)

B. B. Gancyclovir, valgancyclovir: adverse reactions, increased toxicity (eg, blood disorders)

5) Administration to pregnant and lactating women

A. During pregnancy, it should only be used if the benefit outweighs the risk to the fetus.

B. If you are taking this drug, you should avoid breastfeeding.

3.9.4 **Adefovir**

1) Do not administer to the following patients.

A. Patients with hypersensitivity to adefovir or other ingredients of this drug

2) Carefully administer to the following patients

Clinical evidence of lamivudine-resistant hepatitis B infection before (n=226) and after liver transplantation (n=24 chronic hepatitis B patients with an average treatment period of 51 weeks and 99 weeks, respectively, up to 203 weeks) For renal dysfunction, changes in renal function occurred in patients before and after liver transplantation with risk factors including cyclosporine and tacrolimus co-administration, initial renal insufficiency, hypertension, diabetes, and organ transplantation. Therefore, it is difficult to assess the effect of this drug on these changes in renal function, according to the Kaplan-Meier estimate, in 12% of patients by 48 weeks before liver transplantation, 28 by week 96. In% of patients, an elevation of ≥0.5 mg/dL of serum creatinine was observed in 30% of patients by 144 weeks from the initial stage, in 18% of patients by 48 weeks of liver transplantation and in 35% of patients by 96 weeks of liver transplantation , ≥0.5mg/dL of serum creatinine was increased by ≥0.5mg/dL from the initial stage in 35% of patients by 144 weeks. The Kaplan-Meier estimate for the percent (%) of patients who had undergone liver transplantation was higher after liver transplantation (52% vs 5%) than before liver transplantation, 1.3% of patients before liver transplantation (3 out of 226) until the last visit, liver. After transplantation, 2.5% of patients (6 out of 241) had a serum phosphorus level of <2.0 mg/dL. Of the patients before and after liver transplantation, 4% of patients (19 out of 467) had a renal response. In patients before and after liver transplantation treated with this drug, the most frequently reported adverse reactions with a frequency of 2% or higher are as follows.

A. Whole body: asthenia, headache, fever

B. Gastrointestinal tract: abdominal pain, nausea, vomiting, congestion of air in the gastrointestinal tract, diarrhea, liver failure, indigestion

C. Metabolism and nutrition: elevated ALT and AST, abnormal liver function, hypophosphatemia

D. Respiratory: increased cough, pharyngitis, sinusitis

E. Skin and skin appendages: itching, rash

F. Urinary system: elevated creatinine, kidney failure, lack of renal function부족

3) Side effect

A. >10%: asthenia

B. 1-10%: headache, abdominal pain, nausea, stomach, bloating of gas in the intestine, diarrhea, indigestion

C. <1%: Myopathy, osteomalacia, proximal ureteral disease, Fanconi syndrome, hypophosphatemia, pancreatitis

4) Drug interaction

A. Since adefovir is excreted by the kidneys, the serum concentration of adefovir or co-administered drugs can be increased when this drug is co-administered with drugs that reduce renal function or drugs that compete for the secretion of positive tubules.

B. Effects on renal function when combined administration of this drug with drugs excreted to the kidneys or drugs known to affect renal function, except for lamivudine, trimetoprim/sulfamethoxazole, acetaminophen, ibuprofen, and tenofovir Has not been studied.

C. This drug does not alter the pharmacokinetics of trimethoprim/sulfamethoxazole, acetaminophen, ibuprofen, and lamivudine.

D. Adverse reactions should be carefully monitored when co-administration of this drug with drugs excreted into the kidney or other drugs known to affect renal function.

E. When 800 milligrams of ibuprofen were administered three times a day, the AUC of adefovir increased by about 23% and the Cmax by about 33%. These increases are thought to be due to the higher bioavailability of this drug rather than a decrease in renal clearance, and the clinical significance of an increase in adefovir exposure is unknown.

F. Adefovir generally does not inhibit the CYP450 enzyme, but the possibility of adefovir inducing the CYP450 enzyme is not known.

G. The effect of adefovir on cyclosporine and tacrolimus concentrations is unknown.

H. Co-administration of this drug and lamivudine did not change the pharmacokinetics of each drug.

5) Administration to pregnant and lactating women

A. During pregnancy, it should only be used if the benefit outweighs the risk to the fetus.

B. If you are taking this drug, you should avoid breastfeeding.

3.9.5 **Clevudine**

1) Do not administer to the following patients

A. Patients with hypersensitivity to clevudine or other ingredients of this drug

B. Patients under the age of 18

C. Patients with renal dysfunction

D. Since this drug is mainly excreted through the kidneys, dose adjustment is necessary because clevudine excretion half-life may be prolonged due to decreased clearance in patients with renal impairment, but clinical studies on the dosage control of renal dysfunction patients have not been conducted. , Do not administer to patients with creatinine clearance less than 60mL/min.

2) Carefully administer to the next patient

A. Patients who need to be co-administered with other drugs

① This drug is excreted mainly to the kidneys, and concomitant administration with drugs that reduce renal function or compete for active tubular secretion can increase the serum concentration of these drugs. The effect of co-administration of this drug with other drugs that are excreted by the kidneys or known to affect renal function has not been evaluated. When this drug is administered in combination with these drugs, the patient should be closely monitored for adverse events.

② As a result of an in vitro test on the inhibition of the CYP450 enzyme system, which is a representative metabolic enzyme of the drug, it was confirmed that this drug does not act as an inhibitor for CYP450 1A2, 2C9, 2C19, 2D6, 3A4. Since it has not been studied, adverse reactions should be closely monitored when co-administered with drugs affected by this enzyme.

3) Adverse reaction

Adverse reactions were evaluated based on two therapeutically confirmed clinical trials in 330 chronic hepatitis B patients treated with this drug (n=246) or placebo (n=84) for 4 weeks. In these clinical studies, about 2.4% of patients discontinued the study due to adverse reactions or abnormal clinical test values in the placebo-treated group, and none in the patient group receiving this drug.

A. Clinical adverse reaction

Over 5% of reported adverse reactions during treatment with this drug (30 mg of clevudine once daily for 24 weeks) were infection (cold or upper respiratory tract infection), weakness, abdominal pain, indigestion, and headache. Moderate to severe clinical trials that may be related to this drug. Adverse reactions were cold or upper respiratory tract infection, rash, drowsiness, and headache, and the incidence rate was less than 1% in all of the clevudine-administered groups. The table below shows moderate-to-severe (Grade 2-4) clinical adverse reactions reported during the 24-week dosing period in a therapeutically confirmed clinical trial.

B. Clinical test value or higher

In two therapeutically confirmed clinical trials comparing this drug versus placebo, ALT, ALT, Creatinin phosphokinase, and lipase were increased by 2-5 times the upper limit of normal during treatment. In these studies, among patients treated with this drug, elevations in ALT or AST at the beginning of treatment were generally lost as treatment continued with this drug.

Periodic liver function tests are also recommended during treatment.

C. hepatitis worsens

In clinical trials that confirm the treatment of this drug, ALT or AST was defined as "exacerbation of hepatitis" when the ALT or AST level exceeded >20×ULN or >10×ULN and 10 times the baseline value. During the treatment period and during the follow-up period after treatment discontinuation, ‘exacerbation of hepatitis’ was observed in 2-5.5% in the clevudine group and 4.9-13% in the placebo group.

4) Side effects (when administered for 24 weeks)

A. >10%: infection (cold or upper respiratory tract infection), weakness, increased liver enzyme levels

B. 1-10%: abdominal pain, indigestion, headache, worsening hepatitis

C. <1%: fracture, rash, drowsiness, cellulitis, conjunctivitis, endometritis, gingivitis, gastric ulcer

5) General caution

A. The safety and efficacy of this drug is based on the results of a placebo-controlled therapeutic confirmation clinical trial in which 30 mg of this drug was administered once a day for 24 weeks, and no clinical trials comparing the active drug as a control have been studied.

B. The results of the therapeutic confirmation clinical trial showed statistically significant differences compared to the placebo group in terms of the amount of reduction in serum HBV DNA compared to the baseline point, the ratio of subjects below the detection limit of HBV DNA, and the normalization rate of ALT compared to the placebo group. There was no statistically significant difference in conversion rates in the this drug-treated group compared to the placebo group, and histological changes through liver biopsy were not evaluated.

C. The correlation between the optimal treatment duration and treatment and long-term results (cirrhosis and hepatocellular carcinoma) of this drug in chronic hepatitis B patients is not known, and patients with advanced cirrhosis carefully monitor the progression of cirrhosis. Should be.

D. Patients should be informed that treatment with this drug has not been shown to reduce the risk of HBV transmission due to sexual intercourse or blood contamination and therefore appropriate caution should be exercised.

E. The therapeutic effect of clevudine against the nucleoside resistant mutant virus

Ⓐ in vitro effect

Clvudine showed an effect on HBV containing the sole mutations M550V, V519L, M553I, and A546V, but not M550I. The effect of clevudine on viruses containing the sole mutation, L526M, was not constant. Clevudine was not active in HBV containing multiple mutations L526M/M550I or L526M/M550V.

Ⓑ Clinical effect

① Clevudine for patients who have been treated with lamivudine, a small number (n=7) of the nucleoside analogue, and have genetic evidence for the YMDD mutation (M550I/V) of HBV DNA polymerase.

② As a result of a clinical trial evaluating the antiviral activity of HBV, there was no significant inhibitory effect when evaluated in terms of HBV DNA change compared to the baseline.

i. As a result of the clinical trial of clevudine, L526M and M550I/V, which are lamivudine resistance mutations, were not found in the clevudine group during the scheduled administration period. Some changes have been reported. The consistency of these changes and the rebound of HBV DNA levels were not correlated, but the results of studies on the clevudine resistance genotype of HBV are limited.

F. The clinical effect of this drug on hepatitis C and D is not known.

G. There is no clinical experience with this drug in patients with decompensated liver disease or organ transplants, HCV, HDV, or concurrently infected with HIV and HBV.

H. There is no clinical experience with this drug in patients receiving immunosuppressive drugs, including cancer chemotherapy drugs.

I. The effect of this drug on the ability to drive or operate machinery has not been studied, and impairment of these capabilities cannot be predicted from the pharmacological action of this drug. Nevertheless, when considering a patient's ability to operate or operate machinery, the clinical condition of the patient and adverse reaction aspects of this drug should be kept in mind.

J. Cases of myopathy were reported when using this drug in the postmarketing side effects investigation. Myopathy has also been reported with other drugs in this class. In patients treated with clevudine, muscle pain without complications has been reported. Myopathy should be considered in patients complaining of unexplained diffuse muscle pain, muscle tenderness, and muscle weakness. Myopathy is defined as muscle pain and/or weakness of muscle whose cause is persistently unknown, regardless of the degree of elevation of creatinine kinase (CK). There are no patterns related to the degree or time of CK levels among patients with myopathy related to clevudine. In addition, the predisposition to developing myopathy in patients treated with clevudine was not known. Patients must be

informed of persistent unknown muscle pain, muscle tenderness, and muscle history. And if myopathy is diagnosed, clevudine should be stopped. The causal relationship between administration of this class of drugs and whether the increased risk of myopathy is due to coadministration with other drugs associated with myopathy is unknown. Doctors should carefully consider the potential benefits and risks of taking other drugs associated with myopathy with this drug, and monitoring for symptoms or signs of unknown muscle pain, muscle tenderness, and myasthenia.

K. HBV DNA level is less than 4,700 copies/mL, ALT level is normal, HBeAg positive patient at the time of follow-up for 3 months or 6 months in patients who took clevudine for 6 months or 1 year or 1 year and 6 months Patients who showed HBeAg seroconversion continuously at the last 2 time points were followed up for an additional 2 years. Among 63 patients enrolled in this clinical trial (HBeAg-positive: 26, HBeAg-negative: 37), patients with HBV DNA levels of less than 4,700 copies/mL after 2 years of follow-up were: It was 35% (HBeAg positive patients: 46%, HBeAg negative patients: 27%), and 73% of patients with HBV DNA levels less than 141,500 copies/mL (HBeAg positive patients: 77%, HBeAg negative patients: 70%). And 75% of patients with normal ALT levels (HBeAg positive patients: 88%, HBeAg negative patients: 65%) and HBeAg seroconversion was maintained at 81%.

6) Interaction

A. Interaction with food : As a result of the pharmacokinetic evaluation of this drug (Clevudine 600mg) after fasting and high fat diet, it was found that Cmax decreased while Tmax increased and the absorption rate decreased, but the dispersion coefficient for AUC, CL/F, and Cmax. Considering the bioavailability was found to be constant.

B. Interaction with drugs

① Since this drug is mainly excreted by the kidneys, coadministration with drugs that reduce renal function or compete for active tubular secretion can increase the serum concentration of these drugs. The effect of co-administration of this drug with other drugs known to be excreted by the kidney or affect renal function was not evaluated. When this drug is administered in combination with these drugs, the patient should be closely monitored for adverse reactions.

② As a result of an in vitro test on the inhibition of the CYP450 enzyme system, which is a representative metabolic enzyme of the drug, it was confirmed that this drug does not act as an inhibitor for CYP450 1A2, 2C9, 2C19, 2D6, 3A4. Since it has not been studied, adverse reactions should be closely monitored when co-administered with drugs affected by this enzyme.

7) Administration to pregnant and lactating women, children and elderly patients, etc.

A. pregnant women : Reproductive toxicity studies in rats and rabbits showed no evidence of teratogenicity, no effect on male and female fertility or fetus. In a reproductive toxicity study in which clevudine was administered orally to rats up to 1000 mg/kg per day and rabbits up to 500 mg/kg per day, there were no external malformations or developmental mutations in fetuses due to administration. Except for reproductive toxicity studies in animals, no studies have been conducted on pregnant women. This drug should be used during pregnancy only if the benefits outweigh the risk to the fetus. There are no data on the effect of this drug on the transmission of HBV from mother to infant.

B. Nursing mothers: It is not known whether this drug is secreted into human milk, so if you take this drug, you should avoid breastfeeding.

C. Children: There is no clinical experience with this drug in patients under 18 years of age.

D. Elderly people: Subjects 65 years of age or older do not participate in clinical studies of this drug, so it cannot be confirmed whether they respond differently from those of young adults. A significant amount of this drug is excreted by the kidneys, and renal function in elderly patients will be further reduced, so care should be taken in dose selection and it is useful to monitor renal function during the administration period.

3.9.6 **Tenofovir**

1) Do not administer to the following patients

A. Patients with hypersensitivity to this drug or other ingredients contained in this drug

B. Since this drug contains lactose, it is administered to patients with genetic problems such as galactose intolerance, Lapp lactase deficiency, or glucose-galactose malabsorption.

2) Adverse reactions in clinical trials

Since clinical trials are conducted under a wide variety of conditions, the rates of adverse reactions observed in clinical trials of drugs cannot be directly compared with rates of adverse reactions observed in clinical trials of other drugs, and may not be reflected in the rates observed in practice.

A. Clinical trial of chronic hepatitis B patients

① Clinical trial on adult patients with chronic hepatitis B and target liver disease : Treatment-related adverse reactions: In controlled clinical trials in patients with chronic hepatitis B (0102 and 0103), more patients treated with this drug experienced nausea during the 48-week double-blind period (9% YELLOW vs Adefovir Difficile). Replicant formulation 2%). Other treatment-related adverse reactions reported by more than 5% of patients treated with this drug include abdominal pain, diarrhea, headache, dizziness, fatigue, nasopharyngitis, low back pain, and skin rash. In this trial, no significant

changes in the tolerability profile (characteristic or severity of adverse reactions) were observed in patients receiving continuous treatment with this drug for up to 144 weeks.

② Clinical trial on adult subjects suffering from chronic hepatitis B and noncompensated liver disease : In a small, randomized, double-blind, active-controlled trial (0108), patients with CHB and decompensated liver disease were treated with this drug or other antiviral drugs for up to 48 weeks. The most frequently reported treatment-related adverse reactions in 45 patients receiving this drug treatment were abdominal pain (22%), nausea (20%), insomnia (18%), itching (16%), vomiting (13%), and dizziness (13%) and fever (11%). Two of the 45 patients (4%) died during the 48-week trial period due to progression of epilepsy. Of the 45 patients, 3 (7%) discontinued treatment due to adverse reactions. Four of the 45 patients (9%) had a 0.5 mg/dL increase in serum creatine (and one had a serum phosphate of less than 2 mg/dL over 48 weeks). Three of these patients (early with a Child-Pugh index of 10 or less and a MELD index of 14 or more) had worse kidney damage. It is difficult to determine the extent to which this drug had an effect on kidney damage in this population, as both this drug and decompensated liver disease can affect kidney function. One in 45 subjects suffered from hepatitis flares during treatment during the 48-week trial period.

B. Side effects (when administered for 48 weeks)

① >10%: asthenia, pain, diarrhea, nausea

② 1-10%: headache, abdominal pain, back pain, chest pain, fever, vomiting, loss of appetite, indigestion, bloating, pneumonia, depression, insomnia, speech

③ Super neuropathy, dizziness, rash reaction, sweating, muscle pain, weight loss

C. Post-marketing investigation

The following adverse reactions were observed during use after Tenofovir was approved. Since postmarketing adverse reactions are reported spontaneously from a population of uncertain size, they do not always reliably predict their incidence or demonstrate a causal relationship to drug administration.

① Immune system disorder: Allergic reaction (including angioedema)

② Metabolic and nutritional disorders: lactic acidosis, hypokalemia, hypophosphatemia

③ Respiratory system, chest and mediastinal disorders: Difficulty breathing

④ Gastrointestinal disorders: pancreatitis, increased amylase, abdominal pain

⑤ Hepatic gallbladder disorder: hepatic steatosis, hepatitis, increased liver enzymes (most commonly AST and ALT gamma GT)

⑥ Skin and subcutaneous tissue disorders: rash

⑦ Musculoskeletal and connective tissue disorders: rhabdomyolysis, osteomalacia (represented as bone pain and may cause fracture), muscle weakness, muscle disease

⑧ Renal and urinary disorders: acute renal failure, renal failure, acute tubular necrosis, Fanconi syndrome, proximal tubule disease, interstitial nephritis (including acute cases), renal diabetes insipidus, renal failure, increased creatinine, proteinuria, polyuria

⑨ General disorders and conditions at the site of administration: asthenia

⑩ In the adverse reactions listed above, rhabdomyolysis, osteomalacia, hypokalemia, muscle weakness, muscle disease, and hypophosphatemia may occur as a result of proximal renal tubulopathy.

D. Drugs that affect kidney function

Because tenofovir is excreted in the kidneys in principle, when this drug is administered together with a drug that decreases kidney function or competes for active tubular secretion, it increases the serum concentration of tenofovir or the concentration of other drugs that are eliminated from the kidney. Can increase. Although not necessarily limited thereto, some examples include cidofovir, acyclovir, gansicrovir, balacyclovir, and valganciclovir. Drugs that weaken kidney function can also increase the serum concentration of tenofovir.

3.10 Criteria for Suspension or Dropout of Clinical Research

3.10.1 Criteria for dropout

1) In case the subject withdraws consent to participate in this test

2) In case of serious side effects

3.10.2 Criteria for Ending the Trial

1) After randomization, Peginterferon α2a was administered for 48 weeks.

3.11 Effectiveness evaluation criteria, evaluation method and interpretation method (statistical analysis method)

3.11.1 Criteria for evaluating effectiveness

1) Primary endpoint: Changes in HBsAg quantity (log10 HBsAg) during administration of antiviral agents in each group

2) Secondary endpoint

1. Comparison of changes from baseline in serum HBV DNA levels and HBV DNA non-detection

rates and HBV DNA <20 IU/mL during administration of antiviral agents in each group and follow-up

i. Comparison of the ratio of HBV DNA <2,000 IU/mL during follow-up and administration of antiviral agents in each group

ii. Comparison of the ratio of HBV DNA <20,000 IU/mL during the administration of antiviral agents in each group and follow-up

2. Comparison of HBe-antigen seroconversion rate and loss rate during the administration of antiviral agents and follow-up of each group

3. Comparison of HBs-antigen seroconversion rate and loss rate at 1 and 2 years after administration/termination of antiviral agents in each group

4. Changes in the quantity of HBsAg (log10 HBsAg) after administration/termination of antiviral agents in each group

3.11.2 Statistical analysis method

The primary outcome variable, HBsAg quantification (log10 HBsAg), is an index predicting seroconversion in patients with HBe-antigen chronic hepatitis B infection. This clinical study is a superiority clinical trial to evaluate that the effective rate of the test drug (Peginterferon α2a 180 ug/mL) is superior to that of the reference drug (Lamivudine 100 mg, Entecavir 0.5 mg, Adefovir 10 mg). . The log10 HBsAg by each treatment effect is compared using independent t test. The analysis of secondary outcome variables such as the HBe-antigen seroconversion and loss rate, and the HBs-antigen seroconversion and loss rate is analyzed using logistic regression. Effectiveness and stability analysis for this clinical trial is based on Intent-to-treat (ITT) analysis.

3.12 Safety evaluation criteria including side effects, evaluation methods and reporting methods

3.12.1 Definition of adverse events

“Adverse Event (AE)” refers to undesirable and unintended signs (signs, eg, abnormalities in laboratory test values), symptoms, and diseases that occurred in a subject receiving a drug used in a clinical trial. It does not necessarily have a causal relationship with the drug used in the clinical trial. Therefore, the study investigator instructs the patient to report any adverse reactions. This definition includes concomitant disease or injury, or exacerbation of an existing condition. Unexpected adverse reactions are adverse reactions for which the nature, severity, or frequency of drugs used in currently approved clinical trials is not identified.

All adverse reactions occurring during the clinical trial period, even if they are not related to the administration of the investigational drug, should be recorded in the adverse reactions column of the subject's medical record and case record according to the investigator's normal medical practice.

Investigators should make a diagnosis of adverse events based on signs, symptoms and/or other clinical information. In this case, the diagnosis name, not individual signs or symptoms, is documented and recorded as an adverse reaction. When a clear diagnosis is made, individual signs and symptoms do not need to be reported as separate events unless they are atypical or overexpressed. In the absence of a clear diagnosis, all individual signs and symptoms should be recorded separately.

If a clinically important laboratory finding or other abnormal measurement is an adverse reaction, it should be entered in the adverse reaction column of the case report.

All adverse events should be followed up until they are resolved, the condition stabilizes, the adverse events can be explained, or no further follow-up is possible.

If follow-up of the subject was not possible before the investigator confirmed that the adverse reaction was resolved or stabilized or the cause could be explained, the clinical trial agency continued to contact the subject for information (at least two times). The record should be documented in the general Comment column of the subject's medical record and case record. These records should include the date and means of communication.

3.12.2 Severity evaluation criteria

Among the adverse reactions that occurred, adverse reactions that occurred in diagnostic medical tests are classified according to the WHO adverse reaction criteria, and adverse reactions not included in this are classified using a three-stage classification method such as Spiker.

Severity indication	Contents
**Mild**	When treatment is not required and the subject's normal life (function) is not significantly impaired
**Moderate**	Significantly impeding the subject's normal life (function), Treatment may be required and recovery after treatment
**Severe**	Severe adverse reactions require high-level treatment, leaving sequelae or threatening life

3.12.3 Definition and reporting criteria for serious adverse reactions / adverse drug reactions

3.12.3.1 Definition

"Serious adverse reactions/adverse drug reactions (Serious AE/ADR)" refers to cases falling under any of the following items among adverse reactions or adverse drug reactions occurring at any dose of a drug used in clinical trials.

A. Death

B. Life-threatening; It refers to a case in which it is judged that the subject may be subject to immediate death at the time of the occurrence of the event, and adverse reactions that may have resulted in death if developed into a more serious condition are not regarded as serious.

C. In case hospitalization or extension of hospital stay is necessary: For medical reasons, hospitalization is required for continuous observation. Hospitalization refers to the case of official admission to the hospital for medical reasons, and includes only those cases in which hospitalization is required medically among the visits to the emergency room. In addition, it is not included in the case of hospitalization due to the subject's personal reason and optional doctor.

D. In case of disability or functional deterioration

E. If it causes birth defects or abnormalities

F. Exacerbation of hepatitis B is included as a serious adverse event when medical or surgical treatment is required, even if it is not included in the above criteria for severity.

3.12.3.2 **Reporting Criteria**

All serious adverse reactions, regardless of their relevance to the investigational drug, must be reported to the Institutional Review Board and Roche Korea (tel 02 3451 3844, fax 02 557 7201) within one working hour from the time point recognized by the investigator. The initial report should be as complete as possible, including an assessment of the current disease and serious adverse events, as well as the causal relationship between the adverse events and the study drug.

SUSAR reporting

1) In case of death or life-threatening: Within 7 days from the date the lead researcher reported or became aware of this fact. However, in this case, detailed information is additionally reported within 8 days from the date of the initial report.

2) All other serious and unexpected adverse drug reactions: within 15 days from the date the lead investigator reported or became aware of this fact

All subjects with serious adverse reactions must follow up the results, and the responsible investigator periodically terminates the adverse drug reaction with additional safety information in connection with the above report (disappearance of the adverse drug reaction or impossibility of follow-up, etc.) Must be reported until it becomes available.

3.12.4 **Criteria for determining causal relationship**

3.12.4.1 **Whether it is related to test drug administration**

The degree of association between test drug administration and the occurrence of adverse reactions is determined by the investigator. However, the following criteria are reference criteria prepared based on currently available information to help the investigator measure the degree of association between the administration of the test drug and the occurrence of adverse reactions. The greater the relationship between each item and its components (in terms of frequency or intensity), the higher the relationship between test drug administration and adverse reactions.

1.Exposure

Is there any evidence that the subject actually received the drug? (E.g., reliable drug use, acceptable compliance measurements, expected pharmacological effects, in vivo drug/metabolite

measurements, etc.)

2. Time Course

In the event of an adverse reaction, did it appear in an appropriate temporal sequence after administration of the test drug?

Does the time of occurrence of the adverse reaction match the effect of administration of the test drug well?

3. Likely Cause

Is the test drug more reasonable as a cause for explaining adverse events than other etiologies, such as the subject's underlying disease, other drugs, or other host-environment factors?

4. Dechallenge

Are adverse reactions eliminated or ameliorated by discontinuing or reducing the dose of the test drug? (Note: This item is not applicable if the adverse reaction results in death or permanent disability, or if the adverse reaction disappears or improves despite continued use of the test drug.)

5. Rechallenge

Does the adverse reaction recur or worsen by repeated administration of the test drug, or does the subject have a history of causing a similar adverse reaction to the same test drug or similar class of drugs to the study drug? (Note: This item is not applicable when an adverse reaction results in death or permanent disability)

6. Consistency of test drug profile

Are the clinical/pathological manifestations of adverse events consistent with existing knowledge of the pharmacology or toxicology of drugs of an equivalent or similar class?

1.Definitely related

1) If there is evidence that the test drug was administered

2) When the temporal sequence of administration of test drug and occurrence of adverse reactions is reasonable

3) When the adverse reaction is most likely explained by the administration of the test drug than for any other reason

4) When the adverse reaction disappears due to discontinuation of administration

5) If the result of re-administration (rechallenge, conducted only if possible) is positive

6) When an adverse reaction shows a pattern consistent with previously known information about the test drug or test drug of the same series.

2. Probably related

1) If there is evidence that the test drug was administered

2) When the temporal sequence of administration of test drug and occurrence of adverse reactions

is reasonable

3) When the adverse reaction is more likely explained by the administration of the test drug than by other causes

4) When the adverse reaction disappears due to discontinuation of administration

3. Possibly related

1) If there is evidence that the test drug was administered

2) When the temporal sequence of administration of test drug and occurrence of adverse reactions is reasonable

3) When it is judged that the adverse reaction is caused by the test drug at the same level as other possible causes

4) When the adverse reaction disappears due to discontinuation of administration (if carried out)

4. Probably not related

1) If there is evidence that the test drug was administered

2) If there is a more probable cause for an adverse reaction

3) If the result of discontinuation of administration (if implemented) is negative or ambiguous

4) Re-administration (if implemented) If the result is negative or ambiguous

5. Definitely not related

1) When the subject/patient has not been administered the test drug, or

2) When the temporal sequence between administration of the test drug and the onset of adverse reactions is not valid, or

3) If there is another obvious cause for the adverse reaction

If it is more than "possibly related", it is judged that there is a causal relationship with the test drug.

3.12.5 Reporting safety information

The investigator must immediately report any unexpected problems that pose a subject risk to the appropriate clinical trial review committee or ethics committee. This includes death of any cause and any serious adverse reactions associated with or suspected of being associated with test drug administration. Regardless of the causal relationship, all serious adverse reactions should be reported to the Institutional Review Board, and a copy should be kept in the researcher's file at the clinical trial agency.

3.13 Interim analysis and clinical trial protocol change

3.13.1 Interim analysis

Interim analysis is conducted 24 weeks after the initiation of the clinical trial.

3.13.2 Protocol plan change

If the researcher determines that a change in the planned treatment method is necessary during the clinical trial, the treatment plan should not be arbitrarily changed, and the plan must be changed according to the change planning procedure, and the modified plan is reviewed by the Clinical Trial Review Committee It should be applied.

3.14 Monitoring of clinical trial

In order to comply with the clinical trial management criteria, the monitoring procedure is performed by the designated monitor. Direct access to clinical trial records and medical records must be guaranteed at the clinical trial agency. Monitoring is performed through a visit to a monitor designated by the investigator in order to check the completeness and accuracy of the clinical trial case record and to determine the consistency with the supporting documents. In addition to monitoring visits, frequent communication (letter, telephone, or fax) with the clinical trial monitor allows the trial to be conducted in accordance with the design of the protocol and relevant regulations. The clinical trial termination procedure is performed by the clinical trial monitor at the end of the clinical trial.

3.15 Consent and protective measures for the subject

The investigator must fully explain the clinical trial to the subject and provide sufficient time before participation as to whether or not each subject will participate in the study, and then must obtain documented consent for voluntary participation from the subject. Consent is documented by signing and dated by the subject on the consent form.

If the subject is a legally incompetent person (e.g., mentally retarded, limited or incompetent, etc.), written consent must be obtained from a parent, legal guardian, or legal representative.

Despite the fact that the investigator complies with the relevant laws and regulations and conducts the clinical trial strictly according to various related documents, recommendations, and suggestions, the subject suffers from the occurrence of adverse reactions that are causally related to the test drug used in this clinical trial. If so, the investigator should endeavor to apply the appropriate compensation rules in accordance with the victim's compensation protocol.

3.16 Measures for the safety protection of subjects

Institutions conducting clinical trials shall equip the necessary facilities and professional manpower for

clinical trials and make every effort to protect safety so that the clinical trials can proceed properly as specified in this trial protocol.

The person in charge of the clinical trial should be fully aware of the adverse reactions and cautions specified in this plan in advance, and if a serious adverse reaction occurs during the study, immediately stop the clinical trial of the patient and take appropriate measures, and then the clinical trial review committee and the test. You must notify the person in charge.

3.17 **Research Execution Plan Table**

Study procedure	**Screening**	**Baseline**	**On peg IFN Rx**<br>**Weeks 4, 8, 12**<br>**Months 6, 9, 12**	**Post IFN Rx**<br>**6, 12, 24**<br>**months**
Visit window	-30	0	±14	±14
Subject consent	x			
Basic information investigation	x			
Review of selection/exclusion criteria	x	x		
Medical history	x			
General blood test	x	x	SOC	SOC
General chemical test, PT-time	x	x	SOC	SOC
HBsAg (guatitation)/ Ab	x		x	x
Basic ophthalmology examination	x			
Serum HBV DNA	x	x	SOC	SOC
HBeAg / Ab	x	x	SOC	SOC
HBV genotype	x			
TSH	x		SOC	SOC
Alpha-fetoprotein	x		**SOC**	SOC
Innate immunity study	x		**TBA**	**TBA**
Abdominal ultrasound (or imaging tests such as CT, MRI, etc.)	x		SOC	SOC
Randomization		x		
Research drug prescription		x	x	
Adverse events			x	
Concomitant drugs	x	x	x	
Confirmation of worsening disease			x	x

1. HBV DNA, HBeAg/Ab tests within 3 months, Hematology lab, Serum chemistry within 1 month are accepted as screening and baseline tests. (The screening test can be cited as the baseline test.)
2. Alpha-fetoprotein and imaging tests are recognized as screening tests within 3 months.
3. Hematological test-WBC, RBC, Hemoglobin, Platelet count.
4. General chemical test-ALT, AST, ALK-P, T-Bil., Alb/Total Protein.Ca/I-P, Glu, BUN/Cr, Uric acid, Cholesterol.
5. HBeAg/Ab check every 6 months at the start of treatment, and if seroconversion is confirmed twice in a row, it can be stopped at the discretion of the investigator.
6. Other test items are conducted under the judgment of the researcher.
7. SOC, standard of care

3.18 References

1. Ahn YO. Screening target groups for hepatitis B virus infection. In Zuckerman A. ed. Hepatitis B in the Asian-Pacific Region, vol. 1. Screening, Diagnosis and Control. London: Royal College of Physicians of London, 1997;13-19.
2. Chen, C.J., et al., Risk of hepatocellular carcinoma across a biological gradient of serum hepatitis B virus DNA level. JAMA, 2006. 295(1): p. 65-73.
3. Iloeje, U.H., et al., Predicting cirrhosis risk based on the level of circulating hepatitis B viral load. Gastroenterology, 2006. 130(3): p. 678-86.
4. Yang, H.I., et al., Hepatitis B e antigen and the risk of hepatocellular carcinoma. N Engl J Med, 2002. 347(3): p. 168-74.
5. Hsu, Y.S., et al., Long-term outcome after spontaneous HBeAg seroconversion in patients with chronic hepatitis B. Hepatology, 2002. 35(6): p. 1522-7.
6. Chu, C.M., et al., Natural history of hepatitis B e antigen to antibody seroconversion in patients with normal serum aminotransferase levels. Am J Med, 2004. 116(12): p. 829-34.
7. Lin, S.M., et al., Interferon therapy in HBeAg positive chronic hepatitis reduces progression to cirrhosis and hepatocellular carcinoma. J Hepatol, 2007. 46(1): p. 45-52.
8. Niederau, C., et al., Long-term follow-up of HBeAg-positive patients treated with interferon alfa for chronic hepatitis B. N Engl J Med, 1996. 334(22): p. 1422-7.
9. Mommeja-Marin, H., et al., Serum HBV DNA as a marker of efficacy during therapy for chronic HBV infection: analysis and review of the literature. Hepatology, 2003. 37(6): p. 1309-19.
10. Schiff, E.R., et al., Long-term treatment with entecavir induces reversal of advanced fibrosis or cirrhosis in patients with chronic hepatitis B. Clin Gastroenterol Hepatol, 2011. 9(3): p. 274-276 e1.
11. Yuan, H.J., et al., The relationship between HBV-DNA levels and cirrhosis-related complications in Chinese with chronic hepatitis B. J Viral Hepat, 2005. 12(4): p. 373-9.
12. Hsu, Y.S., et al., Long-term outcome after spontaneous HBeAg seroconversion in patients with chronic hepatitis B. Hepatology, 2002. 35(6): p. 1522-7.
13. Chu, C.M. and Y.F. Liaw, HBsAg seroclearance in asymptomatic carriers of high endemic

areas: appreciably high rates during a long-term follow-up. Hepatology, 2007. 45(5): p. 1187-92.

14. Liu, J., et al., Incidence and determinants of spontaneous hepatitis B surface antigen seroclearance: a community-based follow-up study. Gastroenterology, 2010. 139(2): p. 474-82.
15. Liaw, Y.F., M.R. Brunetto, and S. Hadziyannis, The natural history of chronic HBV infection and geographical differences. Antivir Ther, 2010. 15 Suppl 3: p. 25-33.
16. Fattovich, G., et al., Delayed clearance of serum HBsAg in compensated cirrhosis B: relation to interferon alpha therapy and disease prognosis. European Concerted Action on Viral Hepatitis (EUROHEP). Am J Gastroenterol, 1998. 93(6): p. 896-900.
17. van Zonneveld, M., et al., Long-term follow-up of alpha-interferon treatment of patients with chronic hepatitis B. Hepatology, 2004. 39(3): p. 804-10.
18. Lampertico, P., et al., Long-term suppression of hepatitis B e antigen-negative chronic hepatitis B by 24-month interferon therapy. Hepatology, 2003. 37(4): p. 756-63.
19. Keeffe, E.B., et al., A treatment algorithm for the management of chronic hepatitis B virus infection in the United States: 2008 update. Clin Gastroenterol Hepatol, 2008. 6(12): p. 1315-41; quiz 1286.
20. Gish, R.G., et al., Entecavir therapy for up to 96 weeks in patients with HBeAg-positive chronic hepatitis B. Gastroenterology, 2007. 133(5): p. 1437-44.
21. Jung, Y.K., et al., Change in serum hepatitis B surface antigen level and its clinical significance in treatment-naive, hepatitis B e antigen-positive patients receiving entecavir. J Clin Gastroenterol, 2010. 44(9): p. 653-7.
22. Brunetto, M.R., et al., Hepatitis B virus surface antigen levels: a guide to sustained response to peginterferon alfa-2a in HBeAg-negative chronic hepatitis B. Hepatology, 2009. 49(4): p. 1141-50.
23. Hadziyannis, S.J., et al., Long-term therapy with adefovir dipivoxil for HBeAg-negative chronic hepatitis B. N Engl J Med, 2005. 352(26): p. 2673-81.
24. Liu, F., et al., Poor durability of lamivudine effectiveness despite stringent cessation criteria: A prospective clinical study in hepatitis B e antigen-negative chronic hepatitis B patients. J Gastroenterol Hepatol, 2011. 26(3): p. 456-60.
25. Fung, J., et al., The duration of lamivudine therapy for chronic hepatitis B: cessation vs. continuation of treatment after HBeAg seroconversion. Am J Gastroenterol, 2009. 104(8): p. 1940-6; quiz 1947.
26. Lee, H.W., et al., Lamivudine maintenance beyond one year after HBeAg seroconversion is a major factor for sustained virologic response in HBeAg-positive chronic hepatitis B. Hepatology, 2010. 51(2): p. 415-21.
27. Chang, T.T., et al., Entecavir treatment for up to 5 years in patients with hepatitis B e antigen-positive chronic hepatitis B. Hepatology, 2010. 51(2): p. 422-30.

28. Leung, N.W., et al., Extended lamivudine treatment in patients with chronic hepatitis B enhances hepatitis B e antigen seroconversion rates: results after 3 years of therapy. Hepatology, 2001. 33(6): p. 1527-32.

4. Subject explanation and consent

PATIENT INFORMATION SHEET
Version 2.0; October 2016

Subject Explanation and Consent

This consent form is designed to provide you with information about this clinical study. The decision to participate in this study is up to you. In all matters, you are free to decide to participate or give up. In addition, you will not receive any disadvantage for your decision. If you wish to participate in the clinical study after reading the description below, the clinical study will be conducted only if you have voluntarily consented to the signature.

1. Research Title: A Study on the Quantitative Changes of Hepatitis B Surface Antigens and the Relationship between HBe-antigen Seroconversion in 48-week Administration of Peginterferon in HBe-antigen-positive Chronic Hepatitis B Patients Under Long-Term Nucleotide Maintenance Therapy

2. Investigator and implementation organization

: Jeong Heo

Pusan National University Hospital

Address : 179, Gudeok-ro, Seo-gu, Busan, 49241

3. Purpose of Clinical Research: To evaluate the relationship between the quantitative change of HBs-antigen and HBe-antigen seroconversion in patients with HBe-antigen-positive chronic hepatitis B who are undergoing long-term nucleotide maintenance therapy.

Chronic hepatitis B is a serious global health problem and is the leading cause of chronic liver disease, cirrhosis and primary liver cancer. Since the introduction of lamivudine, an oral antiviral agent that is easy to administer and has few side effects in 1998, several antiviral drugs have been newly developed. They are known to inhibit viral replication, normalize liver enzyme levels, and improve histological findings. It is not yet determined how long treatment should be continued after the growth of the virus has been suppressed using oral antiviral drugs. HBe antigen-negative hepatitis mostly recurs after treatment is terminated, and in HBe antigen-positive hepatitis, if the treatment is continued after HBe antigen seroconversion, the persistence of inhibition of viral proliferation increases. Hepatitis B virus DNA remains HBeAg positive for several years even if the hepatitis B virus DNA is measured below the sensitivity of the hepatitis B virus DNA PCR conducted in hospitals, and in this case, most of them revert to the state before treatment when the oral antiviral drug is stopped.

Recently, a lot of research on surface antigens is being conducted as an alternative indicator for treatment evaluation. In the natural course of hepatitis B, the rate of serum loss of surface antigens occurs about 1~2% per year, and the likelihood of liver-related complications such as cirrhosis and hepatocellular carcinoma decreases due to the decrease in hepatitis B virus DNA. In the past, the surface antigen serum loss rate was high in patients whose viral proliferation was suppressed through interferon alpha treatment, and liver-related mortality such as liver function loss and hepatocellular carcinoma were decreased in patients with surface antigen serum loss, but the surface loss indicating ultimate cure. The rate of antigen loss is very low.

The serum loss rate of surface antigens is higher in the interferon treatment group than in the oral antiviral treatment group, but if the period of use of the oral antiviral agent is prolonged, the loss rate of the surface antigen may be increased as much as the interferon treatment. There is a correlation between the decrease in the surface antigen titer and the decrease in the hepatitis B virus DNA after peginterferon (Pegasys) treatment. As a result of active research on the serum titer as well as the loss of the surface antigen, it is a good treatment for hepatitis B. It is expected to be used as an indicator.

In this clinical study, the quantitative change of surface antigen and formation and loss of HBe antigen through long-term peginterferon alpha treatment in HBe antigen-

positive hepatitis patients whose hepatitis B virus DNA was measured as low as PCR sensitivity after long-term oral antiviral drug administration (Seroconversion).

Therefore, in this study, in patients with HBe antigen-positive chronic hepatitis B who are undergoing long-term nucleotide maintenance therapy, the change in surface antigens through 48 weeks of peginterferon administration was identified, and the relationship between HBe antigen formation and loss (serum conversion) was confirmed and used for oral use. I want to study how to stop the administration of antiviral drugs.

4. Research participation criteria:

To participate in this study, you must be 18 years of age or older, target HBe antigen-positive chronic hepatitis B patients who are undergoing long-term nucleotide maintenance therapy, and otherwise be healthy. Research officials will evaluate whether this study is right for you based on your medical history, physical examination, general safety laboratory tests (lab tests for blood), and the results of the screening process and other tests performed prior to the first drug administration.

5. Types of drugs used in this study

The drugs used in this study will be the following drugs, and their efficacy and safety have already been reported. The choice of drug is randomly selected in a 1:1 ratio between the following Pegasys and the conventional antiviral drug.

Pegasys

1) Drug/brand name: Peginterferon α2a /Pegasys®

2) Amount of drug substance/ingredient

① 1 Pre-filled syringe 180 (0.5 mL) contains 180 ug of the main ingredient

② 1 Pre-filled syringe 135 (0.5 mL) contains 135 ug of the main ingredient

③ 180 ug of main ingredient contained in 180 microgram proclick (0.5 mL)

④ Contains 135 ug of main ingredient in 135 microgram proclick (0.5 mL)

3) Formulation: Pre-filled syringe

① 1 pre-filled syringe 180

② 1 pre-filled syringe 135

③ 180 microgram pro click

④ 135 microgram pro click

4) How to use: Subcutaneous administration once a week

5) Pharmaceutical company/manufacturer: Roche Korea

Zeffix

1) Drug/brand name: Lamivudine/Zeffix®

2) Amount of drug substance/ingredient: Contains 100 mg per tablet

3) Formulation: Film coated tablet

4) How to use: Orally administered once a day

5) Pharmaceutical company/manufacturer: Glaxo Smith Kline

Baraclude

1) Drug/brand name: Entecavir/Baraclude®

2) Amount of drug substance/ingredient: Contains 0.5 mg per tablet

3) Formulation: Film coated tablet

4) How to use: Orally administered once a day

4) Pharmaceutical company/manufacturer: BMS Pharmaceutical Korea

Hepsera

1) Drug/brand name: adefovir/Hepsera®

2) Amount of drug substance/ingredient: Contains 10 mg per tablet

3) Formulation: Film coated tablet

4) How to use: Orally administered once a day

4) Pharmaceutical company/manufacturer: Glaxo Smith Kline

Levovir

1) Drug/brand name: clevudine/Levovir®

2) Amount of drug substance/ingredient: Contains 10 mg per tablet

3) Formulation: Hard capsule

4) How to use: Orally administered once a day

5) Pharmaceutical company/manufacturer: Bukwang Pharmaceutical

Viread

1) Drug/brand name: tenofovir/Viread®

2) Amount of drug substance/ingredient: Contains 300 mg per tablet

3) Formulation: Film coated tablet

4) How to use: Orally administered once a day

5) Pharmaceutical company/manufacturer: Gilead Science

6. What treatments are available if you participate in this study?

Participation in this study will be randomized (one place by throwing a coin to one place) and will be given a Pegasys or conventional oral antiviral drug. Medication is administered for 48 weeks in the Pegasys group, and blood tests are performed at regular intervals to determine treatment response. This study has no effect on your

diagnosis and treatment process, except that you are randomly selected from among drugs that have already been proven effective in the treatment of chronic hepatitis B. For reference, in the case of Pegasys, it is not recognized as a reimbursement in the domestic national insurance review standards under the current state.

7. Side effects of drugs used in this study

Some of the commonly known side effects of the drugs used in this study include: However, these side effects are rare and are not particularly common compared to the general population. To be prepared for possible, these side effects are monitored (continuous observation) together.

1) Pegasys

1. General symptoms: Fatigue, muscle pain, stiffness, local reaction at the injection site, asthenia, pain, influenza-like symptoms, boredom, drowsiness, tremor, fever flushing, weakness, herpes simplex. In particular, influenza-like symptoms (fatigue, fever, chills, loss of appetite, headache, joint pain, muscle pain, sweating, etc.) appear, and these symptoms are partially suppressed by acetaminophen. Usually, reducing the amount reduces the severity of side effects.
2. Digestive system: loss of appetite, nausea, vomiting, indigestion, diarrhea, abdominal pain, dry mouth, rarely constipation, abdominal bloating, stomatitis, glossitis, hyperkinesis, heartburn.
3. Liver: Changes in liver function such as elevated AST, ALT, ALP, LDH and bilirubin level can be observed.
4. Mental nervous system: dizziness, headache, sleep disturbance, nervousness, nervousness, insomnia, drowsiness, depression, decreased concentration, anxiety, etc. may appear.
5. Peripheral nervous system: Sometimes perception disorders, sensory loss, taste disorders, tingling, neurological disorders, tremors, etc. may appear.

6. Blood system: Leukocyte decrease, platelet decrease, red blood cell decrease, granulocyte decrease, hemoglobin and hematocrit decrease, anemia, etc. may occur.
7. Autoimmune phenomena: Symptoms thought to be caused by autoimmune phenomena [dysfunction of the thyroid gland (hypothyroidism or hyperthyroidism), deterioration of joint rheumatism, etc.] may occur.
8. Others: Sometimes weight loss, serum protein loss, injection pain, blood sugar rise, rheumatoid arthritis, etc. may occur.

2) Zeffix (Lamivudine)

1. In more than 10% of patients, symptoms such as headache and fatigue, as well as gastrointestinal symptoms such as nausea, diarrhea, and vomiting have been reported, and sometimes pancreatitis may occur. In addition, peripheral neuritis, paresthesia, and musculoskeletal pain have also been reported.
2. In 1-10% of patients, the central nervous system (dizziness, depression, fever, chills, insomnia), skin (rash), gastrointestinal system (anorexia, stomach abdominal pain, chest pain, increased amylase), hematology (neutropenia), liver (AST, ALT increase), nervous system and musculoskeletal system (muscle pain, joint pain), respiratory system (cough), etc. may be accompanied.
3. In less than 1% of patients, symptoms such as alopecia, anaphylaxis, anemia, hepatomegaly, hyperbilirubinemia, hyperglycemia, increased CPK, lactic acidosis, lymphadenomegaly, peripheral neuropathy, pruritus, rhabdomyolysis, weakness, thrombocytopenia, and gastritis. May be accompanied.

3) Baraclude (Entecavir)

1. In more than 10% of patients, there may be a temporary increase in liver levels (increased ALT).

2. In 1-10% of patients, symptoms of the central nervous system (headache, fatigue), endocrine and metabolic systems (hyperlipidemia), and digestive system (increased lipase, increased amylase, diarrhea, indigestion) may also be present.
3. In less than 1%, symptoms such as dizziness, hypoalbuminemia, insomnia, nausea, drowsiness, low thrombosis, and vomiting may be accompanied.

4) Hepsera (adefovir)

1. Asthenia was reported in over 10% of patients
2. Headache, abdominal pain, nausea, stomach and intestinal gas bloating, diarrhea, and indigestion were reported in 1-10% of patients.
3. In less than 1% of cases, myopathy and pancreatitis may be accompanied.

5) Levovir (clevudine)

1. >10%: infection (cold or upper respiratory tract infection), weakness, increased liver enzyme levels
2. 1-10%: abdominal pain, indigestion, headache, worsening hepatitis
3. <1%: fracture, rash, drowsiness, cellulitis, conjunctivitis, endometritis, gingivitis, gastric ulcer

6) Viread (tenofovir)

1. >10%: asthenia, pain, diarrhea, nausea
2. 1-10%: headache, abdominal pain, back pain, chest pain, fever, vomiting, loss of appetite, indigestion, bloating, pneumonia, depression, insomnia, speech
3. Super neuropathy, dizziness, rash reaction, sweating, muscle pain, weight loss

8. Protection of the confidentiality of research records

The information obtained from this study will be given only to the researcher. Your medical records and the consent you have signed may be shown only to researchers and participants on the research team for research and other purposes. However, your identifiable records will be kept confidential, and health information will be provided with your full name obscured, so the data does not tell you who you live or who you are. In addition, if the results of clinical studies are published, your personal information will be kept confidential.

You may revoke consent at any time by notifying the clinical research coordinator, in which case your medical information will no longer be available to the clinical research coordinator.

9. Costs associated with participation in this study

There are no additional costs to you by participating in this study. During the study period, you will only perform essential tests that are performed at the follow-up of hepatitis B patients, which is the basic test for disease progression and liver cancer screening regardless of participation in the study. Therefore, there are no additional costs incurred for the clinical trial. In this study, drugs that have already been proven effective in the treatment of chronic hepatitis B are used. If you participate in the study, you will receive free drugs if you are assigned to the Pegasys group according to randomization.

10. Compensation criteria for side effects

In this study, the investigator will comply with relevant laws and regulations and conduct a strict clinical trial in accordance with various related documents, recommendations, and suggestions.

The effectiveness of antiviral drugs compared to placebo in patients with advanced liver disease has already been established. Since data on the efficacy and safety of the drugs used in this study have already been reported, they are widely applied to the treatment of hepatitis B not only in Korea but also around the world.

11. Etc

1) Your participation in this study may be terminated by the Investigator at any time without your consent for the following reasons:

- When the investigator determines that it is necessary for your health and safety,
- When you did not follow the guidelines of the study,
- When the research team decided to discontinue the study, or for administrative reasons

2) If you experience adverse reactions or impairments that may be related to this study, or if you are making an unscheduled visit for medical treatment for any reason, if you have questions about the progress of this study, etc. In this case, please contact Prof. Jeong Heo (051-240-7869), who is in charge of this clinical trial, or Assistant Professor Hyun-Young Woo (051-240-7869), who is in charge of the trial. If you have any inquiries about your rights as a participant, please call the hospital's Institutional Review Board (051-240-7528) and contact.

3) Other matters necessary for the safety protection of subjects: This study is conducted in accordance with the clinical trial protocol approved by the clinical trial review committee after sufficiently reviewing the ethical and legal requirements of this study. Management standards) and the fundamental spirit of the Helsinki Declaration, which is the guidance of physicians in clinical research. Any violation of your human rights during this study will be notified to the Institutional Review Board or Health Authority.

Subject consent

Clinical Study Title: A Study on the Quantitative Changes of Hepatitis B Surface Antigens and the Relationship between HBe-antigen Seroconversion in 48-week Administration of Peginterferon in HBe-antigen-positive Chronic Hepatitis B Patients Under Long-Term Nucleotide Maintenance Therapy

Please read the information below and mark the boxes when you fully understand the contents.

☐ **I have read this agreement and fully understand its contents.**

☐ **I received detailed explanations from my doctor, asked questions if I had any questions, and received appropriate answers.**

☐ **I voluntarily participate in this study.**

☐ **I give permission to use and share my health information as described in this agreement.**

☐ **I may refuse or stop participating in the clinical study at any time during the clinical study period. Also, I understand that there will be no penalty for me if I stop participating in this study.**

☐ **I am free to request participation in clinical research and receive a copy of the consent form.**

Signature

To participate in this trial, you or your legal representative must sign and date the signature item.

Year Month Day

**Subject**	**Name**	**(signature)**
	**Address**	
**Investigator**	**Name**	**(signature)**

(If the following applies)

**guardian or agent**	**Name**	**(signature)**
	**Relationship**	
	**Address**	

5. Case record

6. Resume of principal investigator
