# Supplementary material for: Effect of switching from nucleos(t)ide maintenance therapy to PegIFN alfa-2a in patients with HBeAg-positive chronic hepatitis B: A randomized trial
Source: PLoS One. 2022 Jul 22;17(7):e0270716. doi: 10.1371/journal.pone.0270716 (PMC9307167; doi:10.1371/journal.pone.0270716)
Supplement: Attachment — Submitted filename: answer to reviewer(1).docx [file pone.0270716.s015.docx]

Please review your reference list to ensure that it is complete and correct. If you have cited papers that have been retracted, please include the rationale for doing so in the manuscript text, or remove these references and replace them with relevant current references. Any changes to the reference list should be mentioned in the rebuttal letter that accompanies your revised manuscript. If you need to cite a retracted article, indicate the article’s retracted status in the References list and also include a citation and full reference for the retraction notice.

As indicated, we have reviewed our reference list and confirm that it is complete and correct.

Additional Editor Comments (if provided):

Please respond sufficiently to the comments of the Reviewer 5.

Reviewer #5: The authors response make me quite confused than the original drafting.

1. I would suggest to add the limitation in their response to the reviewer into the limitation paragraph.

We have added the limitations mentioned in our response to the reviewer to the limitation paragraph in the manuscript.

2. As for their response to the reviewer 1 point 6 and reviewer 2 point 4, I think the discordance of the patients number and the statement makes me really confused about what happened actually to these patients. Would it be possible to make it clear? (Please find a native English speaker to check the fluency before re-submitting the response to the reviewers).

We are very sorry for the confusion. We did not provide sufficient explanation about Table 4 (now Table 2) in our first response to the reviewer. Table 4 (now Table 2) contained information only on cases with HBV DNA elevation that was newly developed at each time point during the study period. The data did not include cases where HBV DNA elevation developed at a previous time point. Therefore, the number of patients with HBV DNA>20,000 IU/mL could be larger than that of patients with HBV DNA>20 IU/mL or >2,000 IU/mL if the rescue antiviral therapy were delayed. We added the above explanation to the footnote of the table and added an additional column to the table to avoid such confusion. That additional column indicated the proportion of patients with HBV DNA level >20 IU/mL, 2,000 IU/mL and 20,000 IU/mL at each time point, excluding cases lost to follow-up or with rescue NA retreatment. This new column shows that the number of patients is more concordant across each HBV DNA level at each time point.

\## We also add the answer to previous comment 1 by reviewer #5

We added data on ALT kinetics during treatment for the PegIFN and NA groups to S2 Fig. Elevated ALT was observed from week 12 to week 48 in the PegIFN group, and the values for this group were significantly higher than those of the NA group (all p<0.001).
